# Application of Chiral Transfer Reagents to Improve Stereoselectivity and Yields in the Synthesis of the Antituberculosis Drug Bedaquiline

**DOI:** 10.1021/acs.oprd.3c00287

**Published:** 2023-10-13

**Authors:** Juliana M. S. Robey, Sanjay Maity, Sarah L. Aleshire, Angshuman Ghosh, Ajay K. Yadaw, Subho Roy, Sarah Jane Mear, Timothy F. Jamison, Gopal Sirasani, Chris H. Senanayake, Rodger W. Stringham, B. Frank Gupton, Kai O. Donsbach, Ryan C. Nelson, Charles S. Shanahan

**Affiliations:** † Medicines for All Institute, Virginia Commonwealth University, Richmond, Virginia 23284-3068, United States; ‡ R&D Centre, TCG Life Sciences Pvt. Limited, Kolkata, WB 700091, India; § Department of Chemistry, Massachusetts Institute of Technology, Cambridge, Massachusetts 02139, United States; ∥ TCG GreenChem, Inc., Richmond, Virginia 23219, United States

**Keywords:** chiral transfer, chiral lithium amide bases, lithiation, 1,2-addition, stereoselective, enantioselective, diastereoselective, bedaquiline (BDQ), bedaquiline assembly (BA) reaction, tuberculosis (TB), global health, API, LMICs

## Abstract

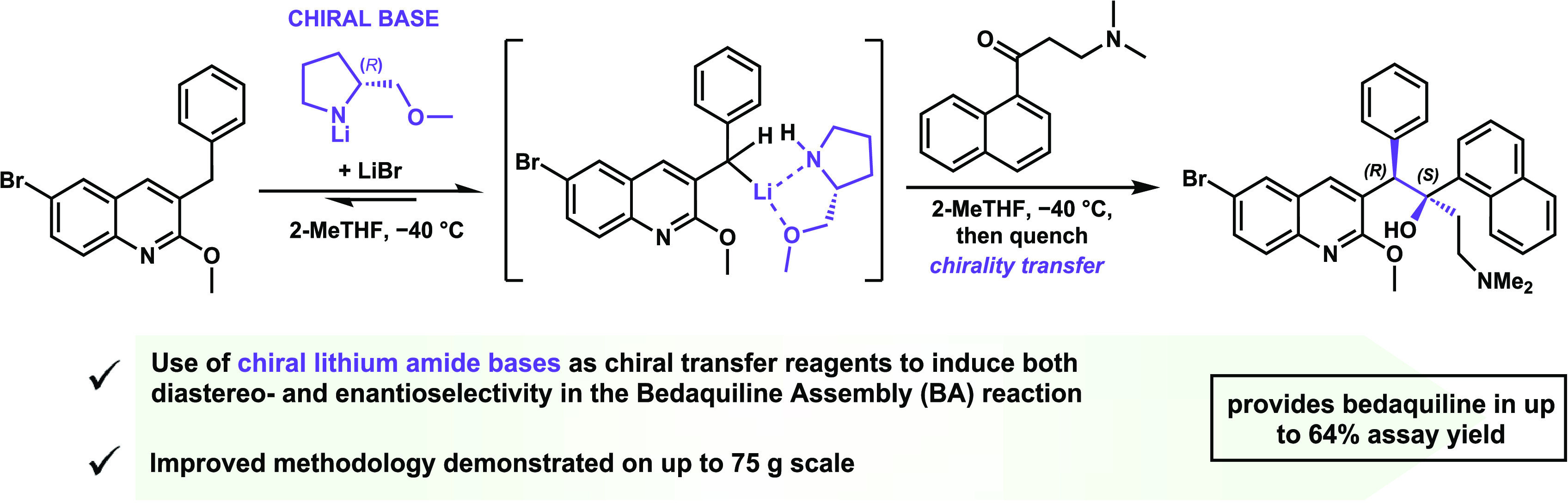

Bedaquiline (BDQ) is an important drug for treating multidrug-resistant tuberculosis (MDR-TB), a worldwide disease that causes more than 1.6 million deaths yearly. The current synthetic strategy adopted by the manufacturers to assemble this molecule relies on a nucleophilic addition reaction of a quinoline fragment to a ketone, but it suffers from low conversion and no stereoselectivity, which subsequently increases the cost of manufacturing BDQ. The Medicines for All Institute (M4ALL) has developed a new reaction methodology to this process that not only allows high conversion of starting materials but also results in good diastereo- and enantioselectivity toward the desired BDQ stereoisomer. A variety of chiral lithium amides derived from amino acids were studied, and it was found that lithium (*R*)-2-(methoxymethyl)pyrrolidide, obtained from d-proline, results in high assay yield of the desired *syn*-diastereomer pair (82%) and with considerable stereocontrol (d.r. = 13.6:1, e.r. = 3.6:1, 56% ee), providing BDQ in up to a 64% assay yield before purification steps toward the final API. This represents a considerable improvement in the BDQ yield compared to previously reported conditions and could be critical to further lowering the cost of this life-saving drug.

## Introduction

Tuberculosis (TB) is an infectious disease and global endemic caused by *Mycobacterium tuberculosis* bacteria.^1^ According to the World Health Organization (WHO), despite being a preventable and curable disease, TB caused a total of 1.6 million deaths in 2021 and represents the world’s deadliest infectious disease after briefly falling behind COVID-19 during the coronavirus pandemic period.^2^ To make matters worse, only about one in three people with this infection had access to treatment in 2020 due to its high cost, which presents a barrier to accessing these medicines in low- and middle-income countries (LMICs).^2^ Treatment courses can vary from 9 to 24 months, depending on the treatment regimen prescribed, which further exacerbates the cost of treatment, leading to poor treatment adherence and resulting in the emergence of significant multidrug-resistant TB (MDR-TB) rates.

Initially known as R207910 and TMC207, bedaquiline (BDQ) is a first-in-class diarylquinoline and an important oral medication used to treat adults with pulmonary MDR-TB. BDQ was developed by Janssen in 2005 and is now part of the WHO’s List of Essential Medicines.^3^ It was approved as an orphan drug by the U.S. Food and Drug Administration (FDA) in December 2012 under the accelerated approval program.^4^ Sold under the brand name Sirturo, BDQ fumarate salt is usually administered as a combination therapy and is mandated to be used only in patients who do not have other treatment options.^5^ In August 2019, the FDA approved the BPaL regimen, which was developed by the Global Alliance for TB Drug Development (TB Alliance) and consisted of a 6-month oral treatment regimen composed of BDQ (B), pretomanid (Pa), and linezolid (L) for treating extensively drug-resistant tuberculosis (XDR-TB). Alternative XDR-TB treatments require a 20-month treatment course and a combination of at least seven different drugs, which consequently results in an increase in the overall treatment cost, making BPaL a promising option for patients with this need.^6^

BDQ possesses a novel mechanism of action via the inhibition of a critical enzyme responsible for adenosine triphosphate (ATP) synthesis. The lack of efficient energy production by the bacterial cells leads to the inhibition of mycobacterial growth and ultimately results in its death.^7^ The bulk of the previously available anti-TB drugs acts by inhibiting the synthesis of the cell wall or affecting the bacteria’s genetic material replication and transcription process.^8^ In this sense, when compared to the alternatives in the market, the discovery of BDQ is considered a breakthrough for TB treatment, breaking the hiatus of 40 years without the development of a new TB drug targeting a different point of the *M. tuberculosis* lifecycle.

**Scheme 1:**
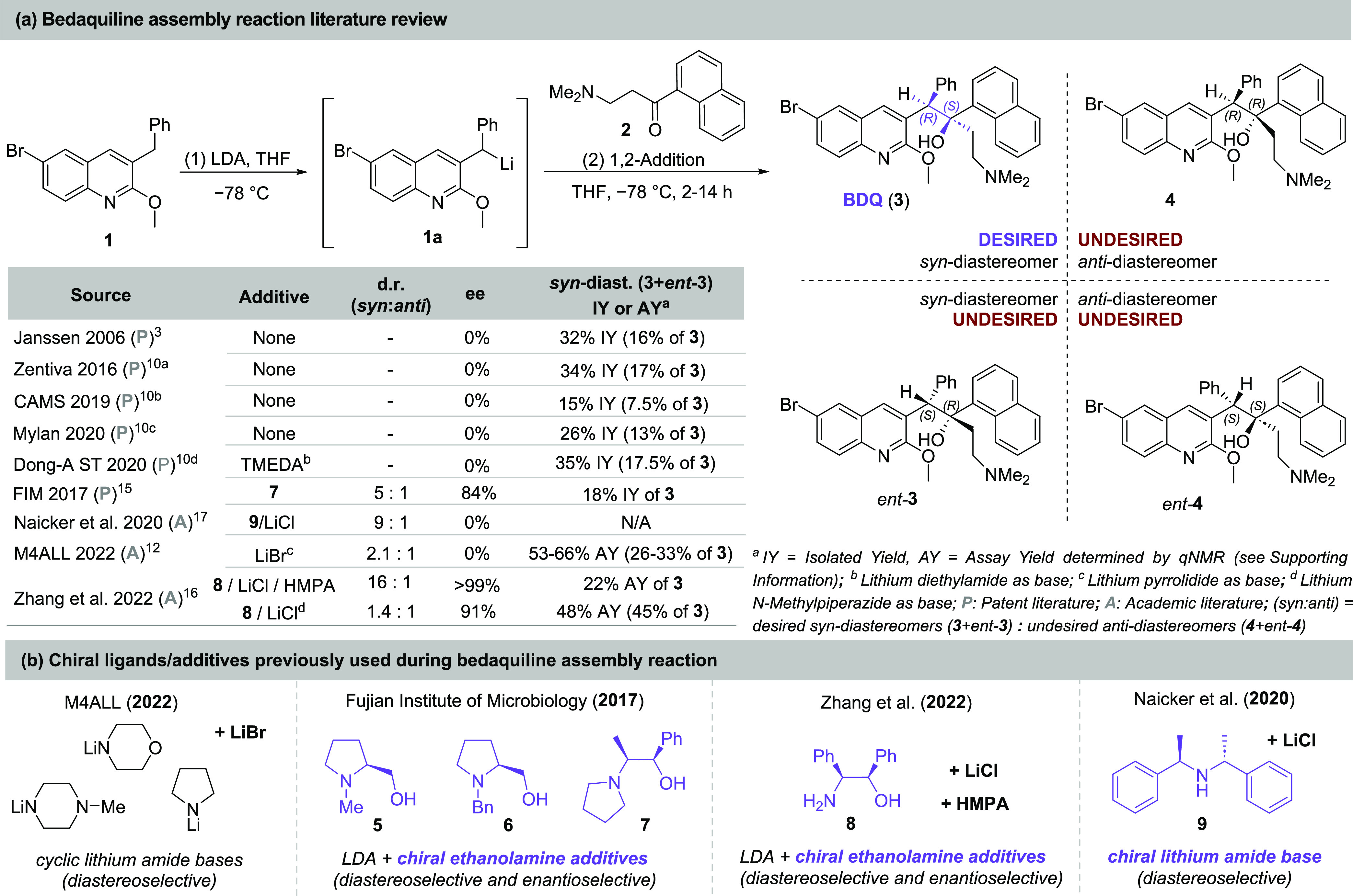
Overview of the BDQ (**3**) Synthetic Methods Previously Described in the Literature

The current manufacturing process for BDQ relies on the reaction of quinoline derivative **1** with ketone **2** (Scheme 1a). The bedaquiline assembly (BA) reaction couples lithiated quinoline **1a** with ketone **2** via a 1,2-addition to form products **3**, *ent*-**3**, **4**, and *ent*-**4**. This mixture of four stereoisomers is distributed in two pairs of diastereomers, *syn*-(*RS*, *SR*) and *anti*-(*RR*, *SS*). BDQ is the (1*R*,2*S*) stereoisomer **3**, the most active against TB.^9^ It is important to acknowledge that the other isomers present reduced activity toward the bacteria. Lesser activity is observed if BDQ (**3**) is combined with its enantiomer *ent-***3**, evidencing the importance of having an efficient purification process that produces the enantiopure active pharmaceutical ingredient (API).^9^

Isolation of BDQ (**3**) from the complex mixture obtained in the 1,2-addition step is achieved through a 4-step sequence of crystallizations (see Supporting Information Scheme S1), which includes precipitating out the less soluble *anti*-diastereomer pair (**4** and *ent-***4**), precipitation of the desired *syn*-diastereomer pair (**3** and *ent-***3**) to remove unreacted starting materials, chiral resolution with (*R*)-BINOL-phosphoric acid and treatment of the obtained solid with a base to yield enantiopure BDQ (**3**), and a final fumarate salt formation and final recrystallization to yield the API BDQ (**3**) fumarate. This long purification process results in a considerable loss of material and is required due to the low conversion and stereoselectivity of the 1,2-addition.

Most of the relevant literature describing the BA reaction does not provide experimental data on the completion of the purification process all the way to the desired API, the BDQ (**3**) fumarate salt. In the cases where this data was displayed, BDQ (**3**) fumarate isolated yield was no more than 10% (see the Supporting Information, Table S1).^3,10^ An additional drawback to this methodology that also contributes to lowering the yield of the product is the low conversion of starting materials **1** and **2** (40–70% remains unreacted in the reaction mixture).^10^ The remaining starting materials can be recovered after the 1,2-addition, and a higher recovery percentage can be achieved by treating the undesired stereoisomers *ent-***3**, **4**, and *ent-***4** with base, which promotes the retro-addition toward **1** and **2**.^11^ Nevertheless, there is no available literature showing how recovered quinoline **1** and ketone **2** can be separated at scale without the use of chromatography. Thus, practical limitations prevent these low-yielding processes from being economical due to the considerable loss of material through the process. The low BDQ (**3**) yield results in a significant waste of material, driving up the total cost of the product. Thus, methods to improve this 1,2-addition step would significantly impact the API manufacturing cost.

The M4ALL has recently reported a significant improvement in the overall yield of this coupling step.^12^ It was proven that higher conversion could be achieved by replacing LDA with less hindered or stronger lithium amide bases obtained from pyrrolidine, morpholine, or *N*-methylpiperazine (Scheme 1b). This modification in the methodology provided a substantial increase in the yield of the mixture of the *syn*- and *anti*-diastereomers (**3** + *ent-***3** + **4** + *ent-***4**) (78–97% assay yield). Furthermore, the use of lithium bromide (LiBr) as an additive improved the *syn*/*anti*-diastereomeric ratio (d.r.) from 1:1.2 to 2.1:1 (Scheme 1a,b).

Only a few examples of the asymmetric synthesis of BDQ (**3**) making use of different strategies and modified starting materials are available in the literature.^13^ Although innovative from a chemistry point of view, these routes present a higher step count and cost compared to Janssen’s methodology and, unfortunately, still provide low overall yields. These factors summed to the fact that these alternatives were not further optimized and validated on a large scale, decrease the chances of these routes being readily adopted by BDQ (**3**) fumarate manufacturers. The use of chiral transfer reagents, such as chiral auxiliaries, chiral solvents, or chiral bases, has found broad applications in organic synthesis whereby stereochemical information is transferred from a chiral to an achiral species and is incorporated into the product.^14^ These strategies can represent an important alternative for the asymmetric BA reaction. Even when stoichiometric amounts of chiral transfer reagents are required, these tactics can often be economically favorable when the materials are derived from commodity chemicals or recycled from the process.

In this regard, the Fujian Institute of Microbiology (FIM) and Zhang et al. described the use of chiral ethanolamines (**5**, **6**, **7**, and **8**) in combination with LDA in the BA reaction (Scheme 1a,b).^15,16^ The best stereoselectivity was achieved when employing the asymmetric compound **8**, which provided a 16:1 d.r. and >99% ee in favor of the desired BDQ (**3**) stereoisomer. However, under these conditions, the coupling reaction suffers from low conversion and approximately 75% of starting material remains unreacted. The combination of additive **8** with a less hindered lithium amide base, such as lithium *N*-methylpiperazide, improved conversion (83%) at the expense of stereoselectivity (*syn*/*anti*, 1.4:1 d.r., 91% ee). The combination of these effects resulted in a 45% yield of the desired isomer **3** in the crude mixture. Naicker et al. also reported an asymmetric approach for the BA reaction (Scheme 1a,b). In this case, the use of the chiral lithium amide base obtained from (*R*)-bis((*R*)-1-phenylethyl)amine (**9**) was studied. Despite some diastereoselectivity being achieved (9:1 d.r., *syn*/*anti*), conversion was extremely low (33% by HPLC A%), and no enantioselectivity was observed.^17^

Herein, we report the use of chiral lithium amide bases derived from amino acids as affordable chiral transfer reagents to greatly improve the reactivity and stereoselectivity of the BA reaction currently being used to manufacture BDQ (**3**) fumarate for TB patients worldwide.^18^ In general, the transfer of chirality in reactions involving highly reactive intermediates, such as the lithiated intermediate **1a**, offers even more challenge for stereochemical control because the background achiral reaction is often hard to slow down. This is the case with the BA reaction and why chiral transfer using chiral bases has been the preferred approach in this work.^19^

## Results and Discussion

### Initial Chiral Ligand Screening

**Scheme 2:**
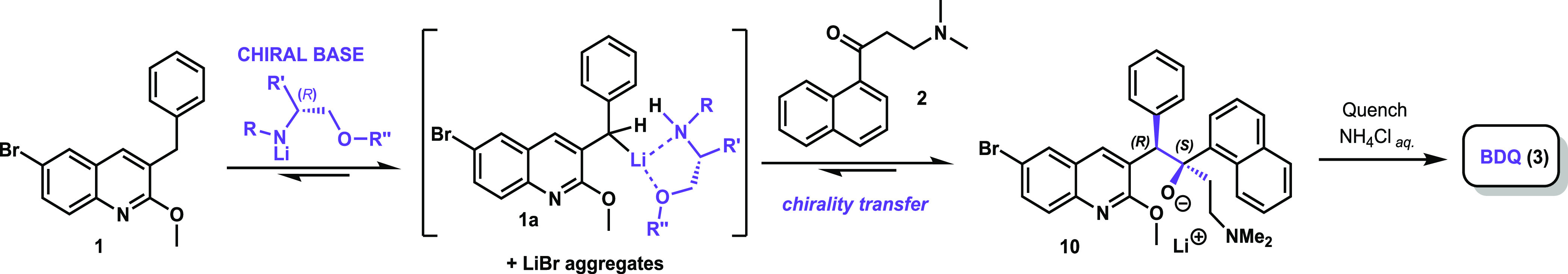
Possible Mechanism for Chirality Transfer via the Formation of Lithium Complex Intermediates

The review of the previously discussed literature on the asymmetric synthesis of BDQ (**3**) suggests that chiral ethanolamines (containing a N–C–C-O bond structure) can promote significant stereoinduction via the transfer of chirality in the 1,2-addition step (Scheme 2). While the exact transition state for this chirality transfer is unknown, the impact of additives like LiBr seems to suggest that higher-order aggregation is critical for the associated chiral amine to promote stereoselectivity in this reaction.^20^ Indeed, lithium salt additives influence diastereoselectivity during lithiation reactions and are capable of affecting the geometry, equilibrium, and rate of assembly or dissociation of lithium aggregates.^21^ These early examples encouraged us to further investigate the use of chiral lithium amide bases possessing a chiral ethanolamine substructure to invoke chiral induction during BDQ (**3**) synthesis. Herein, the dual role of lithium chiral amide bases (N–C–C–O bond structure) was demonstrated for the first time in the BA reaction. It not only promotes quinoline **1** deprotonation but also simultaneously acts as a chiral ligand to induce both diastereo- and enantioselectivity during the 1,2-addition reaction.

**Scheme 3:**
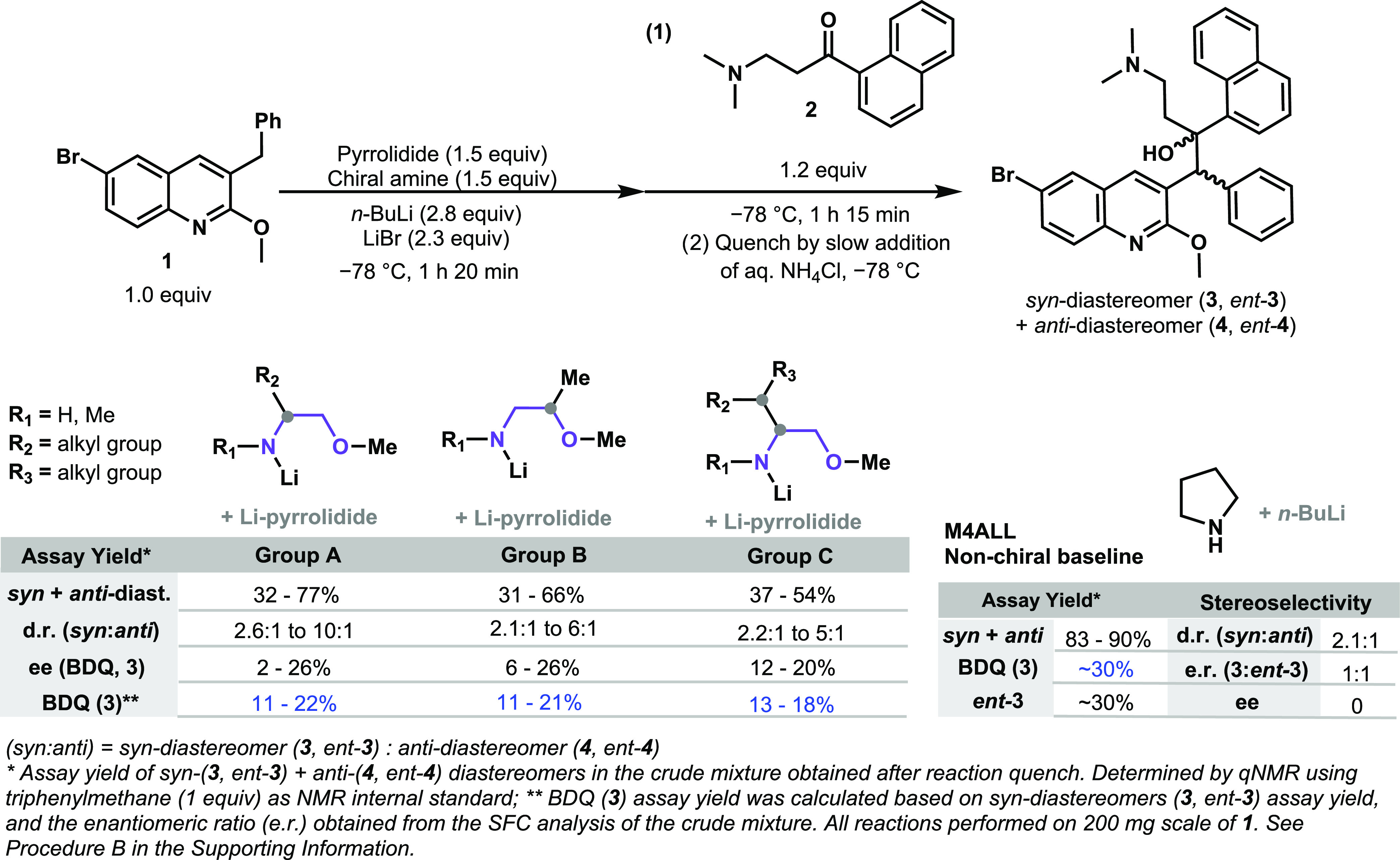
Overview of the Reaction Outcome When Chiral Ethanolamines Derived from Amino Acids Were Combined with Lithium Pyrrolidide Base

In seeking an effective chiral transfer agent, approximately 25 chiral amines containing the aforementioned ethanolamine substructure were screened; the majority of them were derived from d/l-amino acids (alanine, leucine, isoleucine, threonine, valine, and proline) (see the Supporting Information, Scheme S10 for the complete list). At first, the chiral amines were used in combination with lithium pyrrolidide as the base for the lithiation step (Scheme 3). All of the screened chiral amines afforded some level of influence on stereoselectivity toward BDQ (**3**) or *ent-***3**, ranging from 1.5:1 to 10:1 d.r. (*syn*/*anti*) and overall assay yields from 30 to 78%. Moreover, the enantioselectivity toward BDQ (**3**) varied from 2 to 26% ee.

The average assay yield of BDQ (**3**) in these small-scale tests was slightly lower (<20%) than our earlier achiral variant (∼30%); however, several key trends were observed that suggest an improved system may be within reach. First, the role of LiBr in promoting diastereoselectivity was already expected; however, for all chiral ligands tested, the *syn*-diastereomer pair, **3** and *ent-***3**, was favored over the *anti*-pair, **4** and *ent-***4**. Second, all chiral amines derived from natural l-amino acids possessing one chiral center (groups A and B, Scheme 3) favored *ent-***3**, with the only exception being l-threonine, while the non-natural d-amino acid derivatives favored BDQ (**3**) (see Supporting Information Scheme S10).

### Use of Chiral Lithium Amides to Promote Quinoline 1 Deprotonation

**Scheme 4:**
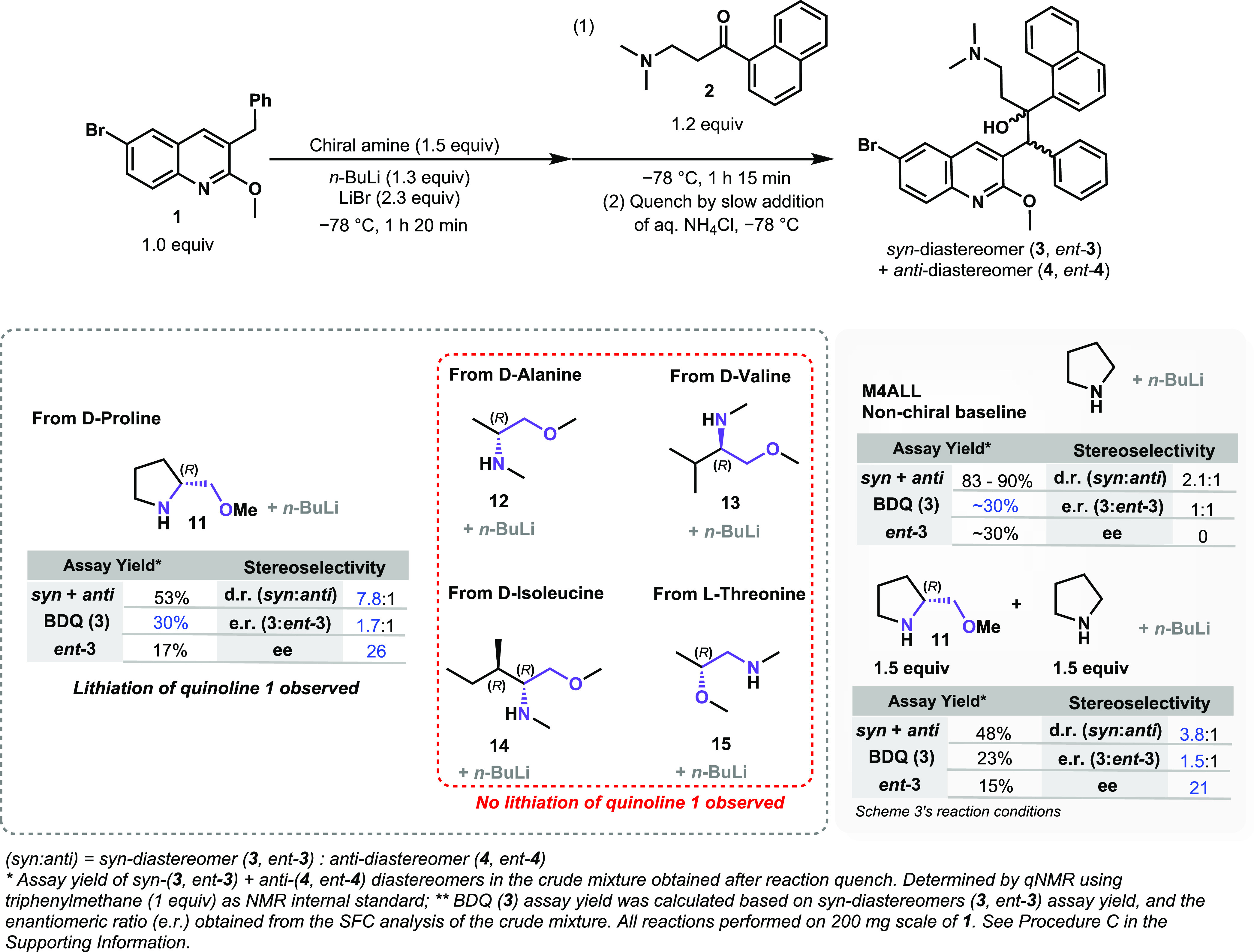
Chiral Lithium Amides That Promote Enantioselectivity toward BDQ (**3**) and Quinoline **1** Deprotonation

During the aforementioned screening (Scheme 3), it was found that the ethanolamines **11**–**15** resulted in the highest enantioselectivity, providing BDQ (**3**) in 20–26% ee (see Supporting Information Scheme S10). These chiral amines were re-evaluated in the absence of lithium pyrrolidide; the objective being to analyze if the lithium chiral amides obtained from those molecules would be basic enough to promote quinoline **1** deprotonation by themselves (Scheme 4). The acyclic lithium amides derived from **12**–**15** did not show any reaction in the absence of an external base. Notably, the lithium amide base of (*R*)-2-(methoxymethyl)pyrrolidine (**11**) promoted quinoline **1** lithiation and afforded a 53% assay yield of the *syn* + *anti*-diastereomers, in addition to the d.r. and enantiomeric excess (ee) favoring BDQ (**3**) (*syn*/*anti*, ∼ 8:1 and 26% ee, respectively) (Scheme 4). Despite the fact that base **11** yielded only 30% of BDQ (**3**), the same yield as in the M4ALL’s nonchiral approach, it is important to highlight that this constitutes the first example of the usage of chiral lithium amide bases directly to promote both diastereo- and enantioselectivity in the current BA reaction. For this reason, amine **11** was selected for further optimization studies. Our main goal was to analyze if modifications in the reaction conditions would allow a higher starting material conversion and consequently increase the yield of BDQ (**3**).

### Reaction Cost Optimization with Lithium (*R*)-2-(Methoxymethyl)pyrrolidide (**11**)

**Scheme 5:**
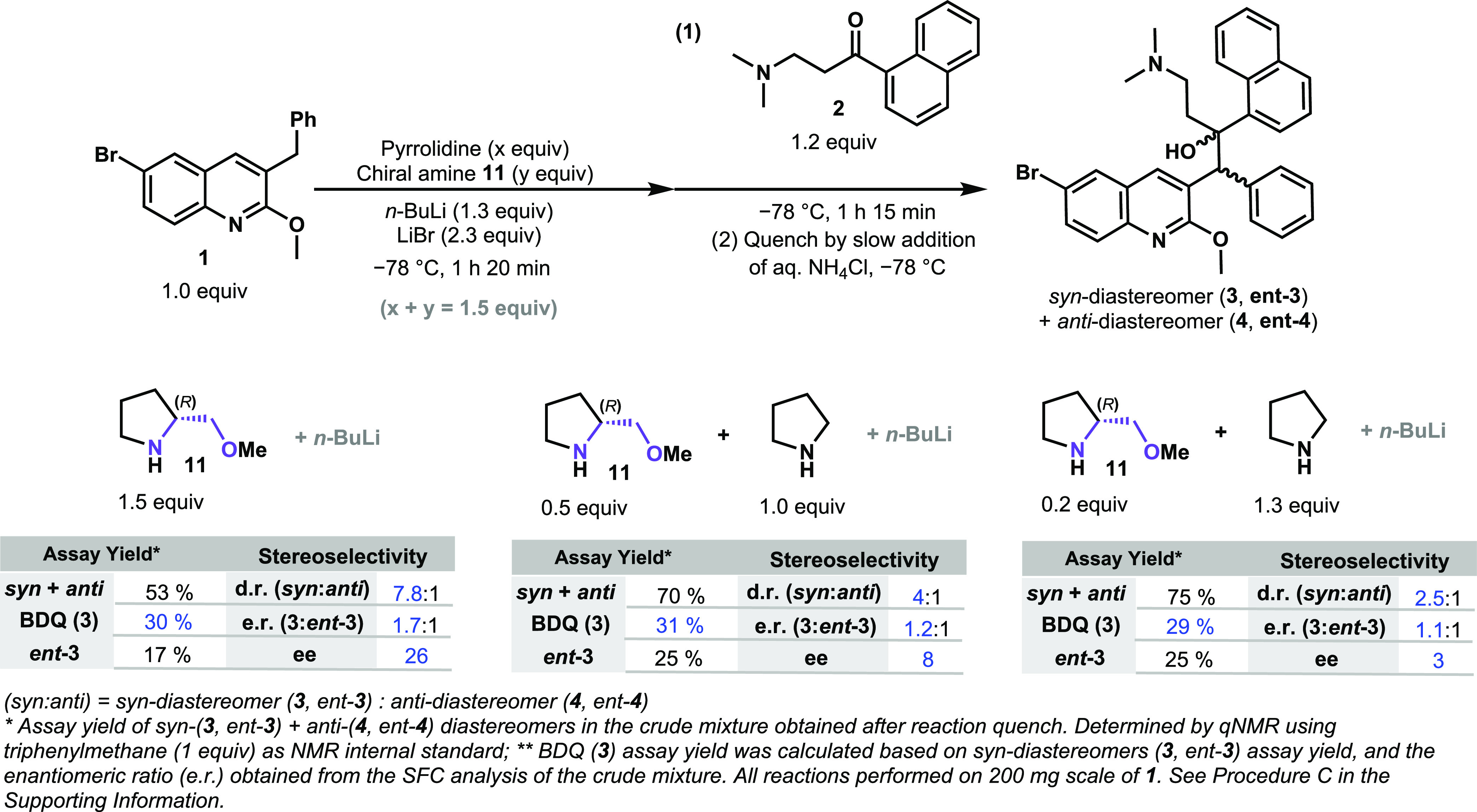
Decrease of the Equivalent Amount of Chiral Base **11** While Increasing Lithium Pyrrolidide

With the focus on lowering the cost of this synthetic route, the usage of a substoichiometric amount of the chiral reagent was explored. Therefore, a decrease in the molar equivalents of **11** was investigated (from the initial 1.5 equiv), and pyrrolidine was examined as a cobase to offset the lower amount of **11** (Scheme 5). In this case, the sum of equivalents for both amines was fixed at 1.5 equiv as the amount of **11** was sequentially lowered. Higher yields of the *syn* + *anti*-diastereomer mixture were observed when the amount of lithium pyrrolidide was increased from 0 to 1.3 equiv (from 53 to 75%, respectively). Nevertheless, a substantial deterioration of enantioselectivity was observed when the amount of chiral base **11** was reduced from 1.5 to 0.2 equiv. These experiments unfortunately concluded that the use of catalytic amounts of the chiral transfer reagent **11** would not be feasible. Indeed, the previously cited literature on the use of chiral lithium alkoxides during the BA reaction also described the employment of excess of the chiral component (1.1–2 equiv) relative to quinoline **1**, which corroborates our observation and led us to investigate the impact of other process parameters on the reaction outcome.^15,16^

### Increasing the Reaction Temperature

**Scheme 6:**
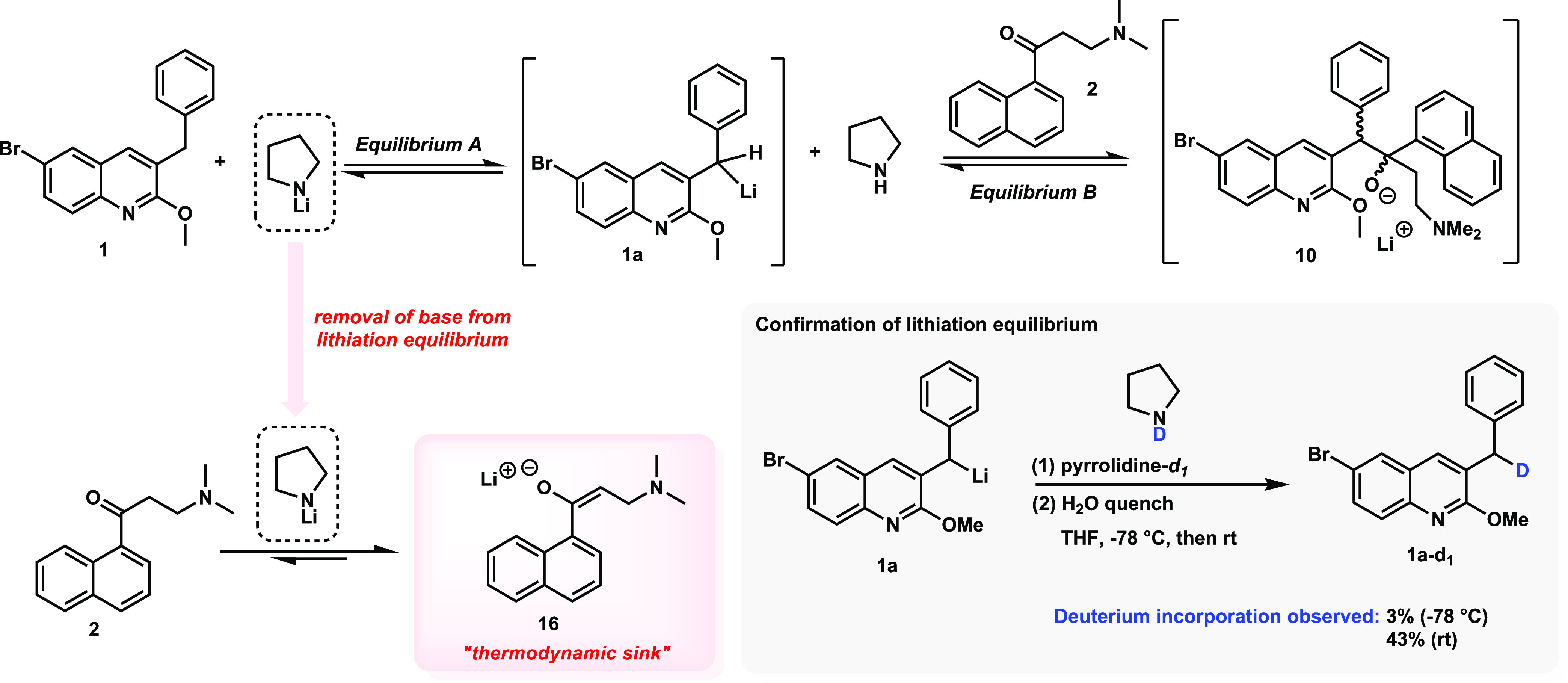
Reversibility of Lithiation Reaction and Equilibrium Shift toward Enolate **16** during Temperature Increase

The effect of temperature on the BA reaction with nonchiral bases has been previously reported.^12^ It was observed in those studies that carrying out the reaction at temperatures higher than −78 °C strongly favored the retro-addition of the lithium alkoxide **10**, leading the reaction equilibrium to favor **1a** and **2** (Scheme 6, Equilibrium B). Although the 1,2-addition reaction is reversible, once the temperature is increased, other undesired side reactions begin to occur, further driving the reaction equilibrium away from the addition adduct **10**, even if attempts are made to restore the reaction temperature to −78 °C. When pyrrolidine-*d*_1_ was added to intermediate **1a**, deuterium incorporation into quinoline **1a** was observed, especially at higher temperatures (Scheme 6). This experiment, along with other sets of deuterium labeling experiments (see the Investigating the Lithiation Step Mechanism section in the Supporting Information), evidence that the initial lithiation step is also under a reversible equilibrium (Scheme 6, Equilibrium A). In this way, as the retro-addition proceeds, the concentration of **1a** increases in solution, and the original lithium amide base is reconstituted by the reaction equilibration. Thus, the formation of enolate **16** begins to disrupt the equilibrium in an irreversible manner since the enolization of **2** is favored at higher temperatures and represents a thermodynamic sink for this reaction.

**Scheme 7:**
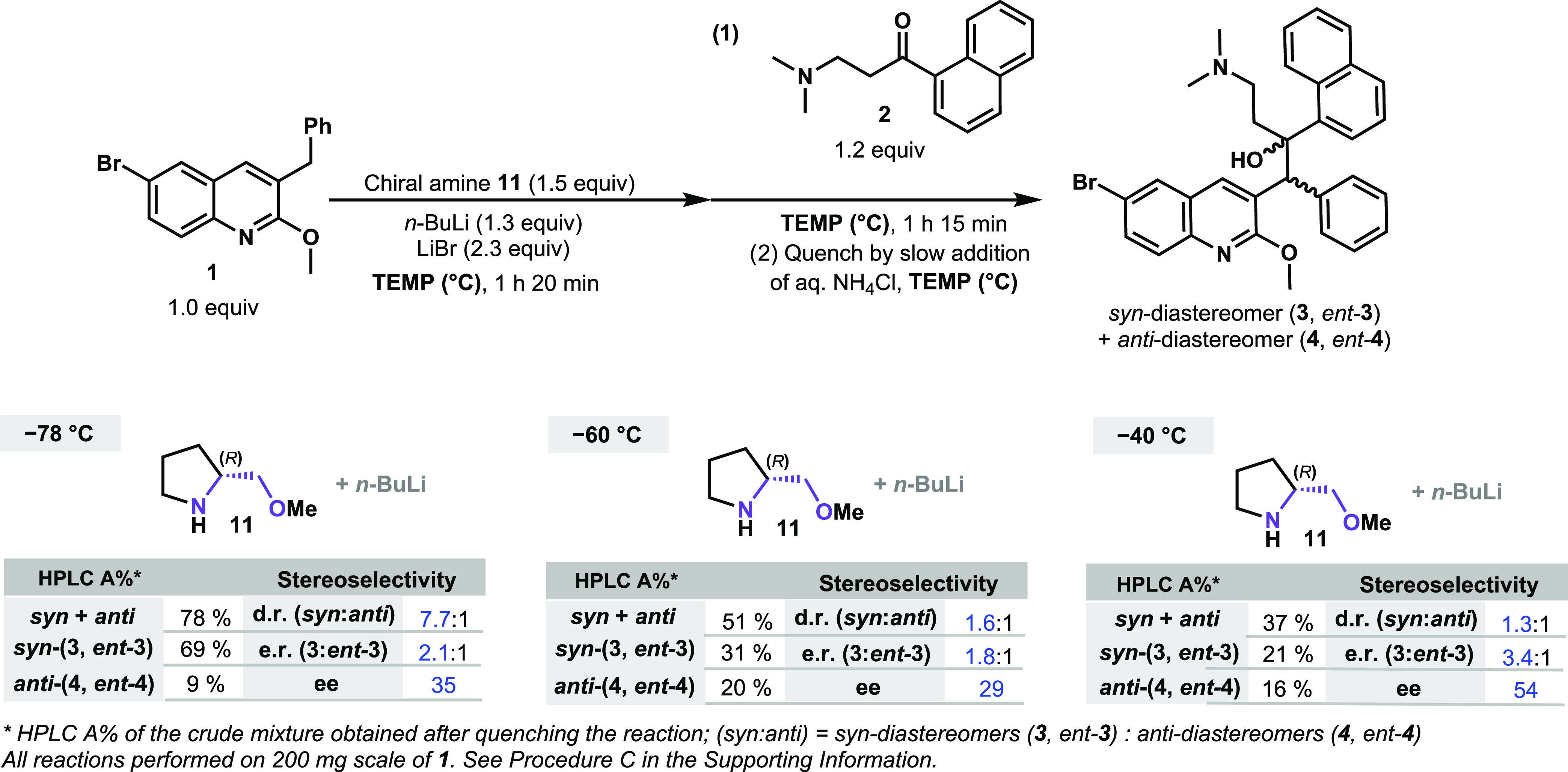
Comparison of the Reaction Outcome at Different Temperatures

Higher reaction temperatures were nevertheless attempted for the asymmetric approach using the chiral lithium amide obtained from **11** (Scheme 7). In this study, we observed that the reactions performed at higher temperatures resulted in lower diastereoselectivity and conversion. The area percent analysis by liquid chromatography (HPLC A%) of the quenched crude reaction mixture showed that the *syn*-diastereomer pair, **3** and *ent-***3**, was obtained in only 31% at −60 °C (1.6:1 d.r.) and 21% at −40 °C (1.3:1 d.r.), compared to 69% at −78 °C (7.7:1 d.r.). Although the d.r. and conversion were negatively influenced, the effect on the enantioselectivity was surprisingly inverted, and the reaction at −40 °C provided higher enantioselectivity (54% ee) compared to the reaction at −78 °C (35% ee).

These results seem to suggest that the equilibrium described in Scheme 2 strongly impacts the coordination of lithiated quinoline species **1a** with the chiral transfer reagent, and these provoked changes result in the enhanced enantioselectivity toward BDQ (**3**). While this result was surprising, the unwanted retro-addition and ketone **2** enolization issue needed to be addressed to ensure good overall starting material consumption. We decided to explore if the use of different solvents could allow the course of this reaction at −40 °C while keeping high conversion rates of the starting materials **1** and **2**, and thus, we turned our attention there next.

### Exploring the Use of Different Solvents

**Figure 1:**
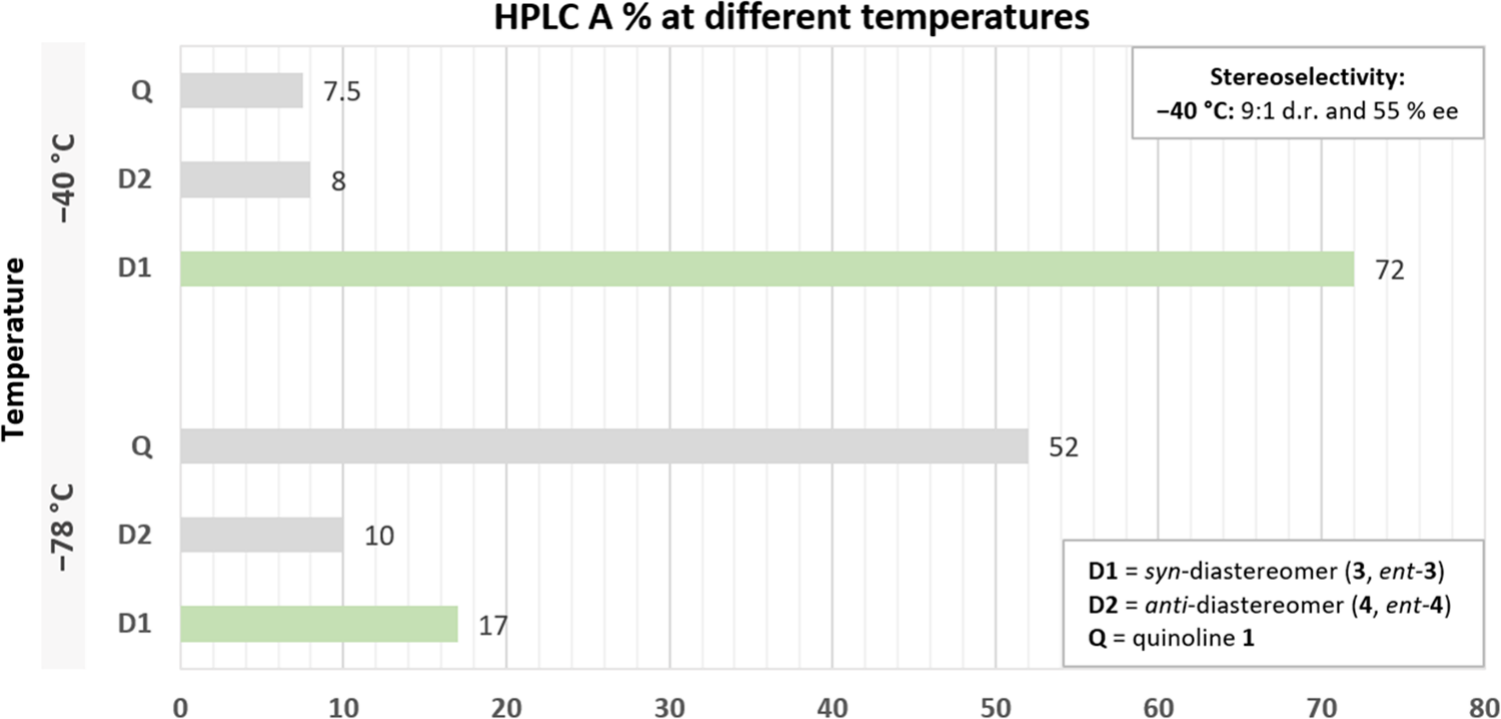
Effect of temperature on the 1,2-addition reaction in 2-MeTHF. Reaction conditions (500 mg scale of **1**): Lithiation: 1 h, quinoline **1** (1.0 equiv, 5 V), and chiral amine **11** (1.5 equiv), LiBr (2.3 equiv), *n*-BuLi (1.8 M, 1.3 equiv) in 5 V of solvent. 1,2-Addition: 1 h, 40 min, ketone **2** (1.2 equiv, 5 V). HPLC A% was obtained after reaction quench with 25% solution of NH_4_Cl (see Supporting Information General Procedure D).

Various solvents were evaluated for this transformation, albeit considerable constraints exist in solvent selection with this system to ensure compatibility with the strong bases and solubility of the reaction mixture at low temperatures. Nonpolar solvents, for example, are not compatible due to the low solubility of LiBr, which is required to encourage aggregate formation of the ionic intermediates and good stereoselectivity. To that end, the reaction was attempted using toluene as the solvent in the absence of LiBr, and mostly starting materials (>85%, HPLC A%) were observed after the reaction quench. Binary solvent systems containing a nonpolar and polar solvent pair were also analyzed (e.g., 1:1 toluene/2-MeTHF, 1:1 hexanes/2-MeTHF, 1:1 DCM:THF) in the presence of LiBr; however, similarly poor conversion was observed, with only trace amounts of product being formed. The replacement of the standard solvent THF with the less polar 2-MeTHF led to the formation of the product, albeit at a slower rate of reaction. When the reaction in 2-MeTHF was performed at −78 °C, starting materials **1** and **2** were still the major components of the reaction mixture (Figure 1). This indicates that the reaction in 2-MeTHF is slower than in THF since the retro-addition is not likely at this temperature. When the reaction was carried out at −40 °C, improved conversion was achieved, and 72% of the *syn*-diastereomer pair, **3** and *ent-***3**, was observed (HPLC A%) along with a 9:1 d.r. (*syn*/*anti*). As observed for the reaction in THF at −40 °C, the temperature increase in the 2-MeTHF system also improved the enantioselectivity to 55% ee.

**Figure 2:**
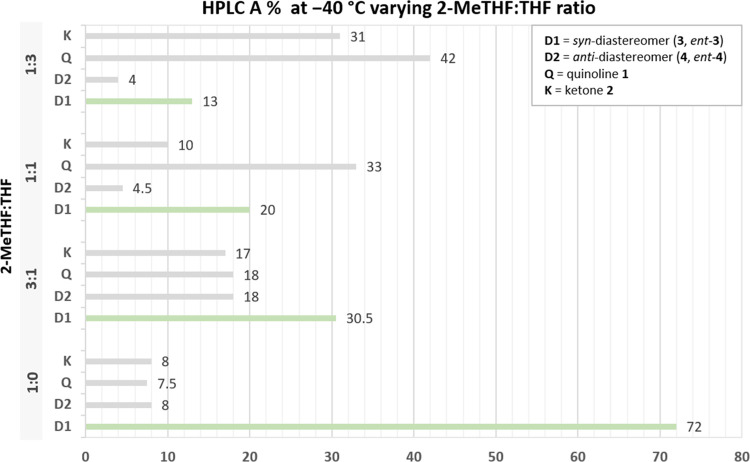
Effect of 2-MeTHF/THF ratios on the 1,2-addition reaction at −40 °C. Reaction conditions (500 mg scale of **1**): Lithiation: 1 h, quinoline **1** (1.0 equiv, 5 V), chiral amine **13** (1.5 equiv), LiBr (2.3 equiv), *n*-BuLi (1.8 M, 1.3 equiv) in 5 V of solvent. 1,2-Addition: 1 h and 40 min, ketone **2** (1.2 equiv, 5 V). HPLC A% obtained after reaction quench with 25% solution of NH_4_Cl (see Supporting Information General Procedure D).

Different ratios of a binary mixture of 2-MeTHF/THF were also screened at −40 °C (Figure 2). The addition of a small amount of THF (25%) to 2-MeTHF (3:1 2-MeTHF/THF) was enough to reduce the *syn*-diastereomer pair (**3** and *ent-***3**) amount by more than half, from 72 to 30% (HPLC A%). The higher the THF percentage in this binary mixture, the more the equilibrium favors the retro-addition and enolization of **2**. The use of 2-MeTHF alone was found to provide superior conversion of the starting materials. This result was considered to be very promising, as performing the BA reaction at an increased temperature would offer practical improvement to throughput due to the enhanced enantioselectivity toward BDQ (**3**) as well as ease operational issues of cryogenic reactions for manufacturers.

### Effect of the Reaction Time

Given the thermodynamics at play in the BA reaction, we turned our attention to the effect of reaction time on the equilibria as we had explored in our previous work.^12^ For the nonchiral approach using lithium pyrrolidide, lithiation time did not seem to have a significant impact on the reaction outcome and no major side reactions were observed at lower temperatures. Deprotonation of **1** is usually very fast (<15 min), and when lithiation was monitored for 90 min, the mass balance was always higher than 95% for all time points. The same observation held true for chiral lithium amide **11**. With regard to the 1,2-addition, it was found that the reaction time is critical to avoid the undesired retro-addition and ketone **2** enolization. For our nonasymmetric approach, the longer the reaction was carried out (>30 min after the completion of ketone **2** addition), the more the reaction was dominated by the thermodynamically driven enolization, leading to the deterioration of the overall yield.

The reaction time for the 1,2-addition step was also analyzed for the asymmetric system. In this case, the ketone **2** addition time was fixed at 1 h, while the time after the addition of **2** was varied from 5 to 60 min, followed by the quenching of the reaction mixture (Table 1). At the shorter reaction time of 5 min, the overall assay yield of the *syn* + *anti* diastereomer mixture was good (81%). However, the reaction was likely not able to reach equilibrium and suffered from lower stereoselectivity (9:1 dr and 19% ee), resulting in a 43% assay yield of BDQ (**3**) (Table 1, entry 1). As the reaction time was increased (10–30 min), a considerably higher stereoselectivity was achieved (up to 15:1 d.r. and ∼50% ee) without noticeably sacrificing conversion (Table 1, entries 2–4). The lower overall yield of 69% at 20 min did not reflect the trend, and this outlier might serve to highlight the high sensitivity of the reaction (e.g., moisture in the system or unintended increase in temperature during reaction quench). At a longer reaction time of 60 min, a decrease in d.r. (9:1) was observed, leading to the conclusion that 20–30 min of postcompletion of ketone **2** addition corresponds to the optimal reaction time. Under these optimized conditions, BDQ (**3**) was synthesized with high levels of stereoselectivity, and it was ultimately achieved in a 56% assay yield (Table 1, entry 4).

**Table 1:** Influence of Reaction Time after Completion of Ketone **2** Addition^a^

entry	time (min)	assay yield (**3** + *ent-***3** + **4** + *ent-***4**) (%)	*syn* (**3** + *ent-***3**) (%)	*anti* (**4** + *ent-***4**) (%)	d.r. (*syn*/*anti*)	e.r. (**3**:*ent-***3**)^b^	ee (%)	assay yield BDQ (**3**) (%)^c^
1	5	81	73	8	9:1	1.5:1	19	43
2	10	80	74	6	12:1	2.7:1	46	54
3	20	69	64	5	13:1	3.7:1	57	50
4	30	80	75	5	15:1	3:1	50	56
5	60	77	69	8	9:1	3.6:1	56	54

a Reaction conditions (2-MeTHF, −40 °C, 1.0 g scale of quinoline **1**): Formation of lithium amide base (step 1): 20 min at −20 to −30 °C, chiral amine **11** (1.5 equiv), LiBr (2.3 equiv), *n*-BuLi (1.8 M, 1.3 equiv) in 10 V of solvent. Lithiation (step 2): 1 h, quinoline **1** (1.0 equiv) in 5 V. 1,2-Addition (step 3): 65–105 min, ketone **2** (1.2 equiv) in 5 V. Reaction quench with 25% aqueous solution of NH_4_Cl. (See Supporting Information General Procedure D).

b Enantiomeric ratios (e.r.) were obtained from SFC analysis.

c BDQ (**3**) assay yields based on the purity of crude mass obtained after reaction quench, determined by HPLC wt %.

### Understanding the Impact of Concentration on the Reaction Outcome

**Figure 3:**
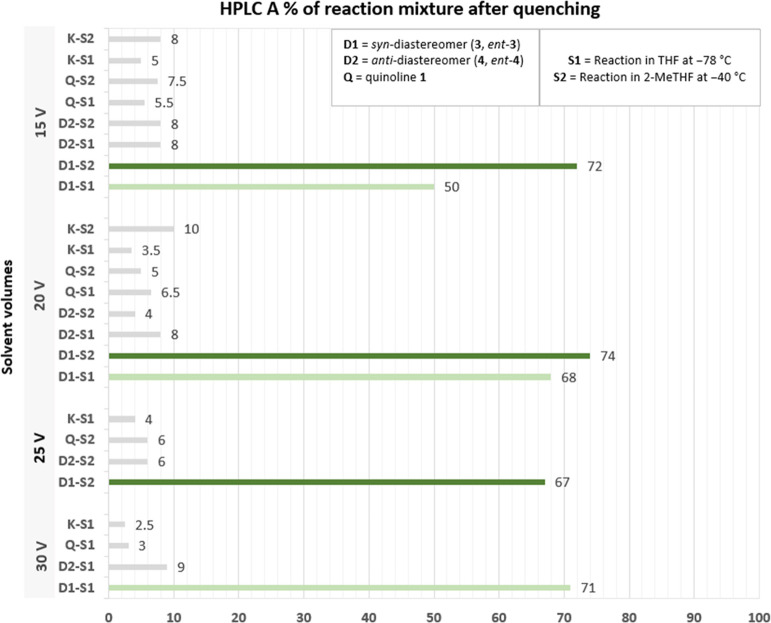
Variation of reaction concentration in THF at −78 °C and 2-MeTHF at −40 °C. Reaction conditions (500 mg scale of **1**): Formation of lithium amide base (step 1): 20 min at −20 °C to −30 °C, chiral amine **11** (1.5 equiv), LiBr (2.3 equiv), *n*-BuLi (1.8 M, 1.3 equiv). Lithiation (step 2): 1 h, quinoline **1** (1.0 equiv) at −40 °C or −78 °C. 1,2-Addition (step 3): 1 h 40 min, ketone **2** (1.2 equiv) at −40 °C or −78 °C. Division of solvent volumes for 15, 20, 25, and 30 V: lithium amide base diluted in 5 V, 10 V, 10 V, 10 V, quinoline **1** in 5 V, 5 V, 10 V, 10 V, and ketone **2** in 5 V, 5 V, 5 V, 10 V. HPLC A% obtained after reaction quench with 25% solution of NH_4_Cl (see Supporting Information General Procedure D).

After achieving the developed reaction conditions with high stereoselectivity toward BDQ (**3**), reduction of the amount of solvent was investigated in order to improve further practical applications during reaction scale-up. Different reaction concentrations were therefore studied to understand how concentration would affect the course of the reaction. Initially, the reaction in THF was observed to proceed better when carried out in a more diluted range of 20–30 volumes (V = mL solvent ÷ g of solute) (Figure 3). When exploring the BA reaction in 2-MeTHF at 15, 20, and 25 V, it was observed that 15–20 V of 2-MeTHF produced similar amounts of the *syn*-diastereomer pair, **3** and *ent-***3**, as compared to 30 V of THF, around 70% (HPLC A%).

**Scheme 8:**
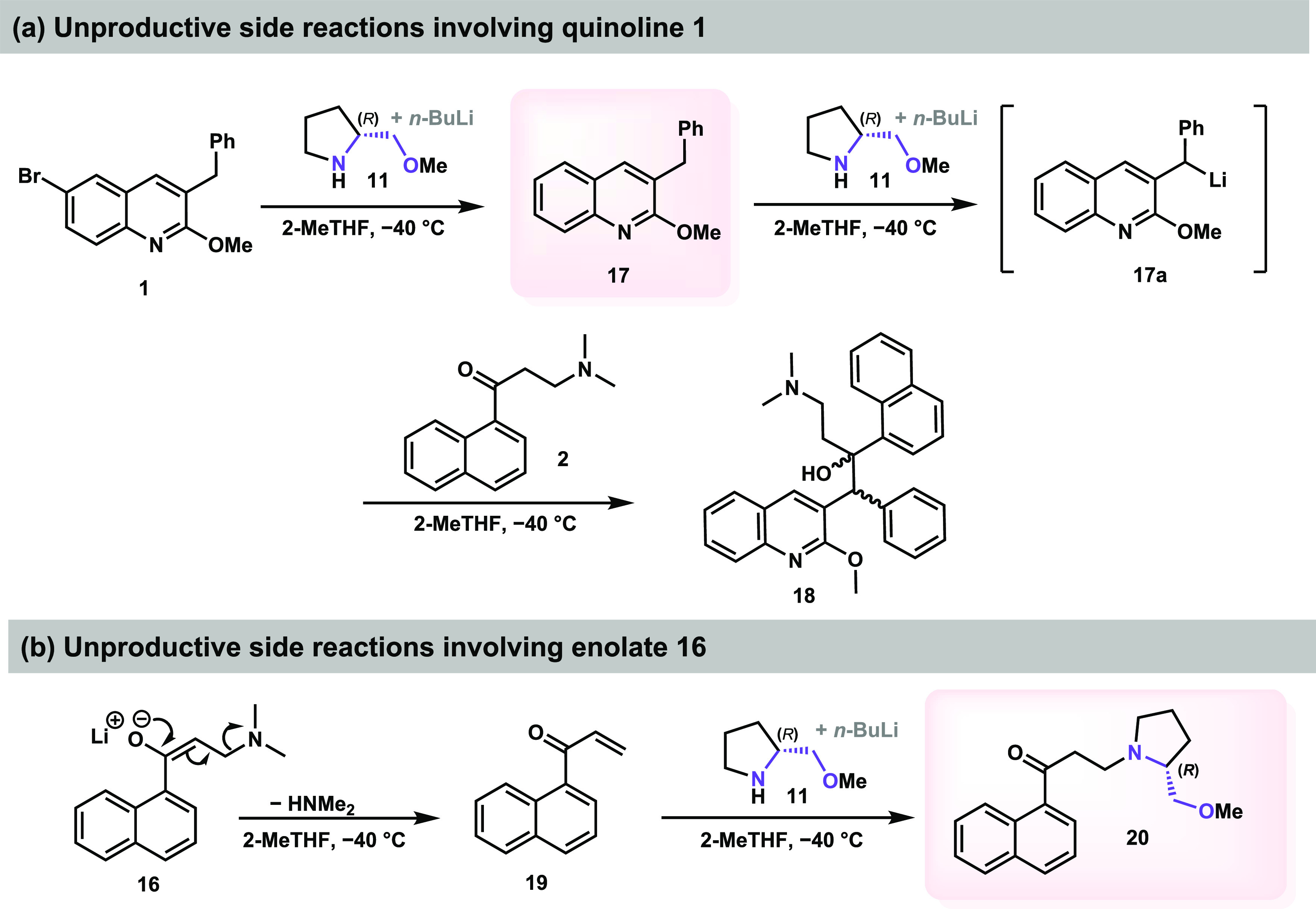
Main Side Reactions Detected in the Developed Asymmetric Methodology for the BA Reaction

Changes in the concentration also impacted the purity profile of this reaction. Additional experiments have shown that when the quinoline **1** solution was further concentrated from 5 to 3 V of THF, a higher amount of the desbromoquinoline **17** was observed due to a competing Li-halogen exchange, indicating that a concentrated medium is not ideal for the lithiation step (Scheme 8) (see Supporting Information Tables S3 and S4). The lithiated compound **17a** can react with **2**, leading to the undesired 1,2-addition product **18** as a mixture of stereoisomers. Higher dilutions tend to slow these side reactions. For instance, when 30 V of THF was used instead of 15 V, only a trace amount of desbromoquinoline **17** was formed, and compound **18** was not observed at all under these conditions. Moreover, the enolization of ketone **2** leads to a facilitated elimination of its dimethylamine moiety, resulting in the side product **19** (Scheme 8). The lithium amide base of **11** can also react in a 1,4-addition with enone **19**, yielding impurity **20**. Based on HPLC analysis, the amount of the side product **20** did not follow a specific trend with the variation in reaction concentration, and it was observed in varying amounts from 1 to 9% (HPLC A%) (see Supporting Information Tables S3 and S5).

In general, the reactions performed in 2-MeTHF also form the aforementioned side products, but when the reactions were run in the 15–20 V range in 2-MeTHF, only trace amounts of side reactions from quinoline **1** were observed (see Supporting Information Table S5). Thus, 2-MeTHF as the reaction solvent in this system offers several clear advantages, namely, (1) the ability to run the process at −40 °C rather than at −78 °C, (2) improved stereoselectivity with the temperature increase, (3) lower reaction volumes, (4) considerably less impurity formation, and (5) 2-MeTHF can be easily dried via azeotropic distillation and provides the possibility of easier solvent recycling, a clear advantage over THF, all of which greatly improve the prospects of manufacturing BDQ (**3**) at a lower overall price point.

### Good Practices to Ensure Reaction Reproducibility

As mentioned before, results obtained from different experiments while using the same reaction conditions can vary to some extent, especially when working on a small scale. There are a few good practices that can be adopted to ensure reproducible results. When all of these requirements are strictly followed, these variations can be considerably minimized. The lithiation/1,2-addition sequence is extremely sensitive to moisture, meaning that all of the components used in this transformation must be freshly distilled and dried. While developing this work, it was found that the azeotropic distillation of the LiBr and quinoline **1** consists of good practice to obtain the lowest water content possible in these materials. Unfortunately, the same procedure cannot be used for ketone **2**, which can easily decompose at the distillation temperatures. Compound **2** is usually commercialized in its more stable hydrochloride salt form and therefore needs to be neutralized prior to performing the BA reaction. Ideally, neutralized ketone **2** should not be stored for long periods, especially at room temperature, as its decomposition toward enone **19** takes place over time. Once neutralized, ketone **2** must be dried at room temperature for a few hours under a vacuum and inert atmosphere; these operations will ensure low levels of impurities.

**Figure 4:**
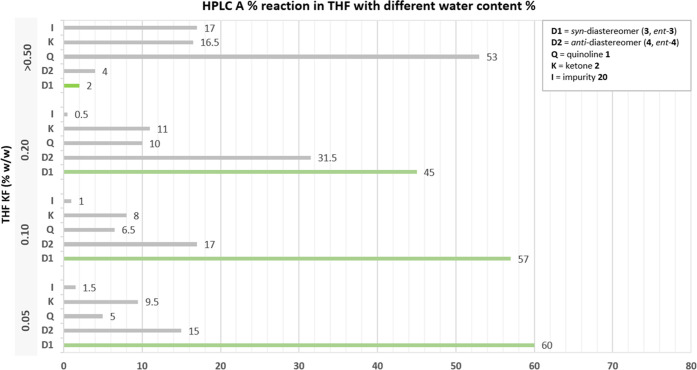
Variation of the THF water content percentage and its effect on the reaction outcome. Reaction conditions (1.0 g scale of **1**, THF, – 78 °C): Formation of lithium amide base (step 1): 20 min at −20 to −30 °C, chiral amine **11** (1.5 equiv), LiBr (2.3 equiv), *n*-BuLi (1.8 M, 1.3 equiv) in 5 V of solvent. Lithiation (step 2): 1 h, quinoline **1** (1.0 equiv) in 5 V. 1,2-Addition (step 3): 1 h 40 min, ketone **2** (1.2 equiv) in 5 V. HPLC A% obtained after reaction quench with 25% solution of NH_4_Cl (see Supporting Information General Procedure D).

A study showing the influence of different percentages of moisture content (% w/w) in THF, determined by the Karl Fischer (KF) titration, was conducted. For the sake of comparison, all experiments were performed at the same scale using properly dried reagents obtained from the same batch, with the goal of minimizing any adverse result caused by the different reagents’ quality. As expected, the increase in water content in the solvent worsened the conversion of the starting materials toward the product. The solvent containing 0.05–0.1% w/w of water provided similar results and good conversion of starting materials based on the HPLC A% analysis; d.r. for both cases was ∼4:1, and the *syn*-diastereomer pair, **3** and *ent-***3**, area was ∼60% (Figure 4). When the water content was 0.2% w/w, the conversion of **1** and **2** decreased, and the dr was reduced to 1.4:1. In addition to the partial quench of lithiated species **1a** and of the lithium amide base obtained from **11**, the presence of water in the system is likely to have a role in perturbing the formation of the lithium aggregates, which ultimately results in the noticeable d.r. variations. A solvent water content of >0.5% w/w shuts down the desired reaction, and only 2% of the *syn*-diastereomer pair was detected. Quinoline **1** corresponded to the major component in the mixture (53%), and the 1,4-addition side product **20** was formed to a larger extent (17%) since the neutralized amine **11** can catalyze the formation of enone **19** through ketone **2** enolization and then act as a nucleophile in the 1,4-addition.

The quality of (*R*)-2-(methoxymethyl)pyrrolidine **11** is also critical. This chiral amine is commercially available from numerous vendors. Reproducible results could not be achieved with different sources of the amine. When comparing good batches of the chiral amine **11** to others that offered an inferior outcome during the BA reaction, no major differences were detected in the nuclear magnetic resonance (NMR) and headspace gas chromatography (GCHS) purity profiles. The chiral purities were also assessed and were >99.5% for both cases. The presence of undetected inorganic salts in the purchased amine **11** was likely to be the main cause of this unexpected behavior.

In order to have better control of the quality of the chiral amine **11** used in this work, we decided to synthesize this material in-house. Synthesis of **11** is described in the literature, making use of diverse approaches with d/l-proline as the starting point.^22^ Distillation of the chiral amine **11** prior to its use in the BA reaction was adopted as the standard procedure. This way, not only the moisture content could be reduced but also the removal of inorganic salts in the crude material. Additionally, storing the pure fraction of the distilled amine **11** over molecular sieves and an inert atmosphere is recommended.

To test the effectivity of the chiral amine **11** purification approach via distillation, d-prolinol was acquired from three different vendors (A, B, and C) and used for the in-house synthesis of (*R*)-2-(methoxymethyl)pyrrolidine (**11**). The goal was to confirm if reproducible results could be obtained independent of where the starting material was coming from, as long as the described purification was being carried out. The distilled chiral amine **11** obtained from d-prolinol purchased from vendors A and C presented very similar analysis results in terms of purity (Table 2). The purity by GCHS and chiral purity determined by supercritical fluid chromatography (SFC) was >99.5% in both cases. Although high chiral purity was observed for amine **11** coming from vendor B (>99.5%), an unknown impurity along with the desired product was detected by GCHS, and the chiral amine **11** presented inferior purity *(∼* 90%).

**Table 2:** Comparison of Important Chiral Amine **11** Parameters after Distillation

entry	vendor	SOR^a^ (deg)	purity by GCHS^b^ (A%)	chiral purity (A%)	KF^c^ (% w/w)
1	A	–8.806	99.9	99.5	∼0.08
2	B	–8.292	89.5	99.9	∼0.10
3	C	–8.674	99.5	100	∼0.12

a SOR: specific optical rotation: solvent CHCl_3_, concentration ∼1.0 g/100 cm^3^.

b GCHS: headspace gas chromatography.

c Karl Fischer (KF) analysis after drying amine **11** over molecular sieves.

The three different batches of distilled amine **11** were used for the BA reaction, which was carried out on a larger scale (5.0 g) in order to minimize the negative influence of moisture content in the experiment outcome (entries 1–3, Table 3). As a result, the percentage of the *syn*-diastereomer pair, **3** and *ent-***3**, was very similar for the three batches, ranging from 72 to 75% (HPLC A%). On the other hand, the amount of the *anti*-diastereomer pair, **4** and *ent-***4**, had a wider variation range from 2 to 13%, resulting in a more noticeable dr difference among these three experiments. The chiral amine **11** obtained from vendors A and C had the same purity profile (Table 2), yet very distinct dr values, ∼19:1 and 6:1, respectively (entries 1 and 3, Table 3). The BDQ (**3**) assay yield varied from 50 to 60%, and surprisingly, the highest yield was associated with the reaction that provided the lowest d.r. (6:1), underlining the importance of not analyzing the reaction’s overall yield, d.r., and ee values separately (entry 3, Table 3). These results suggest that the variation in the d.r. and assay yield of **3** was not linked to the chiral amine **11** purity but was likely due to the unintended introduction of moisture content into the reaction flask during the handling of reagents. With regard to the reaction enantioselectivity, a very small variation was detected in the ee values, which varied from 45 to 50%, showing that the enantioinduction is not as affected by the moisture content present in the reaction as the diastereoselectivity. Nevertheless, the chiral amine **11** obtained from vendor B provided the lowest ee value (45% ee) (entry 2, Table 3), and interestingly, this batch of **11** was the one possessing the lowest purity profile (∼90% vs >99.5% for vendors A and C, Table 2).

**Table 3:** HPLC A% of the Main Components in the Crude Mixture after the Reaction Quench and Assay Yield of *syn*-Diastereomer Pair (**3**, *ent*-**3**) and BDQ (**3**)^a^

			HPLC A%									
entry	d-prolinol vendor	scale of **1** (g)	*syn* (**3** + *ent*-**3**) (%)	*anti* (**4** + *ent*-**4**) (%)	Q (**1**) (%)	K (**2**) (%)	I (**20**) (%)	d.r. (*syn*/*anti*)	e.r. (**3**:*ent*-**3**)^b^	ee (%)	*syn* (**3** + *ent*-**3**) assay yield (%)	BDQ (**3**) assay yield (%)^c^
1	A	5.0	75.0	4.0	5.0	10.0	0.1	18.8:1	2.8:1	48	70	52
2	B	5.0	75.0	2.0	11.4	5.1	0.7	37.5:1	2.6:1	45	69	50
3	C	5.0	72.0	13.0	5.0	1.4	3.2	5.5:1	3:1	50	80	60
4	A	25.0	74.5	5.7	6.5	6.8	3.6	13.1:1	3.1:1	51	85	64
5	C	75.0	77.7	5.7	5.2	7.1	2.9	13.6:1	3.6:1	56	82	64

a Reaction conditions (2-MeTHF, −40 °C): Lithiation: 1 h, quinoline **1** (1.0 equiv, 5 V), and chiral amine **11** (1.5 equiv), LiBr (2.3 equiv), *n*-BuLi (1.8 M, 1.3 equiv) in 10 V of 2-MeTHF. 1,2-Addition: 1 h 45 min, ketone **2** (1.2 equiv, 5 V).

b Enantiomeric ratios (e.r.) were obtained from SFC analysis.

c BDQ (**3**) assay yields based on the purity of crude mass obtained after reaction quench, determined by HPLC wt %.

The higher-purity batches of chiral amine **11** obtained from vendors A and C were selected to be used in the reaction scale-up. As expected, while working on a large scale, the d.r. variations were considerably reduced, resulting in 13.1:1 and 13.6:1 (*syn*/*anti*) for the 25 and 75 g batches, respectively (entries 4 and 5, Table 3). At a 25 and 75 g scale of quinoline **1**, BDQ (**3**) was achieved in a 64% assay yield (entries 4 and 5, Table 3), the highest yield reported for BA reaction to date. This represents a remarkable increase in the BDQ (**3**) yield, more than 50% compared to our previously reported nonasymmetric approach (26–33% assay yield of **3**).^12^

In general, all results indicate that the diastereoselectivity is the most sensitive parameter during BDQ (**3**) synthesis. There is a certain level of complexity associated with the formation of lithium aggregates in solution that makes their precise control very challenging, especially at small scales. Considering the sensitivity of this chemistry, it becomes more evident why a simplified procedure that does not make use of many reagents or additives to promote the desired stereoselectivity is ideal for BDQ (**3**) synthesis. A higher number of reagents and additives introduces additional stoichiometric sensitivities and the potential introduction of perturbing impurities. In this context, the M4ALL’s chiral transfer approach for the BA reaction resembles our previous nonchiral approach since the only methodology modification was the replacement of pyrrolidine with the chiral amine **11**.

## Conclusions

A variety of chiral ligands derived from amino acids containing a N–C–C–O bond structure were employed in the methodology currently used by the manufacturers of BDQ (**3**) fumarate, which consists of quinoline **1** lithiation followed by its 1,2-addition to the ketone **2** fragment. The d-proline derivative, lithium (*R*)-2-(methoxymethyl)pyrrolidide (**11**), was employed as a chiral transfer and found to be sufficiently basic to promote the deprotonation of quinoline **1** while inducing both diastereo- and enantioselectivity toward BDQ (**3**) and maintaining a high conversion rate of starting materials during the BA reaction. The BDQ (**3**) synthesis was shown to be a very sensitive chemical transformation and can be negatively affected by (1) the presence of moisture in the system, (2) lower reagent purity, and (3) an increase in temperature. The lithiation and the 1,2-addition reactions are reversible equilibria, and higher temperatures favor the retro-addition toward starting materials **1** and **2**. The reversibility of the lithiation step allows for the reaction of the lithium amide base of **11** with ketone **2**, favoring the formation of enolate **16**, which constitutes a thermodynamic sink for the desired reaction. Nevertheless, if these three critical parameters are well-controlled, reaction reproducibility can be achieved.

**Scheme 9:**
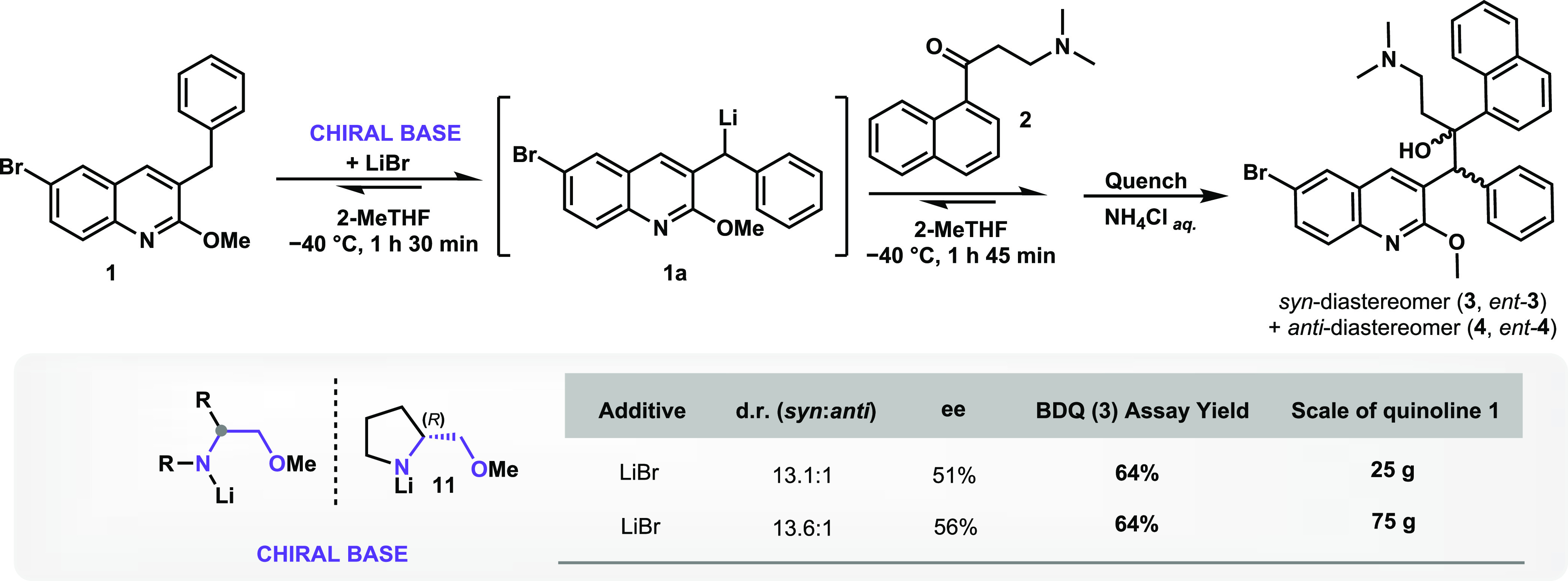
Use of Chiral Lithium Amide of **11** to Promote Enhanced Stereoselectivity toward BDQ (**3**)

An initial reaction optimization showed that switching solvent from THF to 2-MeTHF resulted in slower reaction rates and allowed an increase in the reaction temperature from −78 to −40 °C without favoring the undesired retro-addition. Moreover, solvent volumes can be reduced from 30 to 15–20 V while keeping the same reaction purity profile. Surprisingly, higher temperatures were found to have a significant positive impact on the reaction enantioselectivity toward BDQ (**3**). When using these newly developed conditions and increasing the reaction scale, reduced variation in d.r. was finally achieved, and the combination of high *syn*-diastereomer pair assay yield, high diastereoselectivity, summed to a modest ee (82%, 13.6:1 d.r., 56% ee, respectively, for the 75 g batch experiment), ultimately afforded the highest assay yield for BDQ (**3**) reported to date (64%) (Scheme 9). Further reaction optimization and scale-up can potentially provide even better outcomes. Moreover, the pursuit of other low-cost chiral transfer agents is paramount to achieving improved enantioselectivity. Currently, simplifying the purification process to obtain the enantiopure BDQ (**3**) fumarate salt is being studied. Further improvements in the BDQ (**3**) isolation process are extremely valuable in order to maximize the API final yield and reduce its cost. These findings will be shared in a future publication.

## Supporting Information Available

Experimental procedures, screening tables for chiral ligands and other amines, HRMS of chiral amines **13** and **14**, impurity **20**, and NMR spectra (PDF)
