# Supplementary material for: Application of Chiral Transfer Reagents to Improve Stereoselectivity and Yields in the Synthesis of the Antituberculosis Drug Bedaquiline
Source: Org Process Res Dev. 2023 Oct 13;27(11):2146–59. doi: 10.1021/acs.oprd.3c00287 (PMC10661061; doi:10.1021/acs.oprd.3c00287)
Supplement: Supplementary file 1 — op3c00287_si_001.pdf [file op3c00287_si_001.pdf]

Supporting Information

Application of Chiral Transfer Reagents to Improve Stereoselectivity and Yields in the Synthesis of the Antituberculosis Drug Bedaquiline

Juliana M. S. Robey,[a]* Sanjay Maity,[a] Sarah L. Aleshire,[a] Angshuman Ghosh,[b] Ajay K. Yadaw,[b] Subho Roy,[b] Sarah Jane Mear,[c] Timothy F. Jamison,[c] Gopal Sirasani,[d] Chris H. Senanayake,[d] Rodger W. Stringham,[a] B. Frank Gupton,[a] Kai O. Donsbach,[a] Ryan C. Nelson,[a]* Charles S. Shanahan[a]*

**corresponding authors can be reached at M4ALL@vcu.edu*

[a] Medicines for All Institute, Virginia Commonwealth University, Richmond, VA, 23284-3068, USA

[b] R&D Centre, TCG Life Sciences Pvt. Limited, Kolkata, WB, 700091, India

[c] Department of Chemistry, Massachusetts Institute of Technology, Cambridge, MA, 02139, USA

[d] TCG GreenChem, Inc., Richmond, VA, 23219, USA

Table of Contents

GENERAL INFORMATION S3

Materials and Methods S3

Instrumentation and Analysis S3

LITERATURE OVERVIEW ON BDQ (3) SYNTHESIS AND PURIFICATION S4

Purification via crystallization S4

Literature overview S6

BACKGROUND INFORMATION ABOUT THE REACTION MECHANISM S7

Investigating the lithiation step mechanism S7

*Enolate formation during BDQ (**3**) synthesis S8*

Use of D_2O as the electrophile in the deuterium labeling experiments S10

Additional deuterium labeling experiments based on Le Chatelier's principle S14

Deuterium labeling experiment using pyrrolidine-d_1 .... S18

SYNTHESIS OF KEY RAW MATERIALS 1 AND 2 .... S22

*Synthesis of quinoline **1** fragment S22*

*Synthesis of ketone **2** fragment*......S24

GENERAL METHODS - BDQ (3) SYNTHESIS......S25

Pyrrolidine/chiral ligand system (General Procedure A)......S25
Pyrrolidine/chiral amines system (General Procedure B)......S26
Chiral lithium amides system (General Procedure C)......S27
Optimization experiments using (R)-2-(methoxymethyl)pyrrolidine (General Procedure D)......S28
*Scaling-up BDQ (**3**) synthesis to 75 g (Procedure E)*......S30

GENERAL METHODS – SYNTHESIS OF CHIRAL AMINES AND MAJOR IMPURITY......S32

*Synthesis of (R)-2-(methoxymethyl)pyrrolidine (**11**)*......S32
*Synthesis of the acyclic chiral amines **13** and **14***......S36
*Synthesis of 1,4-Michael addition side product **20***......S40

INITIAL SCREENING OF CHIRAL LIGANDS......S42

Non-amino acid derived chiral ligands – Use of lithium pyrrolidide as the base......S42
Boc-protected amino esters – Use of lithium pyrrolidide as the base......S43
Combination of chiral amino alcohols with lithium pyrrolidide......S43
Combination of acyclic amino acids derivatives as chiral ligands with lithium pyrrolidide......S44
Screening of chiral lithium amides similar to pyrrolidine, N-methylpiperazine, and morpholine......S45

BDQ (3) SYNTHESIS OPTIMIZATION USING LITHIUM (*R*)-2-(METHOXYMETHYL)PYRROLIDIDE (11)......S47

Effect of concentration on the reaction outcome......S47
*Assessing the quality of (R)-2-(methoxymethyl)pyrrolidine (**11**)*......S48

HIGH-PERFORMANCE LIQUID CHROMATOGRAPHY (HPLC) METHODS......S49

Analysis and reaction monitoring......S49
*Analysis of BDQ (**3**) and stereoisomers via Supercritical Fluid Chromatography (SFC)*......S52

^{1}H AND ^{13}C NUCLEAR MAGNETIC RESONANCE (NMR) SPECTRA......S55

REFERENCES......S65

GENERAL INFORMATION

Materials and Methods

Chemicals were obtained from commercial suppliers and were used without any further purification unless otherwise noted. Organometallic reagents were titrated according to a literature procedure before first use and at least weekly thereafter.[1] Dry ice/acetone and dry ice/acetonitrile mixtures were used to achieve −78 ºC and −40 ºC baths, respectively. For small-scale experiments, LiBr (anhydrous, various sources) was dried in a vacuum oven overnight at 100 °C and stored in a desiccator or in a glove box filled with N_2 before use. In the case of reactions performed at scales >5.0 g, azeotropic distillation was applied. Anhydrous THF, 2-MeTHF, or any other dry solvent used in this work were taken from sealed bottles from Sigma-Aldrich (Sure/Seal™). For column chromatography, solvents were purchased in technical grade. Deuterated solvents were purchased from Sigma-Aldrich. All air or moisture-sensitive reactions were performed under an inert atmosphere (Ar or N_2) in glassware that was dried using standard Schlenk techniques. Chromatographic purification was performed using flash column chromatography of the indicated solvent system on silica gel (35 – 70 µm, Acros Organics) unless otherwise noted. Silica plates (TLC Silica 60 F254, Merck, Darmstadt, Germany) were used for thin-layer chromatography. UV active compounds were detected using UV light (λ = 254 nm and λ = 365 nm).

CAUTION: Commercial solutions of reagents such as *n*-butyllithium (*n*-BuLi) and lithium diisopropylamide (LDA) are highly reactive and can be pyrophoric depending on concentrations. Use proper techniques for handling pyrophoric and water-reactive materials and ensure all reagents are fully quenched before work-up. In addition, chemical structures described herein may display bioactive properties. Handle with care.

Instrumentation and Analysis

All NMR spectra were recorded on a Bruker Avance-III (^{1}H NMR: 600 MHz, ^{13}CNMR: 150 MHz) and Bruker Avance-II (^{1}H NMR: 400 MHz, ^{13}C NMR: 100 MHz). Chemical shifts are referenced to residual solvent signals (chloroform-d_1: 7.26 ppm and 77.2 ppm, dimethylsulfoxide-d_6: 2.50 ppm and 39.5, and acetone-d_6: 2.05 ppm and 29.8 for ^{1}H NMR and ^{13}C NMR, respectively) and reported in parts per million (ppm) relative to tetramethylsilane (^{1}H, ^{13}C). Electron spray ionization (ESI) mass spectra were recorded on a 1200-series HPLC-system or a 1260-series Infinity II HPLC-system (Agilent) with binary pump and integrated diode array detector coupled to an LC/MSD-Trap-XTC-mass spectrometer (Agilent) or an LC/MSD Infinitylab LC/MSD (G6125B LC/MSD). High-resolution mass spectrometry (HRMS) was

performed using a JEOL JMS T100LC Accu-TOF mass spectrometer controlled by Mass Center software version 1.3.4 m (JEOL Inc., Tokyo, Japan). Analytical HPLC was performed using an Agilent 1260 Infinity system with a binary pump and a diode array detector. The columns Eclipse Plus C18 (4.6 x 100 mm; 3.5 µM particles) and ChiralPak IC-3, (4.6 mm ID X 250 mm L, 3 µM) were used for analyzing the crude mixture and for SFC analysis, respectively.

LITERATURE OVERVIEW ON BDQ (3) SYNTHESIS AND PURIFICATION

Purification via crystallization

Once the lithiation/1,2-addition sequence of reactions towards BDQ (**3**) is completed, four additional steps are required to obtain the actual API: the enantiopure BDQ (**3**) fumarate salt. After reaction quenching and solvent removal, the obtained residue is submitted for purification. The first step (Step 1, Scheme S1) consists of crystallization in THF to remove the less soluble *anti*-diastereomer pair, compounds **4** and *ent*-**4**. However, a small portion of the *syn*-diastereomer pair, **3** and *ent*-**3**, is lost during this first filtration along with the undesired solid (5 to 10%). The difference in solubility between diastereomers exists, but it is insufficient to precipitate one pair over the other exclusively. Although possible, recovery of **3** and *ent*-**3** from the solid mixture enriched with **4** and *ent*-**4** add extra purification operations to this process.

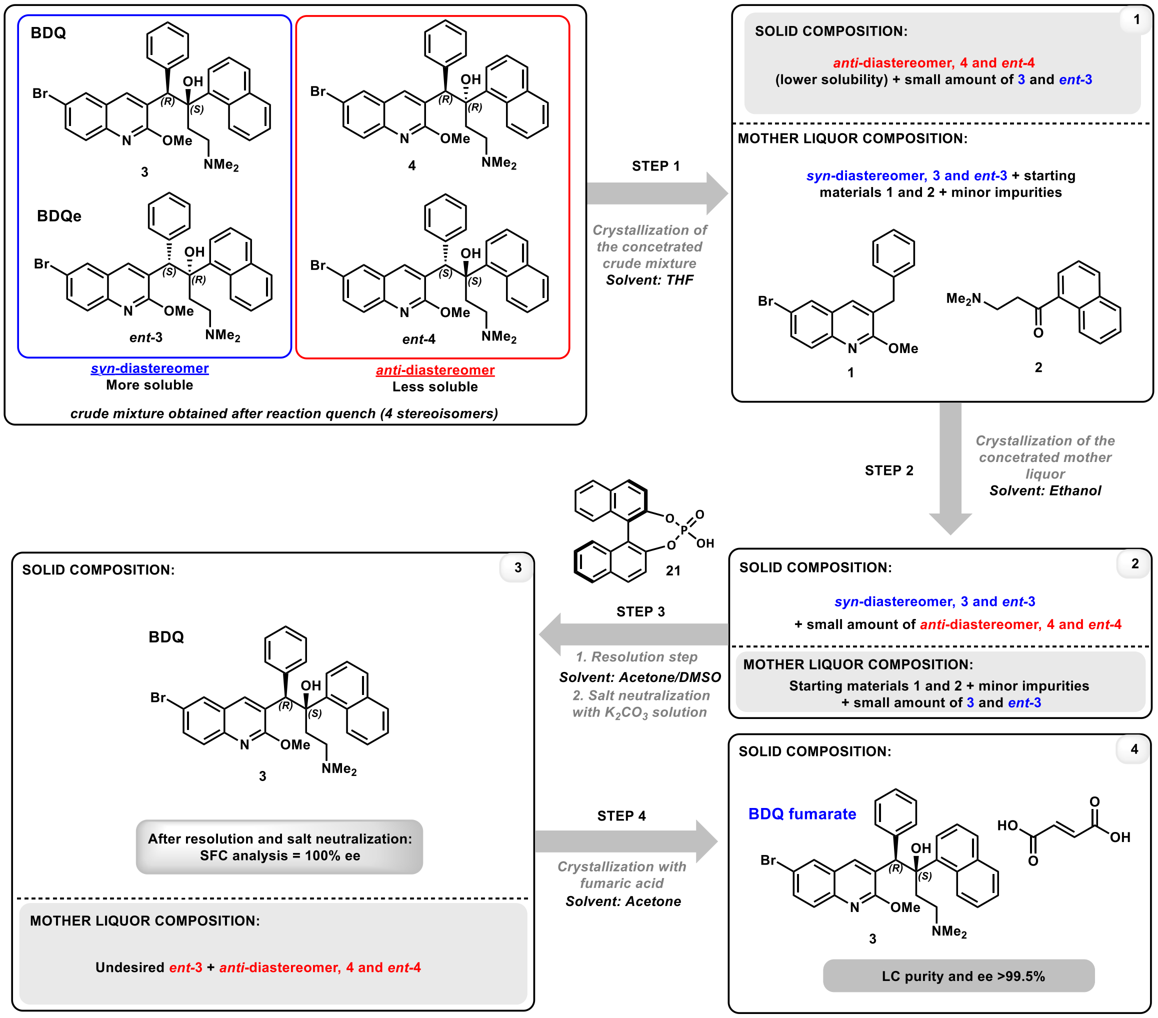

Scheme S1. *Purification steps used to obtain enantiopure BDQ (**3**) fumarate salt*

Not surprisingly, compounds **4** and *ent*-**4** are partially carried over to Step 1 mother liquor containing the major portion of the *syn*-diastereomer pair, **3** and *ent*-**3**. The material obtained after concentration of this mother liquor is submitted to a second crystallization in ethanol (EtOH) (Step 2, Scheme S1). The resulting solid consists of a mixture of the *syn* + *anti* diastereomers pairs, enriched with desired **3** and *ent*-**3** (>90%). Most impurities and remaining starting materials **1** and **2** are almost completely removed in Step 2 mother liquor.

The solid obtained in Step 2 is reacted with the (*R*)-BINOL-phosphoric acid **21**. The remaining *anti*-diastereomer pair, **4** and *ent*-**4**, is entirely purged during this resolution step (Step 3, Scheme S1). This step is very selective towards BDQ (**3**), and high-purity material can be obtained after the BDQ (**3**) phosphate salt neutralization with a K_2CO_3 solution. The typical HPLC A% purity profile and enantiomeric excess

obtained for this step are >99.5%. Finally, the last step (Step 4, Scheme S1) consists of a simple treatment of the enantiopure BDQ (**3**) with fumaric acid to yield the desired fumarate salt of **3**.

Literature overview

The table below shows an overview of the most relevant literature on BDQ (**3**) synthesis (Table S1).[2] Most of them are patents, and therefore, detailed information was not disclosed.

Table S1. *Literature overview for BDQ (**3**) synthesis*

Source	Additive	d.r. (*syn*:*anti*)	e.r. (3:*ent*-3) / ee	*syn + anti* (3+*ent*-3+4+*ent*-4)	*syn*-diast. (3+*ent*-3)	BDQ (3) fumarate yield
**Janssen 2006 (P)[2a-b]**	None	1:1	1:1 / 0%	-	**32% (16% of 3)[a]**	**10%**
**Zentiva 2016 (P)[2c]**	None	1:1	1:1 / 0%	-	**34% (17% of 3)[b]**	-
**FIM 2017 (P)[2d]**	**7**	5:1	~12:1 / 84%	-	**18% of 3[c]**	-
**CAMS 2019 (P)[2e]**	None	1:1	1:1 / 0%	-	**15% (7.5% of 3)[d]**	-
**Mylan 2020 (P)[2f]**	None	1:1	1:1 / 0%	**56%**	**26% (13% of 3)[e]**	-
**Dong-A ST 2020 (P)[2g]**	TMEDA*	1:1	1:1 / 0%	-	**35% (17.5% of 3) [f]**	**9%**
**Naicker et al. 2020 (A)[2h]**	**9**/LiCl	9:1[g]	1:1 / 0%	-	-	-
**M4ALL 2022 (A)[2i]**	LiBr**	2.1:1	1:1 / 0%	**78 - 97% (26 - 32.5 of 3)[h]**	-	-
**Zhang et al. 2022 (A)[2j]**	**8**/LiCl/HMPA	16:1	1:0 / >99%	**22% of 3[i]**	-	-
	**8**/LiCl***	1.4:1	21:1 / 91%	**83% (45% of 3)[i]**	-	-

Lithium diethylamide; ** Lithium pyrrolidide; *** Lithium N-Methylpiperazide as base; P: Patent literature; A: Academic literature

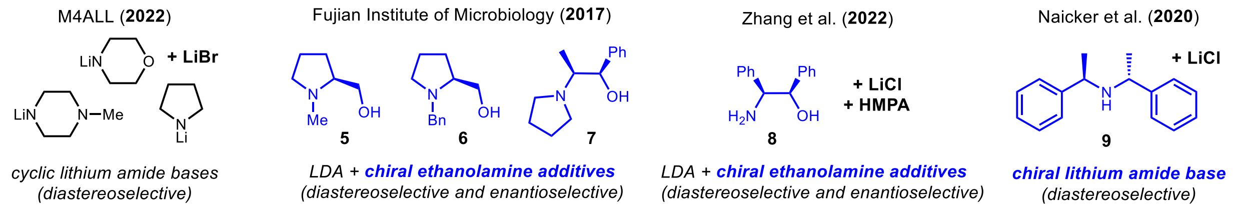

Important notes about Table S1:

[a] A 39% isolated yield was obtained after the second crystallization (steps 1 and 2, Scheme S1) (~11:1 d.r. solid); 32% corresponds to the *syn*-diastereomer pair, **3** and *ent*-**3**. The resolution and neutralization (step 3, Scheme S1) overall yield was 39%. The fumarate salt formation yield was 82% (step 4, Scheme S1). This corresponds to a 10% yield of the enantiopure BDQ (**3**) fumarate salt.

[b] A 37% isolated yield was obtained after the second crystallization (steps 1 and 2, Scheme S1) (12:1 d.r. solid); 34% corresponds to the *syn*-diastereomer pair, **3** and *ent*-**3**; Resolution and fumarate salt formation steps (steps 3 and 4, Scheme S1) were not displayed in the patent.

[c] Patent claimed an 18% isolated yield of the enantiopure BDQ (**3**) (>99.5% ee) after a 3-step purification for removal of the undesired stereoisomers (see reference). According to them, a chiral resolution step was not necessary for the removal of the undesired *ent*-**3**. Fumarate salt formation (step 4, Scheme S1) was not displayed in the patent.

[d] A first crystallization to remove the *anti*-diastereomer pair, **4** and *ent*-**4** (step 1, Scheme S1) was performed, then two crystallizations to obtain high-purity *syn*-diastereomer pair, **3** and *ent*-**3** (step 2, Scheme S1), in 15% isolated yield. Resolution and fumarate salt formation (steps 3 and 4, Scheme S1) were not displayed in the patent.

[e] A 29% isolated yield was obtained after the second crystallization (steps 1 and 2, Scheme S1) (~13:1 d.r. solid); 26% corresponds to the *syn*-diastereomer pair, **3** and *ent*-**3**. Resolution and fumarate salt formation (steps 3 and 4, Scheme S1) were not displayed in the patent.

[f] A 37% isolated yield was obtained after two crystallizations in EtOH (~23:1 d.r. solid) (step 1, Scheme S1); Yield of the *syn*-diastereomer, **3** and *ent*-**3**, was 35%; Resolution step yield: 43%; Neutralization step yield: 67%; Fumarate salt formation yield: 86%. This corresponds to a 9% yield of the enantiopure BDQ (**3**) fumarate.

[g] A 33% conversion was determined by LC-MS, yield was not provided.

[h] Assay yield determined by quantitative NMR (qNMR) of the crude reaction mixture obtained after reaction quench. The isolated yield was not provided.

[i] Isolated yield obtained after column chromatography.

BACKGROUND INFORMATION ABOUT THE REACTION MECHANISM

Investigating the lithiation step mechanism

While studying the non-asymmetric synthesis of BDQ (**3**) in our previous work,[2i] we confirmed that the 1,2-addition between **1a** and **2** leading to the lithium alkoxide **10** is a reversible equilibrium (Scheme S2-a). At −78 °C this equilibrium is shifted in the desired direction, favoring **10**; however, any increase in temperature favors the retro-addition towards **1a** and **2**. To further understand this reaction mechanism, the lithiation step was investigated. It was hypothesized that quinoline **1** deprotonation was also a reversible equilibrium. One result supporting this hypothesis was that when quenching the reaction mixture with a

deuterium source, D_2O or TFA-*d*, the percentage of deuterium incorporated into the remaining ketone **2** was very high (~50%). This demonstrated that ketone **2** enolization was taking place (Scheme 2-b).

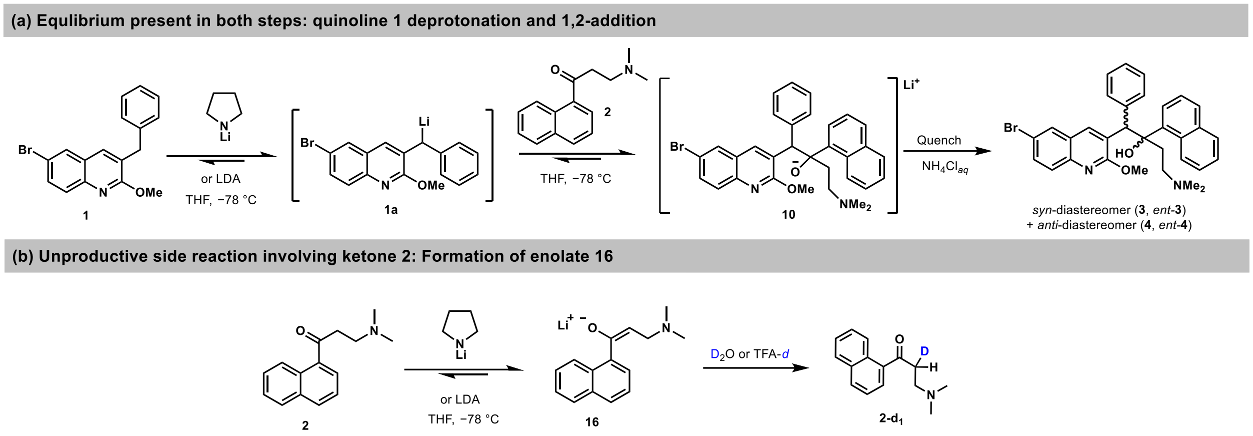

Scheme S2. *(a) Proposal of equilibrium during lithiation and 1,2-addition step; (b) Unproductive reaction involving ketone* ***2***

We considered two main possibilities to explain these observations: (a) lithiation is a reversible equilibrium; when ketone **2** is added to **1a** solution, besides the desired 1,2-addition, the lithium amide base present in the reaction medium due to the lithiation equilibrium can also react with **2** leading to the enolate **16** formation; and/or (b) enolization occurs during the workup, and it is catalyzed by the neutralized amine (DIPA or pyrrolidine).

Enolate formation during BDQ (3) synthesis

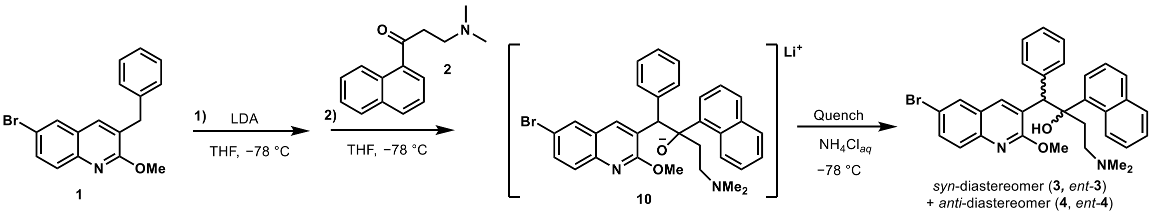

Purpose of the experiment: to analyze the final outcome when the crude reaction mixture is quenched with D_2O or TFA-*d*.

Experimental Procedure: Quinoline **1** (150 mg, 0.46 mmol, 1.0 equiv) was dissolved in anhydrous THF (0.5 mL), and the reaction mixture was cooled to −78 °C. Commercial LDA solution (1 M in THF/hexanes, 0.60 mmol, 0.60 mL, 1.3 equiv) was added dropwise, and the reaction mixture was stirred under Ar atmosphere. After 1 h, a solution of ketone **2** (125 mg, 0.55 mmol, 1.2 equiv) in anhydrous THF (0.5 mL)

was added dropwise to the vial containing the lithiated quinoline. After the addition of ketone **2**, the reaction vial was kept at −78 °C for an additional 1 h. The resulting mixture was quenched with D_2O (2 mL) or TFA-*d* (1 equiv). The organic phase was dried with anhydrous Na_2SO_4 and concentrated under vacuum prior to ^{1}H NMR analysis in $CDCl_3$.

Results: Based on the ^{1}H NMR analysis of the crude mixture after the reaction quench, it was possible to observe the incorporation of ~50% of deuterium into ketone **2** when quenching was performed with D_2O, and 45% with TFA-*d* (Figure S1).

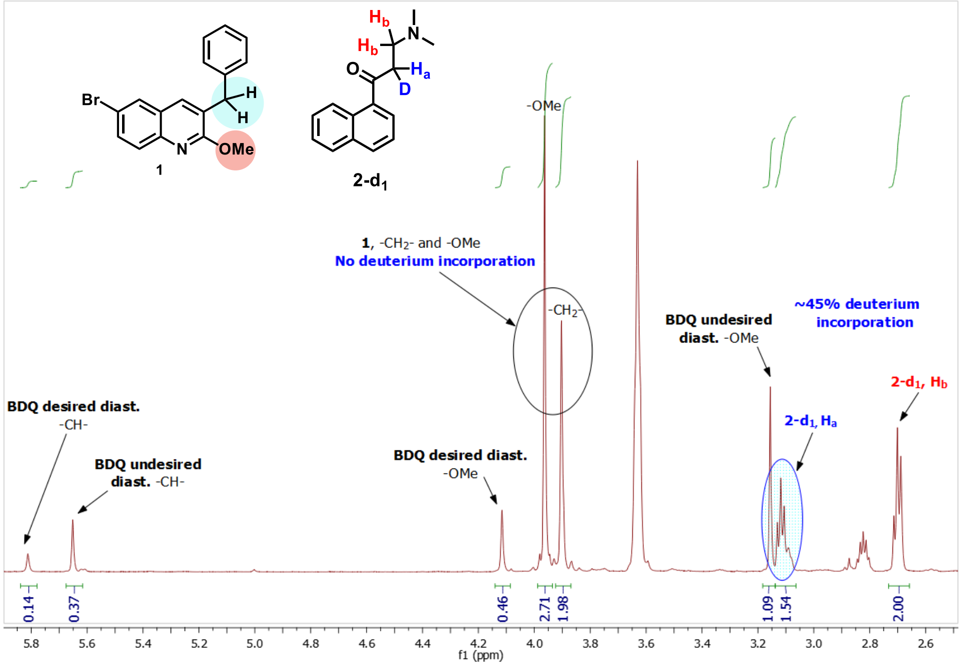

Figure S1. *^{1}H NMR of the crude mixture after quenching with TFA-d at −78 °C*

Control experiment: With these experiments, our goal was to confirm if ketone **2** can be enolized only in the presence of D_2O.

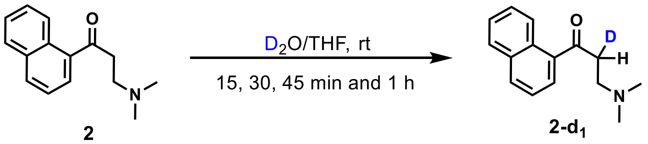

Experimental Procedure: Ketone **2** (125 mg, 0.55 mmol) was solubilized in 2 mL of THF, and 2 mL of D_2O was added to the same vial. The resulting mixture was allowed to stir at room temperature for 15 min to 1 h.

Results: The ^1H NMR ($CDCl_3$) of the reaction mixture was taken at different time points. It seems ketone **2** α-proton is very acidic, and H/D exchange occurs easily. The incorporation of deuterium into **2** increases over time: 15 min (12%), 30 min (24%), 45 min (37%), and 1 h (48%). The quenching process and sample preparation for NMR analysis of the reaction described in Figure S1 take less than 15 min, and yet the analysis showed ~50% of deuterium incorporation. The presence of amine (DIPA or pyrrolidine) in the original reaction system is likely to catalyze the H/D exchange, and this would explain the higher deuterium incorporation in a shorter period of time. Based on these results, we decided to explore different experiments to support our hypothesis of reversible lithiation.

Use of D_2O as the electrophile in the deuterium labeling experiments

Quenching of **1a** with D_2O was assessed at different reaction times to study how the percentage of deuterium incorporation into the quinoline fragment varied over time. In our previous publication, intermediate **1a** assay yield was determined by low-temperature NMR at −78 °C.[2i] When lithium pyrrolidide was used as the base, 91% of **1a** was detected. Although lithiation yield was proven to be high, when **1a** was quenched with D_2O the deuterium incorporation was around 35 to 40%. This result was constant for all time points (1 to 90 min). The mass balance of both deuterated and non-deuterated quinoline recovered by the end of the experiment (**1-d₁** and **1**, respectively) was >95% for all assessed time points, indicating that other side reactions were not taking place (Scheme S3).

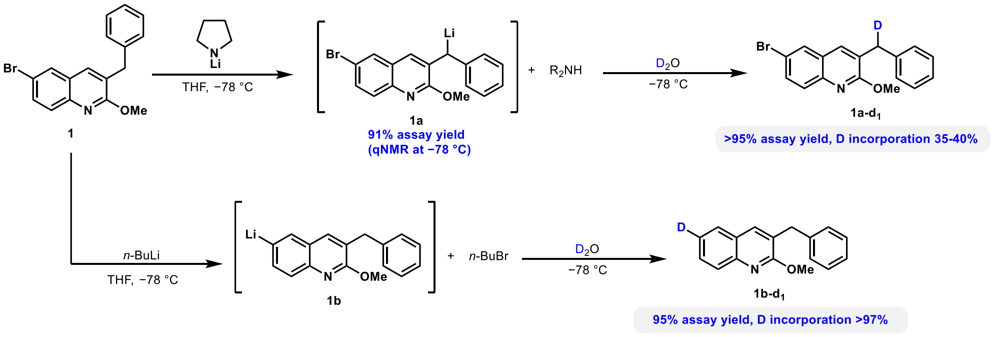

Scheme S3. *Use of D_2O as electrophile during quench of the lithiated intermediates **1a** and **1b***

As a control experiment, the lithiated intermediate **1b** was produced by reacting quinoline **1** directly with *n*-BuLi. In this case, Li/Br exchange was completely favored, and the deuterated desbromoquinoline **1b-d₁** was achieved in 95% (Scheme S3). Interestingly, the deuterium incorporation percentage into the quinoline aromatic position was >97%. Both lithiated species **1a** and **1b** are extremely sensitive to water. Once

formed, they present a very intense purple and reddish color respectively, which fades in seconds when exposed to moisture. Water is known to be more acidic than D_2O, therefore, a better electrophile. Nevertheless, their pK_a difference is small (Δ_{pKa}= 0.44),[3] and assuming **1a** and **1b** are highly reactive, it would not be reasonable to consider that the low incorporation of deuterium into **1a** was due only to decreased reactivity of **1a** with D_2O. At this point, we hypothesized that the lithiation of **1** with lithium pyrrolidide is rapid and reaches equilibrium within the first minutes of the reaction. The addition of D_2O likely disturbs the equilibrium shifting the reaction towards **1**, explaining the lower percentage of deuterium incorporation into **1a**.

Detailed data and procedures:

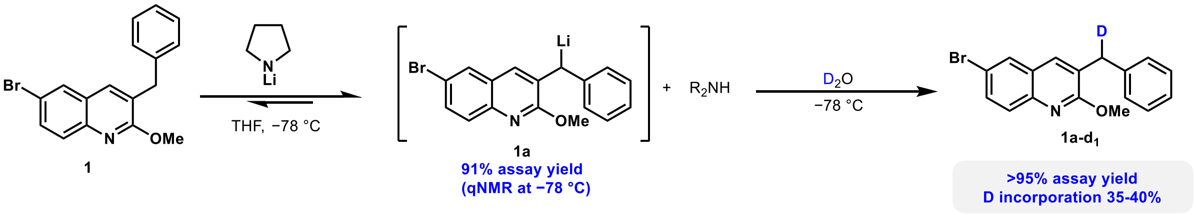

Purpose of the experiment: to analyze how the incorporation of deuterium into lithiated **1a** and mass balance varies over time (duplicate experiment).

Experimental Procedure: Amine (pyrrolidine or *N*-methylpiperazine, 0.91 mmol, 1.5 equiv) was added to an oven-dry vial containing anhydrous THF (2 mL) at 0 °C under N_2 atmosphere, followed by the dropwise addition of *n*-BuLi (2.5 M in THF, 0.79 mmol, 1.3 equiv) over 5 min. The reaction mixture was kept at the same temperature for 20 min, and cooled to −78 °C. Quinoline **1** (200 mg, 0.61 mmol) was dissolved in anhydrous THF (2 mL) and added dropwise to the vial containing the lithium amide base. The reaction mixture was stirred for the indicated time (1−90 min), and D_2O (3 mL) was quickly added to the mixture still at −78 °C (<3 min). Triphenylmethane (0.61 mmol, 1.0 equiv) was added to the sample as the NMR internal standard. Extraction was performed with EtOAc (2 x 5 mL), and the organic phase was separated and dried with anhydrous Na_2SO_4. The solvent was removed under reduced pressure prior to ^1H NMR analysis. Each reaction was performed in duplicate.

Results: When lithium pyrrolidide was used as the base, deuterium incorporation percentage and mass balance were constant from 1 to 90 min. The mass balance of deuterated and non-deuterated quinoline (**1-d$_1$** and **1**, respectively) recovered by the end of the experiment was between 95 and 100% for all time points (Figure S2). Similar results were observed for lithium *N*-methylpiperazide (Figure S3). Each data point in

Figures S2 and S3 corresponds to an individual experiment; duplicate experiments were performed to confirm reproducibility.

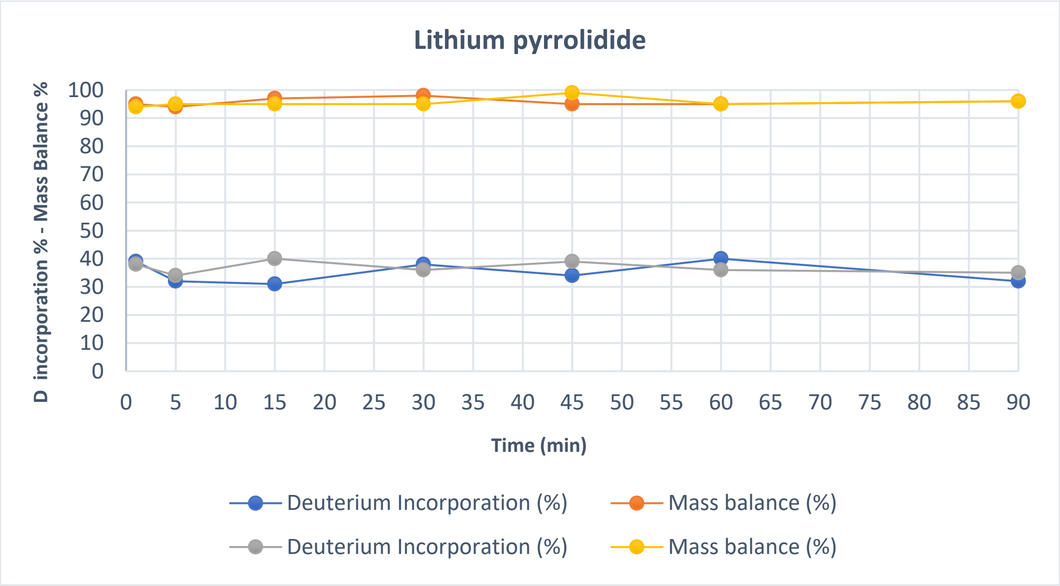

Figure S2. *Variation of deuterium incorporation and mass balance over time when lithium pyrrolidide was used as the base*

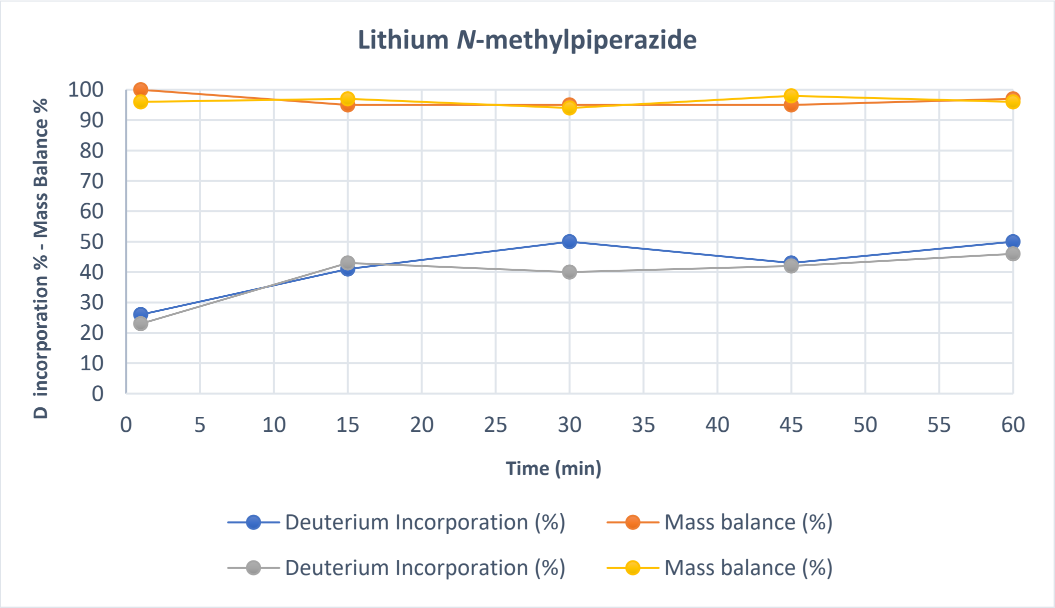

Figure S3. *Variation of deuterium incorporation and mass balance over time when lithium N-methylpiperazide was used as the base*

Control experiment: Li/Br exchange by treating quinoline **1** with *n*-BuLi – generation of lithiated species **1b**. The goal of this experiment was to analyze if a different lithiated intermediate would present similar behavior as **1a** when reacting with D_2O.

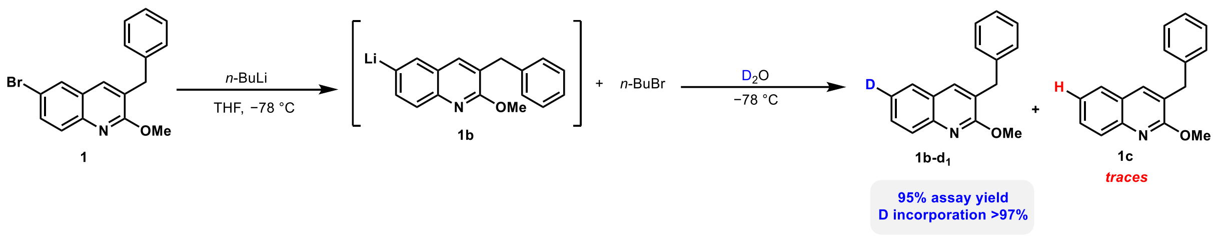

Experimental Procedure: Quinoline **1** (200 mg, 0.61 mmol) was dissolved in anhydrous THF (2 mL) under N_2 atmosphere in an oven-dry vial. The quinoline solution was cooled to −78 °C, and *n*-BuLi (1.3 equiv, 2.5 M solution in THF) was added dropwise over 5 min. The solution presented a reddish color. The reaction was held for 1 h, and quenched by the fast addition (<3 min) of 3 mL D_2O. The resulting mixture was extracted with EtOAc (2 x 5 mL), and the phases were separated. The organic layer was dried with anhydrous Na_2SO_4, and the solvent was removed under reduced pressure. Triphenylmethane (0.61 mmol, 1.0 equiv) was added to the sample as the NMR internal standard. Compound **1b-d$_1$** was obtained in 95% assay yield.

Results: During Li/Br exchange 1-bromobutane (liquid) is formed. This compound possesses a high boiling point (102 °C) and cannot be lost at cryogenic temperature; therefore, reversibility of this transformation is a possibility. When the reaction was quenched with H_2O, the ^{1}H NMR analysis was in accordance with the spectrum of the commercial standard sample of desbromoquinoline **1c**. A new signal (7.37 ppm, triplet, 1H) appeared in the aromatic region. This triplet can barely be noticed when D_2O was used for the quench (Figure S4). These results showed that the forward reaction was completely favored (**1b-d$_1$**, 95% assay yield), and the high percentage of deuterium incorporation into the quinoline aromatic position (>95%) suggests D_2O is a good electrophile in the reaction with the lithiated species **1b**. This reactivity statement should also hold true to lithiated species **1a**.

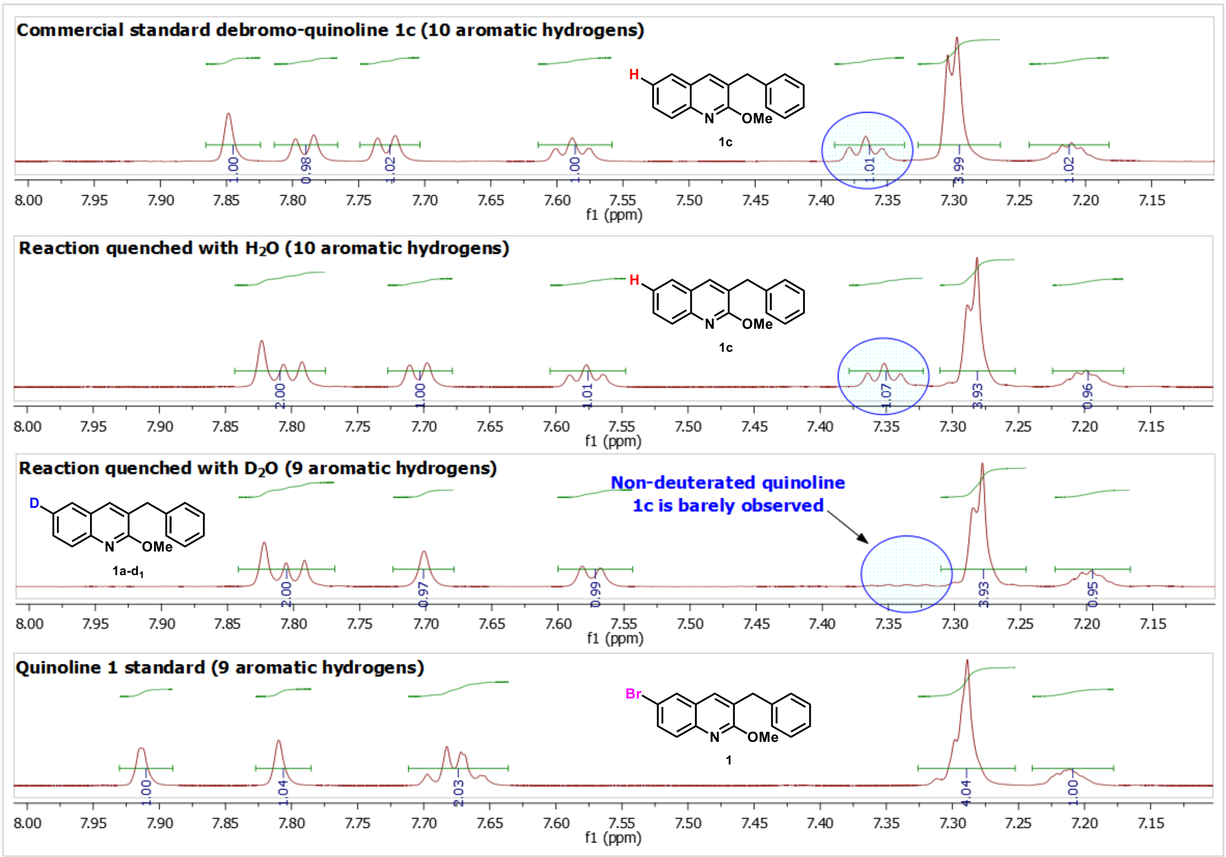

Figure S4. *Comparison of* ^{1}H *NMR of commercial standard of desbromoquinoline* ***1c*** *with the reaction after quenching with* H_2O *or* D_2O *(NMRs of the crude mixture in acetone-*d_6*)*

Additional deuterium labeling experiments based on Le Chatelier's principle

Moving forward with the lithiation equilibrium hypothesis, additional sets of experiments based on Le Chatelier's principle were carried out in order to confirm the reversibility of the lithiation step. We had previously demonstrated that **1a** acts only as a nucleophile by performing the reaction of **1a** with **2-d₂**. (Scheme S4).[2i] The absence of deuterium incorporation into quinoline confirmed that the lithiated intermediate **1a** is not likely to have any participation in the ketone **2** enolization.

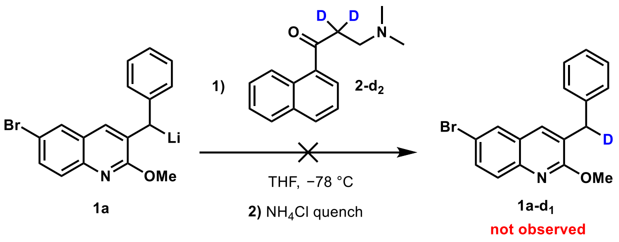

Scheme S4. *Mechanistic investigations of the lithiation step via deuterium labeling experiments*

In the first set of experiments, a solution of **1** and lithium pyrrolidide was treated with increasing amounts of pyrrolidine (1.5, 5.6, and 11.2 equiv). If the lithiation step has some reversible component, additional pyrrolidine, a reaction product, should disfavor deprotonation. Indeed, after quenching these systems with D_2O, the amount of deuterium incorporation into quinoline **1a** decreased from approximately 30% to 10%, then to 5%, respectively.

For a second set of reactions, the temperature dependence of the deprotonation reaction was explored, reasoning that changing the temperature of a reversible reaction could alter the relative ratios of products and reactants. To probe this effect, a solution of **1** and lithium pyrrolidide was prepared at room temperature. After quenching an aliquot of this solution with D_2O, 50% deuterium incorporation into **1a** was observed. After cooling this same reaction mixture to −78 °C followed by D_2O quench at low temperature, only 26% deuterium was incorporated into **1a**, with >95% quinoline **1**+**1a-d$_1$** mass balance, which indicates that other undesired side reactions or decomposition were not taking place.

Detailed data and procedures:

Set 1 – Pyrrolidine concentration: If lithiation is a reversible, increasing the pyrrolidine concentration should shift the equilibrium back to the starting materials. Consequently, deuterium incorporation into **1a** after reaction workup with D_2O must decrease. Three pyrrolidine concentrations were analyzed (1.5, 5.6, and 11.2 equiv relative to quinoline **1**) at −78 °C, and 15 min reaction time (Table S2) (Figure S5).

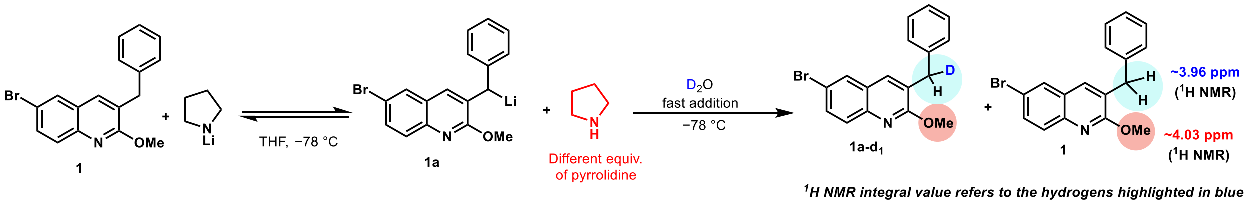

Experimental Procedure: Freshly distilled pyrrolidine (1.5, 5.6, or 11.2 equiv) was added to an oven-dry vial containing anhydrous THF (2 mL) at 0 °C under N_2 atmosphere, followed by the dropwise addition of *n*-BuLi (2.5 M in THF, 0.79 mmol, 1.3 equiv) over 5 min. The reaction mixture was held for 20 min, and cooled to −78 °C. Quinoline **1** (200 mg, 0.61 mmol) was dissolved in anhydrous THF (2 mL) under inert atmosphere, and added dropwise to the vial containing the lithium amide base. The reaction mixture was stirred for 15 min, and D_2O (3 mL) was quickly added to the mixture at −78 °C (<3 min). Triphenylmethane (0.61 mmol, 1.0 equiv) was added to the sample as the NMR internal standard. Extraction was performed with EtOAc (2 x 5 mL), and the organic phase was separated and dried with anhydrous Na_2SO_4. The solvent was removed under reduced pressure prior to ^1H NMR analysis. Each reaction was performed in duplicate.

Table S2. *Variation of the percentage of deuterium incorporation into quinoline **1a** while increasing pyrrolidine concentration*

**Lithium pyrrolidide**				
**Time (min)**	**Pyrrolidine equiv**	**Integral Value (H)**	**Deuterium Incorporation (%)**	**Mass balance (%)**
15	1.5	1.69	31	100
15	5.6	1.83	17	100
15	11.2	1.93	7	100
**Duplicate experiment**				
**Time (min)**	**Pyrrolidine equiv**	**Integral Value (H)**	**Deuterium Incorporation (%)**	**Mass balance (%)**
15	1.5	1.6	40	100
15	5.6	1.83	17	100
15	11.2	1.9	10	100

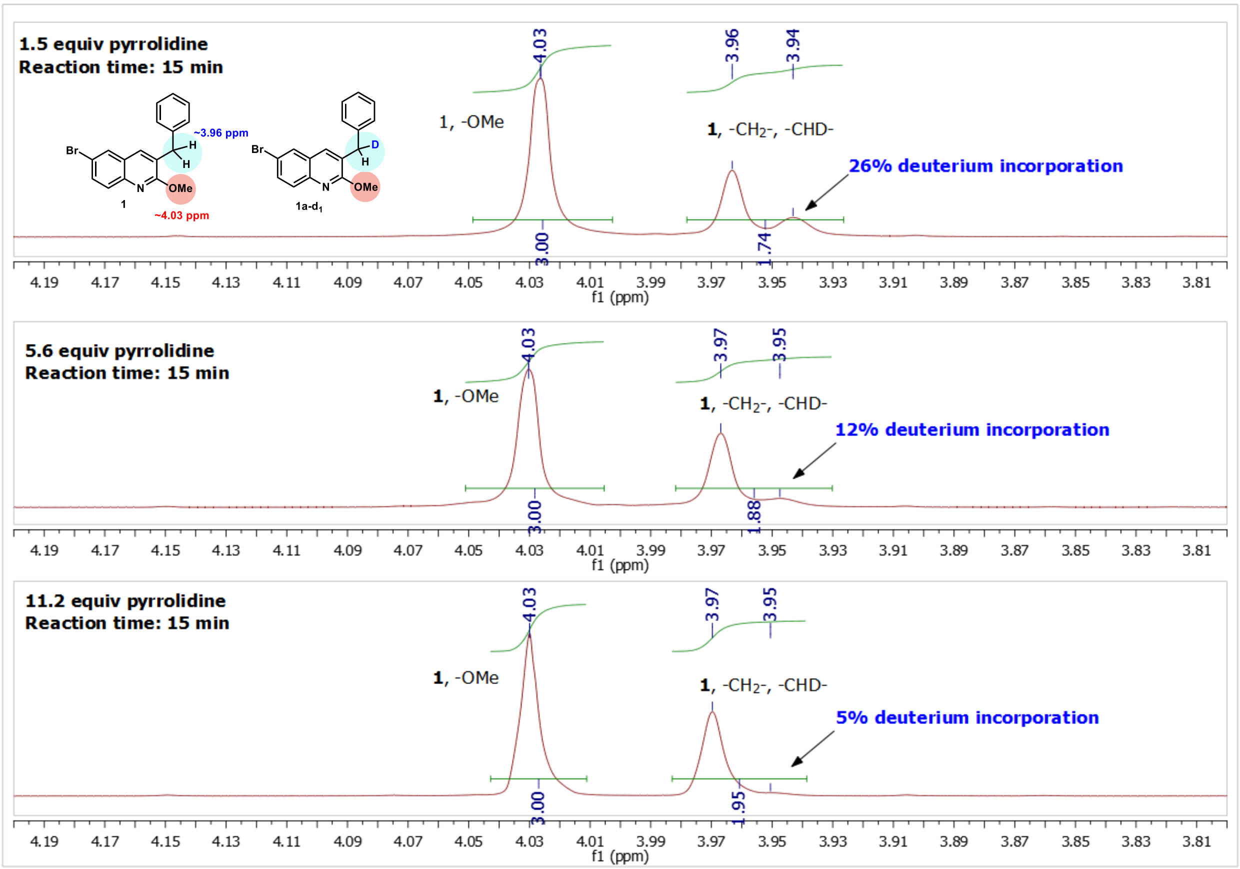

Figure S5. *^{1}H NMR of the crude reaction mixture after quenching with D_2O at different pyrrolidine concentrations*

Set 2 – Temperature effect: If lithiation is a reversible equilibrium, changes in temperature must lead to different **1a** concentrations. We expected that increasing temperature would favor **1a** formation. Therefore, higher deuterium incorporation should be observed when the reaction is quenched with D_2O at room temperature.

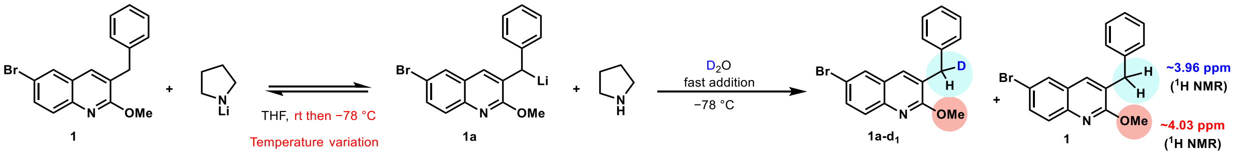

Experimental Procedure: Freshly distilled pyrrolidine (0.91 mmol, 1.5 equiv) was added to an oven-dry vial containing anhydrous THF (2 mL) at 0 °C under N_2 atmosphere, followed by the dropwise addition of *n*-BuLi (2.5 M in THF, 0.79 mmol, 1.3 equiv) over 5 min. The reaction mixture was held for 20 min, and cooled to −78 °C. Quinoline **1** (200 mg, 0.61 mmol) was dissolved in anhydrous THF (2 mL) and added dropwise to the vial containing the lithium amide base over 5 min. The reaction mixture was stirred for 15

min and warmed up to room temperature for 45 min. An aliquot was collected and quenched with D_2O for ^{1}H NMR analysis. The vial was transferred back to the low-temperature bath at −78 °C, where it was kept for an additional 45 min. The quench was performed by quickly (<3 min) adding D_2O (3 mL) to the mixture at −78 °C. Triphenylmethane (0.61 mmol, 1.0 equiv) was added to the sample as the NMR internal standard. Extraction was performed with EtOAc (2 x 5 mL), and the organic phase was separated and dried with anhydrous Na_2SO_4. The solvent was removed under reduced pressure prior to ^{1}H NMR analysis.

Results: ^{1}H NMR analysis of the aliquot collected at room temperature showed around 50% incorporation of deuterium into quinoline **1a**. Whereas deuterium incorporation decreased by half when the quenching of the same mixture was performed at −78 °C (Figure S6). The mass balance of recovered quinoline (mixture of **1** and **1a-d₁**) was >95% after quenching. These results are in accordance with our previously formulated hypothesis.

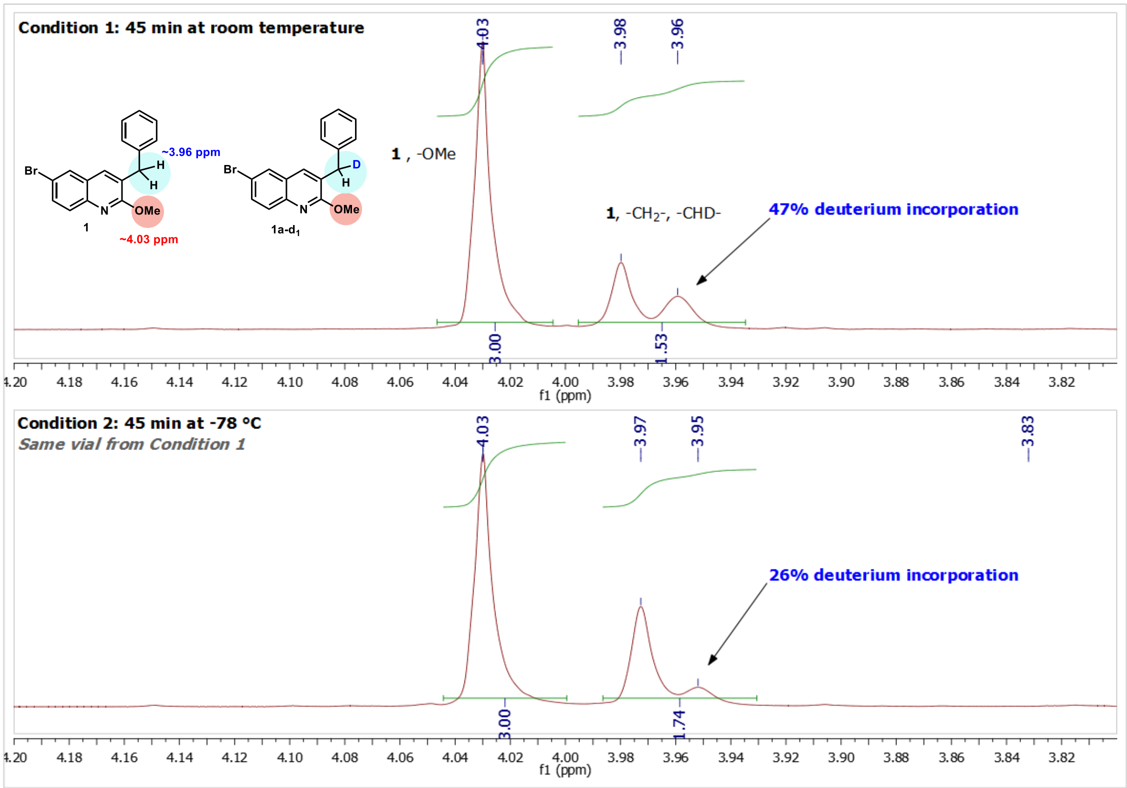

Figure S6. *^{1}H NMR of the crude mixture after reaction quenching with D_2O at different temperatures*

Deuterium labeling experiment using pyrrolidine-d_1

Pyrrolidine-d_1 was added to a solution of **1a** at two different temperatures followed by a quench with H_2O. In both cases, deuterium incorporation into **1a** was observed: 3% at −78 °C and 43% at room temperature. This result points to the deprotonation of pyrrolidine-d_1 by **1a** (Scheme S5).

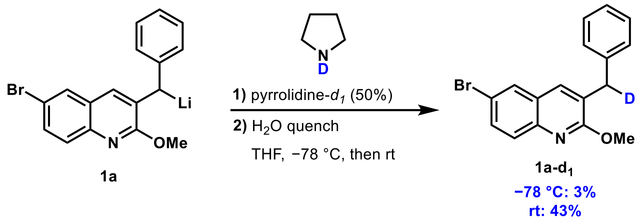

Scheme S5. *Treatment of intermediate* ***1a*** *with pyrrolidine-d$_1$ solution*

Detailed data and procedures:

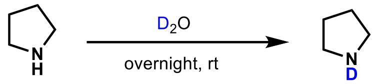

Synthesis of pyrrolidine-*d$_1$*:[4] Pyrrolidine (20 mL) and D_2O (20 mL) were added to a 100 mL round-bottom flask. This miscible mixture was allowed to stir overnight at room temperature. After this period, pyrrolidine (-ND/-NH) was extracted using toluene (5 x 10 mL), and sodium chloride (NaCl) was added to break the emulsion. The organic phase was dried with anhydrous $MgSO_4$, and after filtration, the resulting mixture was stirred with an excess of calcium hydride (CaH_2) for 4 h to remove any remaining D_2O. The pyrrolidine mixture (-ND/-NH) was purified via distillation under atmospheric pressure.

Results: A mixture of deuterated and non-deuterated pyrrolidine (-ND/-NH, *ca* 50% deuterium incorporation) was obtained. During distillation, it was not possible to obtain a pure fraction of this mixture; toluene was present in all of the collected fractions. The sample used for the experiment corresponds to a mixture of pyrrolidine -ND/-NH (50%) in toluene (~1:6 ratio by ^{1}H NMR, pyrrolidine-*d$_1$*:toluene) (Figure S7-b).

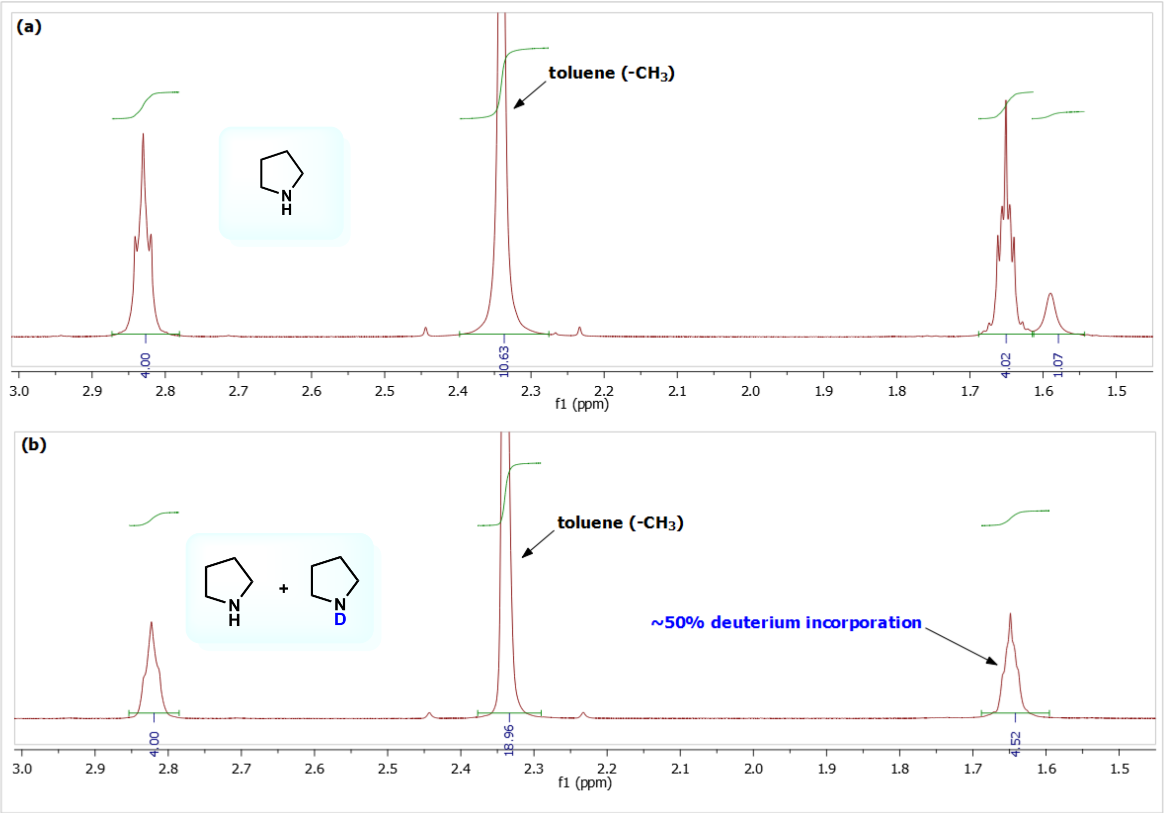

Figure S7. *^{1}H NMR (CDCl3) of (a) pyrrolidine and toluene mixture in (1:3.5), and (b) pyrrolidine NH/ND 50% and toluene mixture (1:6)*

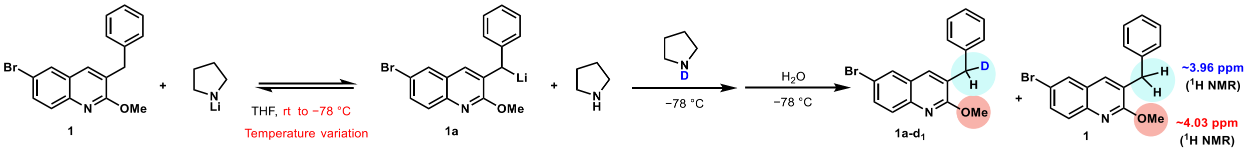

Experimental Procedure: In an oven-dry vial containing dry THF (2 mL) under N_2 atmosphere, freshly distilled pyrrolidine (0.91 mmol, 1.5 equiv) was added, and the solution was cooled down to 0 °C, followed by the dropwise addition of *n*-BuLi (2.45 M in hexane, 0.79 mmol, 1.3 equiv) over 5 min. After 20 min, the resulting mixture was cooled to −78 °C, and a solution of quinoline **1** (200 mg, 0.61 mmol, 1 equiv) in dry THF (2 mL) was added to the vial containing the lithium amide base over 5 min. Two different experiments were carried out: (1) After 5 min reaction time at −78 °C, the addition of 2.4 mL of pyrrolidine-d_1 (50%) mixture in toluene (corresponding to ~0.5 mL of pyrrolidine/pyrrolidine-d_1, ~10 equiv) was performed, and the reaction stirred for 120 min at the same temperature, and (2) After 5 min at −78 °C, the addition of 2.4 mL of pyrrolidine-d_1 (50%) mixture in toluene was performed, and the reaction mixture was warmed up to room temperature and stirred for 90 min. After the desired reaction time, the mixture was

quenched with a saturated aqueous solution of NH_4Cl (3 mL, fast addition). The organic phase was separated and dried with anhydrous Na_2SO_4. The solvent was removed under reduced pressure prior to ^{1}H NMR analysis.

Results: At −78 °C, the H/D exchange occurs very slowly, and only 3% of deuterium was incorporated into **1a**. Quinoline **1a-d$_1$** C-D coupling around 35 ppm can be observed in the ^{13}C NMR (Figure S8-a). Deuterium incorporation was more evident at room temperature, likely due to the faster H/D exchange, around 43%. C-D coupling signal in ^{13}C NMR was considerably more intense as well (Figure S8-b).

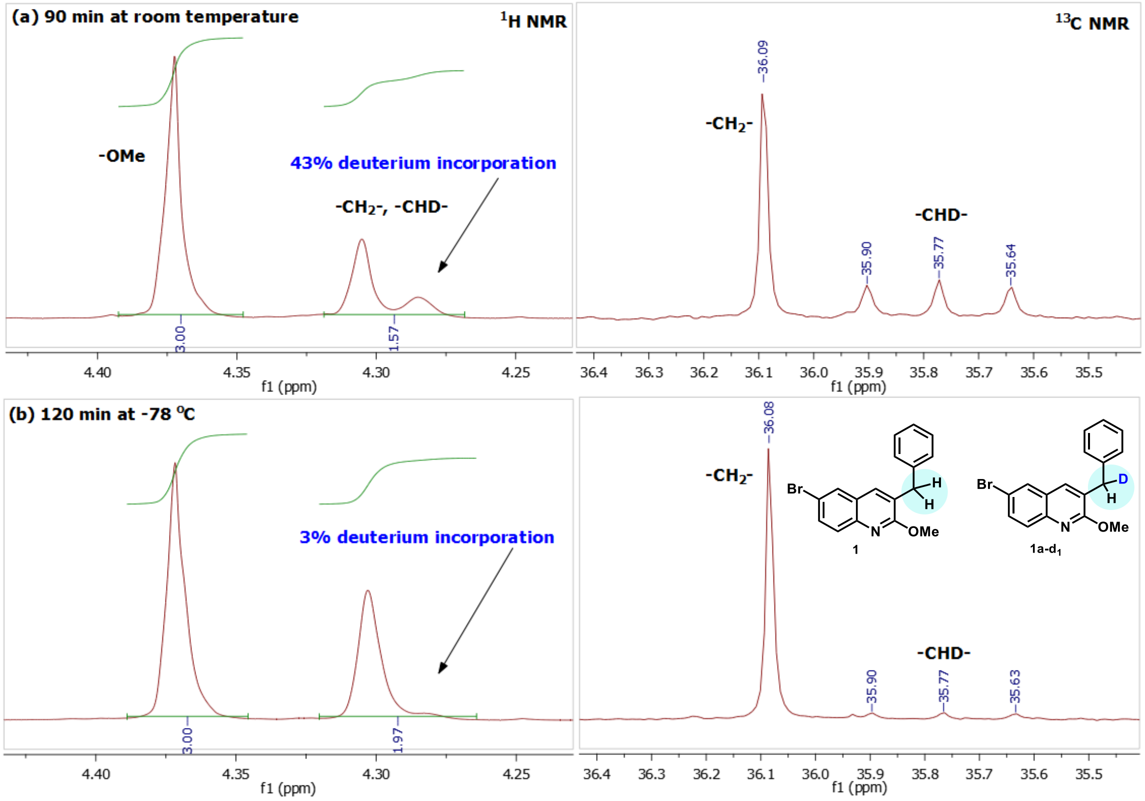

Figure S8. *^{1}H and ^{13}C NMR of the crude mixture after reaction quenching with NH_4Cl aqueous solution at (a) room temperature, and (b) −78 °C*

SYNTHESIS OF KEY RAW MATERIALS 1 AND 2

Synthesis of quinoline 1 fragment

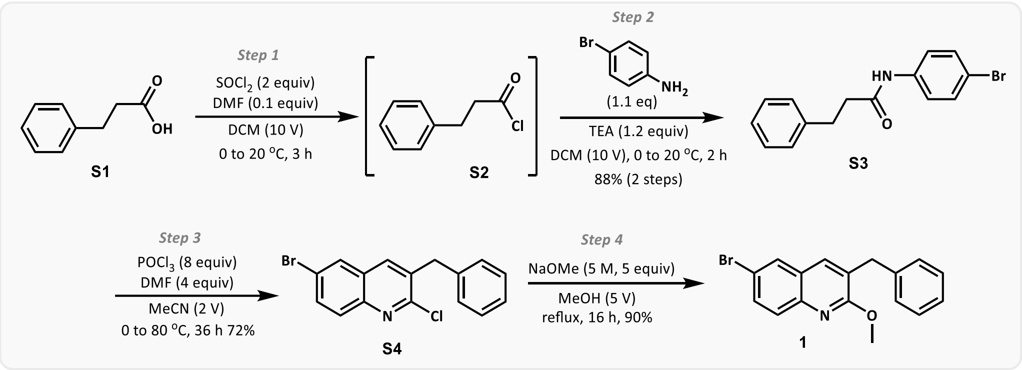

Steps 1 and 2: *Synthesis of the acyl chloride of the 3-phenylpropanoic acid and its reaction with 4-bromoaniline*

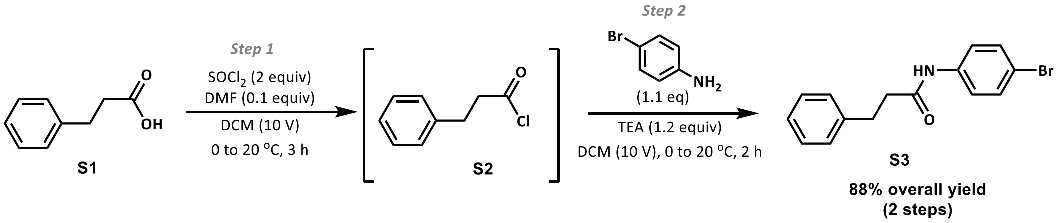

N-(4-bromophenyl)-3-phenylpropanamide (S3). To a solution of 3-phenylpropanoic acid (**S1**) (500 g, 3.32 mol, 1 equiv) and DMF (25.5 mL, 66.6 mmol, 5.1 mL, 0.1 equiv) in DCM (5 L, 10 V), $SOCl_2$ (483 mL, 2 equiv) was added dropwise over 1 h at 0 °C. The reaction mixture was allowed to warm to 20 °C, stirred for 2 h, and concentrated under vacuum to give **S2** as colorless oil (564 g, crude). The obtained acyl chloride **S2** was solubilized in DCM (1.5 L) and added dropwise to a solution of 4-bromoaniline (604.1 g, 1.05 equiv) and triethylamine (516.72 mL, 1.2 equiv) in DCM (5 L, 10 V) over 1 h at 0 °C under N_2 atmosphere. The mixture was warmed to 20-25 °C and stirred for 1 h. After completion of the reaction, solvent was evaporated under reduced pressure at 35-40 °C. Water (1 L, 2 V) was added to the obtained solid and stirred for 6 h at 25-30 °C. The solid was filtered, washed with MTBE (500 mL, 1 V), and dried under vacuum to afford the product **S3** as a white solid in 88% yield (890 g, 99.9% purity by HPLC). NMR data is in accordance with the literature.[2i]

Step 3: *Cyclization toward chloro-quinoline fragment*

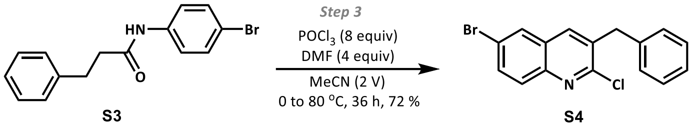

3-benzyl-6-bromo-2-chloroquinoline (S4). DMF (305.4 mL, 4 equiv) was added to a flask containing $POCl_3$ (737.6 mL, 8 equiv) at 0 °C and stirred for 1 h under N_2 atmosphere, followed by the addition of a solution of compound **S3** (300 g, 1 equiv) in MeCN (900 mL, 2 V). The temperature was increased to 80 °C, and reaction mixture was stirred for 36 h at the same temperature. After completion of the reaction, the mixture was cooled to 10-15 °C, and water was slowly added (12 L), and stirred for 30-45 min. After solid precipitation was observed, reaction mixture was further stirred for another 1 h at 25-30 °C. The solid was filtered, and washed with water (2 x 300 mL), followed by cold MeOH (2 x 300 mL). After drying under vacuum, compound **S4** was obtained as an off-white solid in 72% yield (700.0 g, 99.9% purity by HPLC). NMR data is in accordance with the literature.[2i]

Step 4: *Chloride displacement with sodium methoxide (NaOMe)*

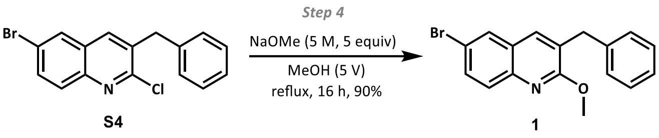

3-benzyl-6-bromo-2-methoxyquinoline (1). To a solution of compound **S4** (700.0 g, 1 equiv) in MeOH (3.5 L, 5 V), NaOMe (5 M in MeOH, 5 equiv) was added and the reaction mixture was stirred for 8 h at 80 °C under N_2 atmosphere. After completion of the reaction, resulting mixture was cooled to 25-30 ºC and concentrated under reduced pressure to remove the MeOH. Water was added (4 V) and stirred for another 6 h at 25-30 °C. The resulting solid was filtered, washed with water (1 V) and cold MeOH (1 V), and finally dried under vacuum at 40-45 °C for 6 h. Quinoline **1** fragment was obtained as an off-white solid in 90% yield (620.0 g, 98% purity by HPLC). NMR data is in accordance with the literature.[2i]

Note: To ensure low water content, quinoline **1** was dried via azeotropic distillation (THF or 2-MeTHF) prior to its use in the bedaquiline assembly (BA) reaction.

Synthesis of ketone 2 fragment

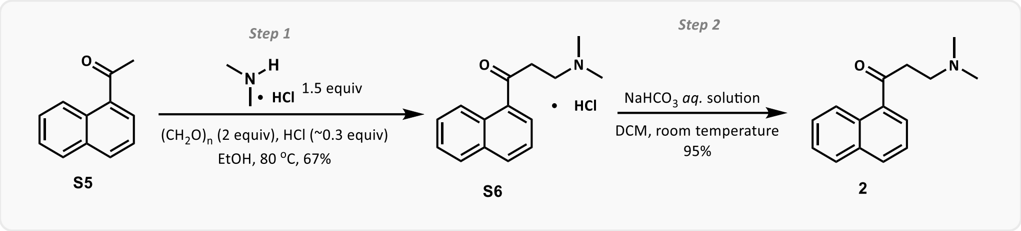

Step 1: *Reaction of 1-acetylnaphthalene with iminium ion*

3-(dimethylamino)-1-(naphthalen-1-yl)propan-1-one hydrochloride salt (S6). To a suspension of *N*,*N*-dimethylamine hydrochloride (179.5 g, 1.5 equiv) and paraformaldehyde (93.8 g, 2 equiv) in EtOH (438 mL, 1.75 V) at room temperature, 1-acetylnaphthalene (**S5**) (250.0 g, 1 equiv) was added followed by the dropwise addition of concentrated HCl (12 M, 31.4 mL, ~0.3 equiv). The reaction mixture was warmed to 80 °C and stirred for 30 h at the same temperature. After completion, the resulting mixture was cooled down and concentrated under reduced pressure to remove the EtOH at 50-55 °C. MeCN (4 V) was added to the obtained crude mass (476.0 g) and stirred for 5 h at 25-30 °C. The resulting solid was filtered, washed with MeCN (1 V), and dried under vacuum at 40-45 °C for 5 h to afford compound **S6** as an off-white solid in 67% yield (227.0 g, 99% purity by HPLC). NMR data is in accordance with the literature.[5]

Step 2: *Hydrochloride salt neutralization*

3-(dimethylamino)-1-(naphthalen-1-yl)propan-1-one (2). Hydrochloride salt (**S6**) (80.0 g, 1 equiv, 98% purity by HPLC) and water (800 mL, 10 V) were charged into a 5 L 3-neck round-bottom flask at 25-30 °C. The resulting mixture was stirred until full solubilization of the salt was achieved (10-15 min). DCM (800 mL, 10 V) was added, and the mixture was stirred for 15-20 min. A saturated aqueous solution of $NaHCO_3$ (800 mL, 10 V) was slowly added over 45 min via an addition funnel (rate of addition= 17.7 mL/min), and stirring was held for an additional 8-10 min. The biphasic mixture was transferred to a separatory funnel and the layers separated. The aqueous phase was extracted with DCM (2 x 400 mL, 10 V). Organic layers were combined and dried with anhydrous $MgSO_4$, filtered through a Büchner funnel, and the $MgSO_4$ bed was washed with DCM (80 mL, 1 V). Solvent was removed under vacuum at 30-35 °C for 1-2 h (740-750 mmHg) to afford compound **2** as a pale-yellow liquid in 95% yield (66.0 g, 95% purity by HPLC). NMR data is in accordance with the literature.[5]

Note: Prior to its use in the BA reaction, compound **2** should be solubilized in anhydrous solvent (THF or 2-MeTHF) under inert atmosphere (N_2), followed by the addition of activated molecular sieves added (4 Å) to ensure low water content. Drying ketone **2** at high temperatures is not recommended (decomposition, undesired side reactions). Avoid storing ketone **2** for long periods since its decomposition can take place over time. Ideally, hydrochloride salt **S6** should be neutralized only prior to compound **2** use in the BA reaction. If storage of **2** cannot be avoided for any reason, opt for storing this material at low temperature and under inert atmosphere.

GENERAL METHODS – BDQ (3) SYNTHESIS

Pyrrolidine/chiral ligand system (General Procedure A)

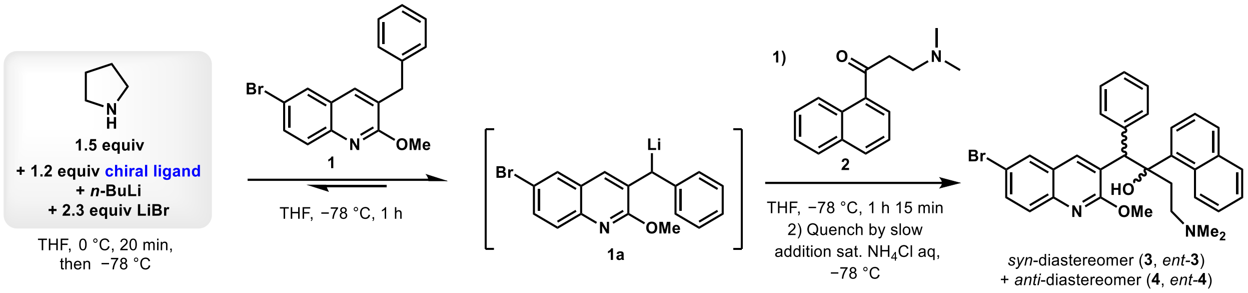

In a glove box filled with N_2, a 40 mL oven-dry vial containing a stir bar was charged with anhydrous LiBr (120 mg, 1.38 mmol, 2.3 equiv). The vial was closed with a TFE-lined silicone septa cap and removed from the glove box, and a balloon containing N_2 was attached. Dry THF (2 mL) was added to this vial, and the resulting mixture was stirred at room temperature until full solubilization of the salt was achieved. Pyrrolidine (75 µL, 0.9 mmol, 1.5 equiv) and the chiral ligand (0.72 mmol, 1.2 equiv) were added. The mixture was cooled down to 0 °C followed by the dropwise addition of *n*-BuLi (2.5 M in hexane, the equivalents amount varies depending on the ligand, see notes below). The mixture was stirred for 20 min (light-yellow solution). The reaction was cooled to −78 °C, and a solution of quinoline **1** (200 mg, 0.60 mmol, 1 equiv) in dry THF (2 mL) was added dropwise into the reaction mixture over a period of 15 min. A dark purple color of the lithium complex **1a** quickly appears. The solution was stirred for an additional 45 min at the same temperature. After this period, a ketone **2** solution (164 mg, 0.72 mmol, 1.2 equiv) in dry THF (2 mL) was added dropwise over 1 h. The reaction mixture was stirred for an additional 15 min, and quenched by dropwise addition of a saturated aqueous solution of NH_4Cl at −78 °C (1.0 mL). Triphenylmethane (Ph_3CH, 146 mg, 0.60 mmol, 1 equiv), was added to the mixture as the NMR internal

standard, and the workup was performed using water (8 mL) and EtOAc (10 mL). Prior to NMR analysis, the organic layer was dried with anhydrous Na_2SO_4, and the solvent was evaporated under reduced pressure.

Pyrrolidine/chiral amines system (General Procedure B)

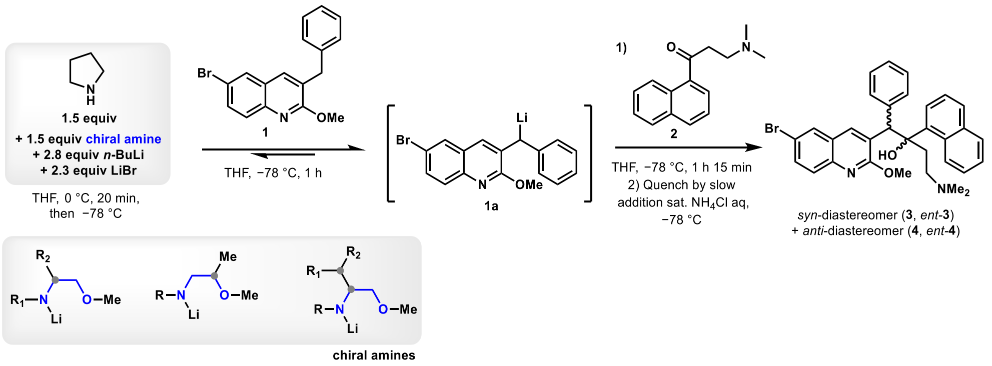

Step 1 (Neutralization of amines hydrochloride salts): In a round-bottom flask, the hydrochloride salt of the chiral amine (1.00 g, 1 equiv) was dissolved in water (10 mL), and a solution of NaOH (2.10 equiv) in water (10 mL) was added dropwise into the reaction mixture and stirred for 15 min at room temperature. DCM (20 mL) was poured into the reaction flask and vigorously stirred for an additional 15 min. The resulting mixture was transferred into a separatory funnel. The organic layer was separated and dried with anhydrous Na_2SO_4, and the solvent evaporated under reduced pressure. The concentrated free amine was dried with a high-vacuum pump for 30 min. The final product was stored under N_2 atmosphere with 4 Å activated molecular sieves (*Note*: Some of the neutralized amines present relatively low boiling points, and drying time must be reduced).

Step 2: In a glove box filled with N_2, a 40 mL oven-dry vial containing a stir bar was charged with anhydrous LiBr (120 mg, 1.38 mmol, 2.3 equiv). The vial was properly closed with a TFE-lined silicone septa cap and removed from the glove box, and a balloon containing N_2 was attached. Dry THF (2 mL) was added to this vial, and the resulting mixture was stirred at room temperature until full solubilization of the salt was achieved. Pyrrolidine (75 µL, 0.9 mmol, 1.5 equiv) and the chiral amine (0.9 mmol, 1.5 equiv) were added. The mixture was cooled to 0 °C followed by dropwise addition of *n*-BuLi (2.5 M in hexane, 2.8 equiv). The mixture was stirred for 20 min (light-yellow solution). The reaction was cooled to −78 °C, and a solution of quinoline **1** (200 mg, 0.60 mmol, 1 equiv) in dry THF (2 mL) was added dropwise into the reaction mixture over a period of 15 min. A dark purple color of the lithium complex **1a** quickly appears. The solution was stirred for an additional 45 min at the same temperature. After this period, a ketone **2**

solution (164 mg, 0.72 mmol, 1.2 equiv) in dry THF (2 mL) was added dropwise over 1 h. The reaction mixture was stirred for an additional 15 min, and quenched by dropwise addition of a saturated aqueous solution of NH_4Cl at −78 °C (1.0 mL). Triphenylmethane (Ph_3CH, 146 mg, 0.60 mmol, 1 equiv), was added to the mixture as the NMR internal standard, and the workup was performed using water (8 mL) and EtOAc (10 mL). Prior to NMR analysis, the organic layer was dried with anhydrous Na_2SO_4, and the solvent was evaporated under reduced pressure.

Chiral lithium amides system (General Procedure C)

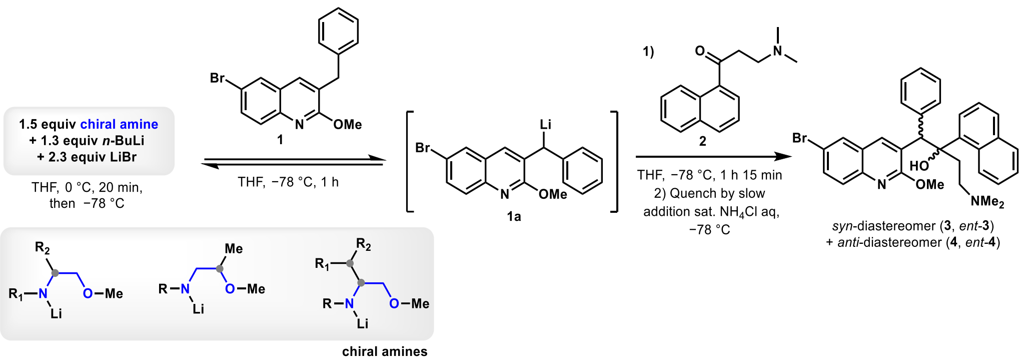

Step 1 (Neutralization of amines hydrochloride salts): *Same as General Procedure B.*

Step 2: In a glove box filled with N_2, a 40 mL oven-dry vial containing a stir bar was charged with anhydrous LiBr (120 mg, 1.38 mmol, 2.3 equiv). The vial was closed with a TFE-lined silicone septa cap and removed from the glove box, and a balloon containing N_2 was attached. Dry THF (2 mL) was added to this vial, and the resulting mixture was stirred at room temperature until full solubilization of the salt was achieved. The chiral amine (0.9 mmol, 1.5 equiv) was added, and the mixture was cooled to 0 °C followed by the dropwise addition of *n*-BuLi (2.5 M in hexane, 1.3 equiv). The mixture was stirred for 20 min (light-yellow solution) and cooled to −78 °C. A solution of quinoline **1** (200 mg, 0.60 mmol, 1 equiv) in dry THF (2 mL) was added dropwise into the reaction mixture over a period of 15 min. A dark purple color of the lithium complex **1a** quickly appears. The solution was stirred for an additional 45 min at the same temperature. After this period, a ketone **2** solution (164 mg, 0.72 mmol, 1.2 equiv) in dry THF (2 mL) was added dropwise over 1 h. The reaction mixture was stirred for an additional 15 min, and quenched by dropwise addition of a saturated aqueous solution of NH_4Cl at −78 °C (1.0 mL). Triphenylmethane (Ph_3CH, 146 mg, 0.60 mmol, 1 equiv), was added to the mixture as the NMR internal standard, and the workup was performed using water (8 mL) and EtOAc (10 mL). Prior to NMR analysis, the organic layer was dried with anhydrous Na_2SO_4, and the solvent was evaporated under reduced pressure.

***Important notes (valid for Procedures A to C)*:**

a) Reaction is extremely moisture sensitive. LiBr is hygroscopic and ideally must be weighed in a glove box filled with N_2.

b) The distillation of pyrrolidine prior to its use is recommendable, as well as its storage under an inert atmosphere with activated molecular sieves (4 Å).

c) All the chiral catalysts screened in this part of the work are commercially available. Activated molecular sieves (4 Å) were added to the liquid ones to minimize their water content.

d) Solid chiral ligands (e.g., BINOL or TADDOL) were charged in the same vial as LiBr inside the glove box prior to THF addition.

e) *n*-BuLi must be titrated often to ensure its actual concentration is known prior to its use.

f) Dry solvent was purchased from Sigma-Aldrich in Sure/Seal™ bottles.

g) Equivalents of *n*-BuLi vary depending on the ligand used: 1.3 equiv of *n*-BuLi is the fixed amount used for pyrrolidine deprotonation. An additional 1 equiv of *n*-BuLi must be added for each acidic hydrogen present in the catalyst. For example, if 1.5 equiv of BINOL is added to the reaction, an additional 3 equiv of *n*-BuLi is summed to the fixed amount of 1.3 equiv, totalizing 4.3 equiv.

Optimization experiments using (*R*)-2-(methoxymethyl)pyrrolidine (General Procedure D)

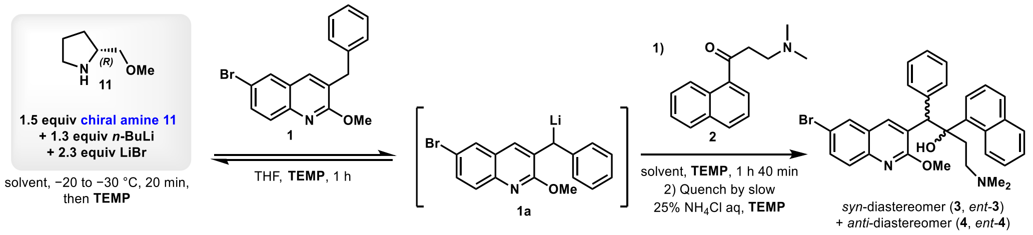

In a glove box filled with N_2, a 2-neck round-bottom flask containing a stir bar was charged with anhydrous LiBr (2.3 equiv). The flask was closed with a rubber septum and a balloon containing N_2 was attached. Dry THF (5 V) was added to the flask, and the resulting mixture was stirred at room temperature until full solubilization of the salt was achieved. The chiral amine **11** (1.5 equiv) was added, and the mixture was cooled to −20/−30 °C, followed by the dropwise addition of *n*-BuLi (1.8 M in hexane, 1.3 equiv). The mixture was stirred for 20 min. The reaction flask was cooled to −78 °C or −40 °C, and a solution of quinoline **1** (1 equiv) in dry THF (5 V) was added dropwise into the reaction mixture over a period of 15 min. A dark purple color of the lithium complex **1a** quickly appears. The solution was stirred for an

additional 45 min at the same temperature. After this period, a ketone **2** solution (1.2 equiv) in dry THF (5 V) was added dropwise over 1 h. The reaction mixture was stirred for an additional 40 min, and quenched by dropwise addition of a 25% aqueous solution of NH_4Cl at −78 °C or −40 °C (5 V). The product was extracted with DCM (2 x 5 V). An aliquot of the crude sample was submitted to HPLC analysis.

Note: *This procedure was used for experiments related to the variation of reaction concentration, solvent screening, and for analyzing the effect of different water content % in the solvent. Scale: 500 mg, 1.0 g, or 5.0 g of quinoline* ***1****.*

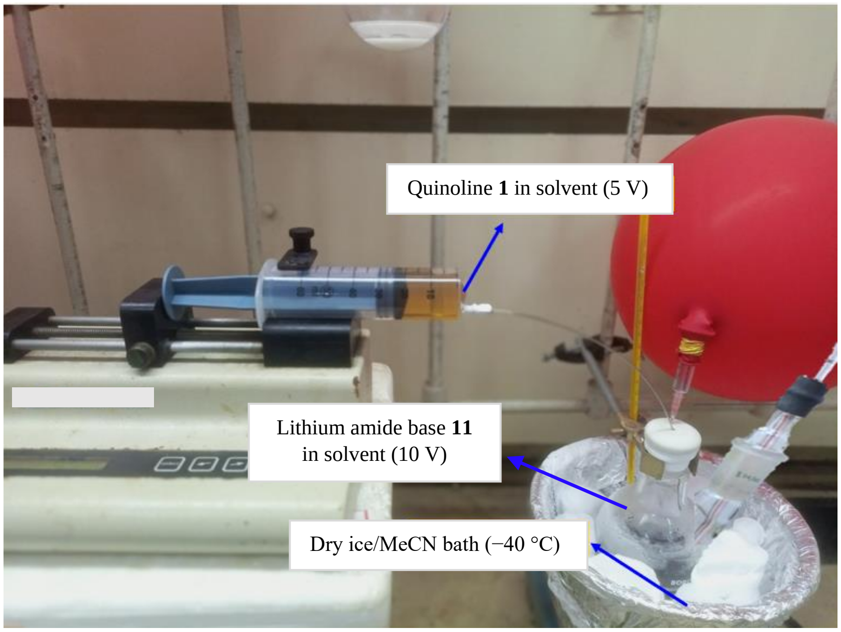

Figure S9. *Reaction setup utilized to synthesize BDQ (**3**) (500 mg to 5.0 g scale of quinoline **1**)*

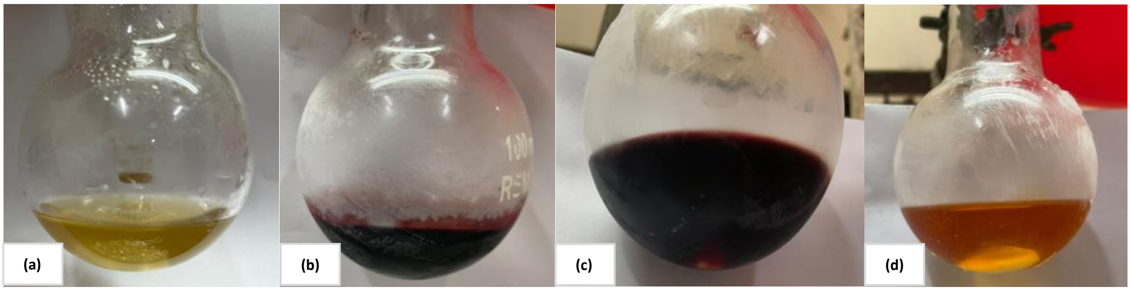

Figure S10*. Appearance of the reaction mixture at different times of the BDQ (**3**) synthesis: (a) after preparation of lithium (R)-2-(methoxymethyl)pyrrolidide; (b) during addition of quinoline **1**; (c) during addition of ketone **2**, and (d) after reaction quench with NH_4Cl aqueous solution*

Scaling-up BDQ (3) synthesis to 75 g (Procedure E)

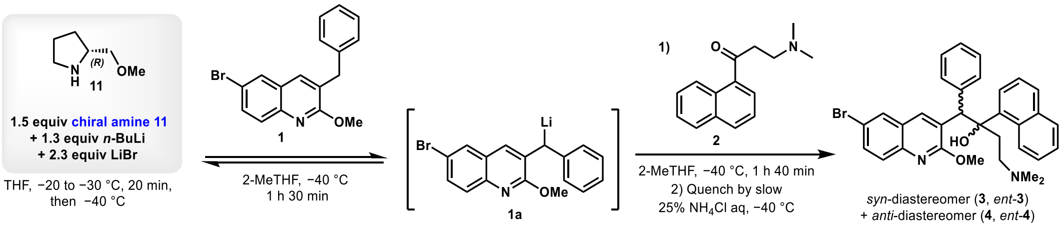

A 5 L four-neck round-bottom flask was equipped with an overhead stirrer and a thermometer for monitoring the reaction internal temperature, as well as a N_2 inlet/outlet to ensure inert atmosphere during the entire course of the reaction (see Figure S12). 2-MeTHF (450 mL, 6 V) was transferred to the 5 L flask, followed by a solution of LiBr (0.526 mol, 45.64 g, 2.3 equiv) in 2-MeTHF (225 mL, 4 V), which was dried via azeotropic distillation. The solvent and LiBr solution were transferred to the reaction flask via cannula through an addition funnel (see Figure S12). Anhydrous (*R*)-2-(methoxymethyl)pyrrolidine (**11**) (0.343 mol, 40.2 mL, 1.5 equiv) was similarly transferred to the reaction flask. The reaction mixture was cooled to −20/−30 °C, and 1.8 M of *n*-BuLi in hexanes (0.297 mol, 135.0 mL, 1.3 equiv) was added dropwise (cannula/addition funnel). After 20 min, the flask was further cooled to −40 °C, and a solution of quinoline **1** (0.228 mol, 75.0 g, 1.0 equiv, 98% purity by HPLC) in dry 2-MeTHF (300 mL, 4 V), also dried via azeotropic distillation, was transferred to the addition funnel, followed by additional 2-MeTHF (75 mL, 1 V). The quinoline **1** solution contained in the addition funnel was added to the lithium amide base solution over 1 h (cannula/addition funnel). The resulting mixture was stirred for an additional 30 min. A solution of ketone **2** (0.274 mol, 62.3 g, 1.2 equiv, 95% purity by HPLC) in dry 2-MeTHF (375 mL, 5 V) was added to the reaction mixture over 1 h at the same temperature (cannula/addition funnel). The reaction was stirred for an additional 45 min, and quenched by the dropwise addition of a 25% NH_4Cl aqueous solution (375 mL, 5 V) at −40 °C (cannula/addition funnel). The reaction mass was directly poured into a separatory funnel. The phases were separated, and the aqueous layer was extracted with DCM (2 x 375 mL, 10 V). The combined organic layers were dried with anhydrous Na_2SO_4 (20.0 g), filtered through a Büchner funnel, and the Na_2SO_4 bed was washed with DCM (75 mL, 1 V). The solvent was removed under reduced pressure at 45-50 °C to afford 136 g of crude material. Analysis of the obtained material showed that the *syn*-diastereomer pair (**3**+*ent*-**3**) was obtained in 82% assay yield, corresponding to 64% of BDQ (**3**) in the crude mixture prior to purification (based on HPLC wt % purity and SFC ratios).

Note: After distillation, (*R*)-2-(methoxymethyl)pyrrolidine (**11**) was stored under inert atmosphere and with activated molecular sieves (4 Å). In the case of ketone **2**, after neutralization of its hydrochloride salt,

compound **2** was dried under vacuum at room temperature, and after the addition of 2-MeTHF, activated molecular sieves (4 Å) were added to the solution prior to its use in the BA reaction.

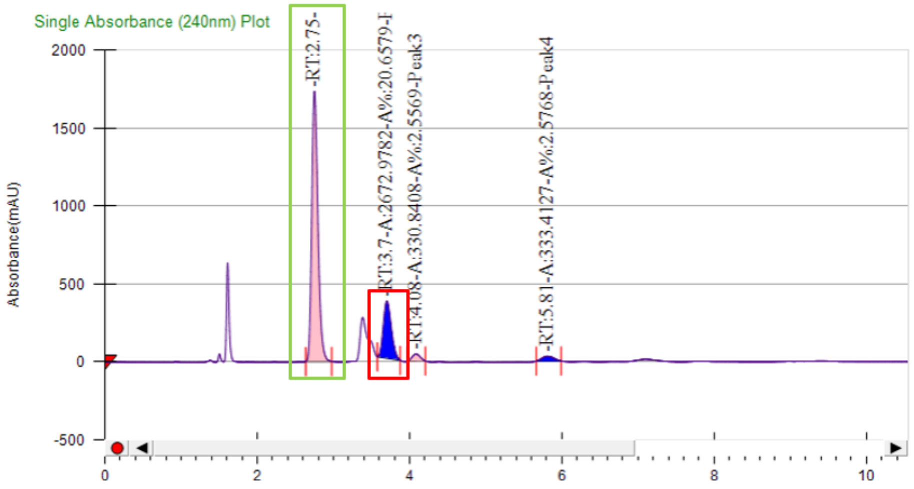

Peak Information

Peak No	% Area	Area	Ret. Time	Height	Cap. Factor
1	74.2085	9602.0412	2.75 min	1733.3967	2749
2	20.6579	2672.9782	3.7 min	367.8483	3699
3	2.5569	330.8408	4.08 min	45.6347	4082.3333
4	2.5768	333.4127	5.81 min	31.1984	5805.7833

Figure S11. *SFC analysis of crude material obtained after reaction quench (75 g batch): peak 1 = BDQ* ***3****, peak 2 = ent-**3***

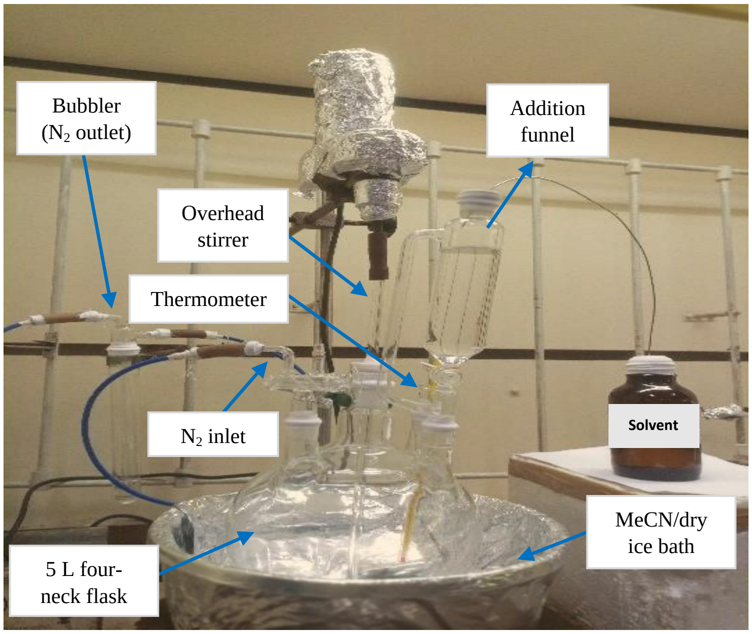

Figure S12. *Reaction setup utilized to synthesize BDQ (**3**) (75.0 g scale of quinoline **1**)*

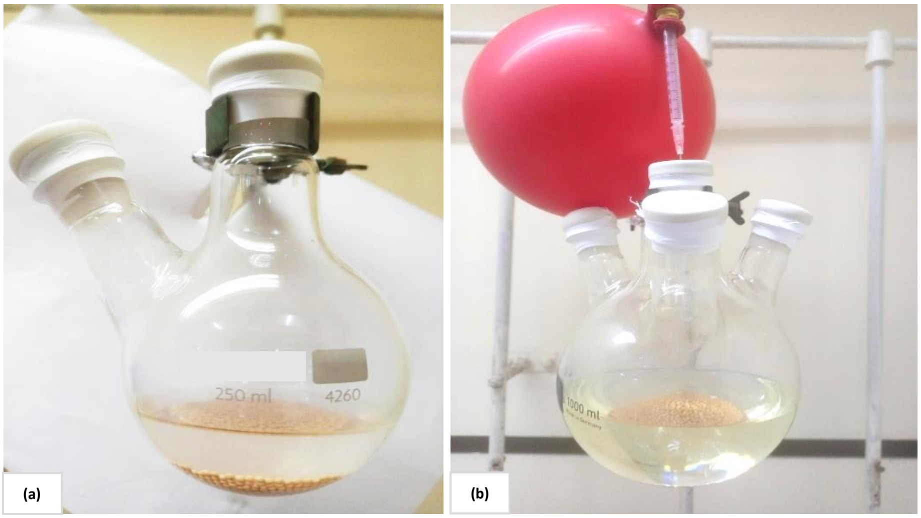

Figure S13. *Solutions of (a) (R)-2-(methoxymethyl)pyrrolidine (**11**) and (b) ketone **2** over activated molecular sieves (4 Å), and N_2 atmosphere prior to addition to the reaction flask*

GENERAL METHODS – SYNTHESIS OF CHIRAL AMINES AND MAJOR IMPURITY

Synthesis of (*R*)-2-(methoxymethyl)pyrrolidine (11)

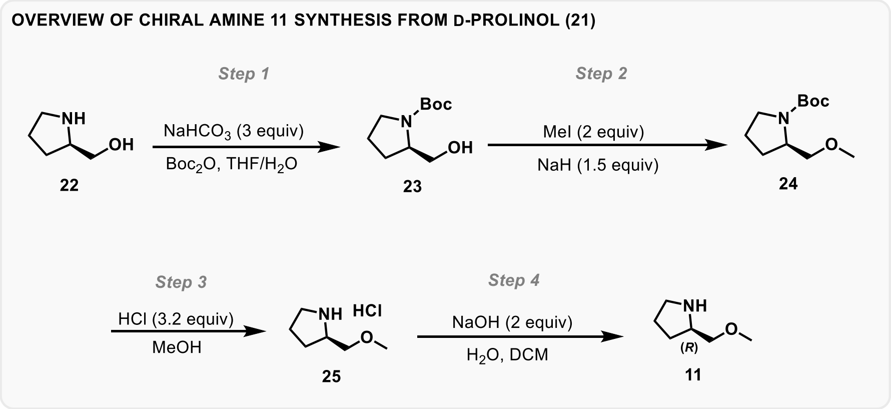

Step 1: *Amino group protection*

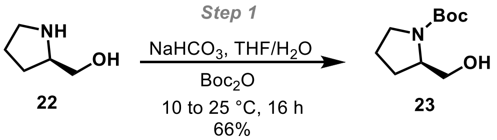

Tert-butyl (R)-2-(hydroxymethyl)pyrrolidine-1-carboxylate (23). A suspension of D-prolinol (**22**) (290.0 g, 1.0 equiv) and $NaHCO_3$ (602.1 g, 2.5 equiv) in THF (2.9 L) and H_2O (2.9 L) was cooled to 5-10 °C, and Boc_2O (938.6 g, 1.5 equiv) was slowly added to this mixture. The reaction was stirred for 12 h at 25 °C, and water (1.2 L) was added to the reaction flask. After phases separation, the organic layer was extracted with EtOAc (2 x 2.9 L). The combined organic phase was washed with brine (2.9 L), dried over anhydrous Na_2SO_4, and concentrated under vacuum to give 599.0 g of crude material. *n*-heptane (2 V, 600 mL) was charged into the flask and stirred for 12 h at 25-30 °C. Precipitation of a white solid was observed. The solid was filtered and washed with cold *n*-heptane (0.5 V), and dried under vacuum for 4-5 h at 45-50 °C resulting in 485 g of isolated mass. The assay purity of the obtained material was assessed by qNMR (78%), affording a 66% yield of **23**. NMR data is in accordance with the literature.[6]

Note: The moderate yield is due to compound **23** losses in the mother liquor (this step needs to be further optimized).

Step 2: *O-Methylation with Iodomethane*

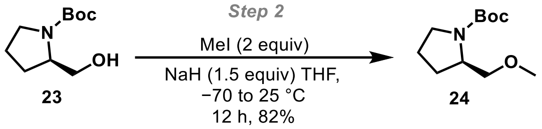

Tert-butyl (R)-2-(methoxymethyl)pyrrolidine-1-carboxylate (24). To a solution of compound **23** (480.0 g, 78% purity, 1.0 equiv) and MeI (677 g, 2.0 equiv) in THF (2.9 L), NaH (60% dispersion in mineral oil) (143 g, 1.5 equiv) was added portion-wise at 0-5 °C. The suspension was warmed to 25 °C and stirred at 25 °C for 12 h, and a saturated NH_4Cl solution (2.9 L) was slowly added to the reaction mixture. After phase separation, the aqueous layer was extracted with EtOAc (3 x 2.9 L). The combined organic phase was washed with brine (2 x 2.9 L), dried over anhydrous Na_2SO_4, filtered, and concentrated under vacuum to give 514.0 g of crude material. The assay purity of the obtained material was assessed by qNMR (95%), affording an 82% yield of **24**. Compound **24** was used without further purification in the next step. NMR data is in accordance with the literature.[7]

Step 3: *Removal of protecting group*

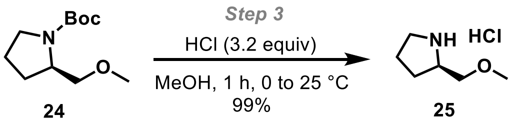

(R)-2-(methoxymethyl)pyrrolidine hydrochloride (25). To a stirred solution of HCl in MeOH (4 M, 1.8 L, 3.2 equiv) at 0 °C, compound **24** (505.0 g, 95% purity, 1.0 equiv) was added, and the resulting solution was

stirred for 2 h at 25 °C. After completion of the reaction, the solvent was removed under reduced pressure to give compound **25** as a light-yellow solid (383.0 g). The assay purity of the material was assessed by qNMR (87%), affording a 99% yield of **25**. Compound **25** was used without further purification in the next step.

Step 4: *Hydrochloride salt neutralization*

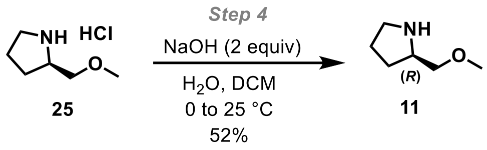

(R)-2-(methoxymethyl)pyrrolidine (11): The hydrochloride salt **25** (380.0 g, 87% purity, 1.0 equiv) was dissolved in water (4 V), and a solution of NaOH (2.1 equiv) in water (10 V) was added dropwise into the reaction mixture at 0 °C and stirred for 15 min at room temperature. DCM (3 x 5 V) was poured into the reaction flask and vigorously stirred for an additional 15 min. The biphasic reaction mixture was transferred to a separatory funnel, and the organic layer was separated, dried over anhydrous Na_2SO_4, and concentrated under reduced pressure to provide 130.0 g of isolated crude material. The assay purity of the obtained material was assessed by qNMR (94%), affording a 52% yield of **11**. NMR data is in accordance with the literature.[8]

Note: The low yield is due to amine **11** losses in the aqueous phase (this step needs to be further optimized).

Distillation of (R)-2-(methoxymethyl)pyrrolidine (11). Distillation was performed to achieve the pure chiral amine **11**. The crude material obtained after hydrochloride salt neutralization was distilled under vacuum (oil bath temperature was around 110 °C). The first fraction was collected at vapor temperature of 30-35 °C at 0.4 mmHg. This fraction comprises the desired compound **11** with purity >99% (HPLC A%). A second fraction (dark brown colored) was collected at vapor temperatures of 37-42 °C at 0.4 mmHg. According to the ^{1}H NMR analysis, this fraction contains a small amount of product along with other impurities. The first fraction was stored under argon atmosphere with activated molecular sieves (4 Å). When starting from 130 g of the non-purified chiral amine **11**, around 68 g of pure material was isolated after distillation (52% yield, based on mass). The mass of the residue left in the flask after distillation was 30.0 g, while the mass of chiral amine captured in the cold trap was ~25.0 g (can be purified in the next round of distillation).

Note: *It is important to check the purity of the isolated chiral amine by GCHS, to ensure no other volatile impurities are present in the collected sample prior to its use in the BDQ (**3**) synthesis. Further optimization for the distillation purification will be developed.*

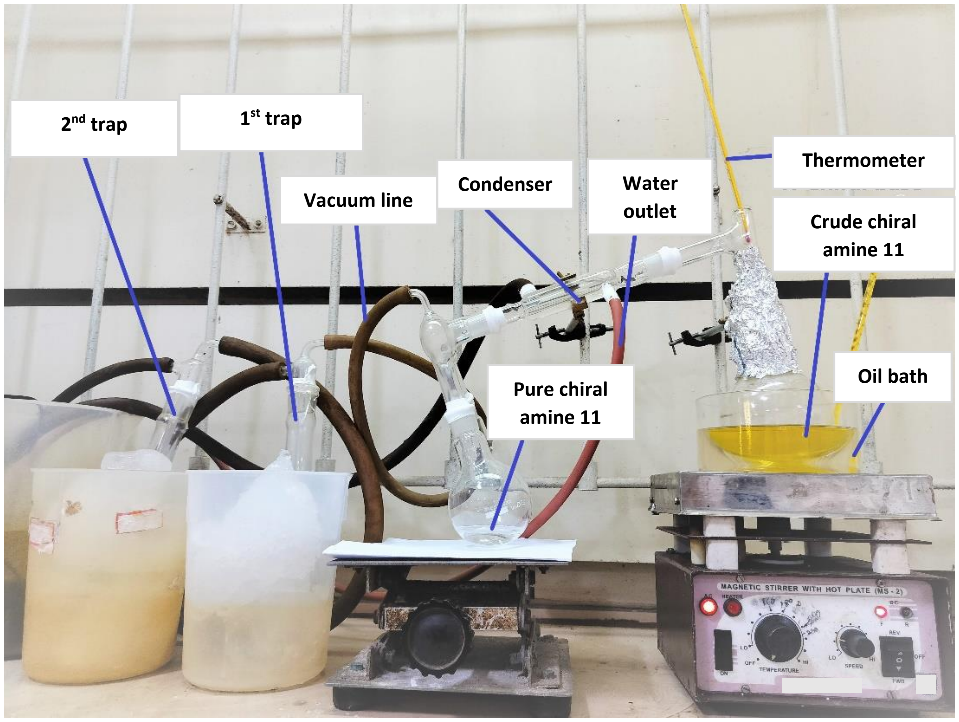

Figure S14. *Setup used for vacuum distillation of chiral amine* ***11***

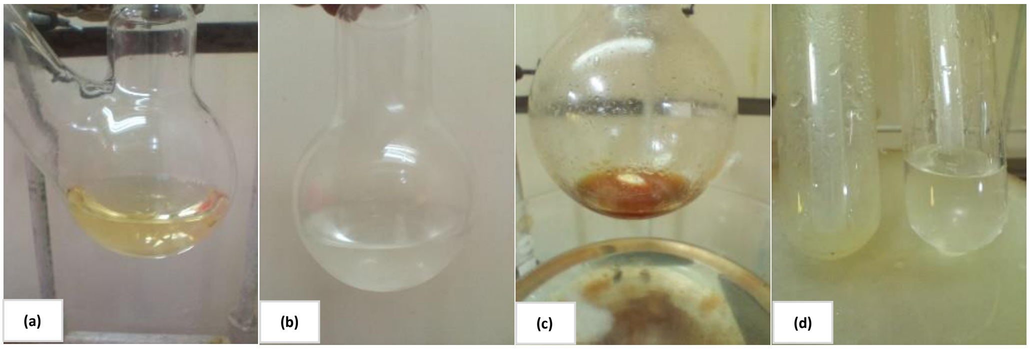

Figure S15. *Vacuum distillation of (R)-2-(methoxymethyl)pyrrolidine (**11**): (a) crude amine prior to distillation; (b) First fraction of **11** collected (>99% by HPLC); (c) Brown residue obtained by the end of distillation; (d) Chiral amine **11** vapor captured in the traps (containing other volatile impurities)*

Note: Although the procedures described above were adopted to produce (*R*)-2-(methoxymethyl)pyrrolidine (**11**) for the BA reaction scale-up, M4ALL's techno-economic analysis had shown the use of D-proline as starting material is essential to make this asymmetric approach more cost-effective than our previously published racemic methodology.[2i] The in-house production of **11** from D-proline is recommended due to the high values some vendors can charge for more advanced intermediates or the amine **11** itself, which have a significant impact on the total raw materials cost. After analyzing different methodologies in the opened literature, we identified a more straightforward and lower-cost 4-

step telescoped process covering the transformation of L-proline to (*S*)-2-(methoxymethyl)pyrrolidine in 70% overall yield (Scheme S6).[8, 9] Switching L-proline with D-proline should not cause considerable variation in the reaction outcome. Therefore, this 4-step sequence becomes a valuable option to obtain **11** as cheaply as possible.

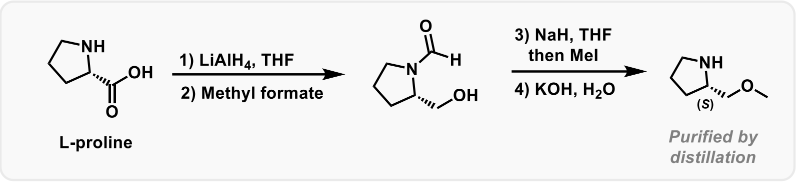

Scheme S6. *Four-step process for the synthesis of (S)-2-(methoxymethyl)pyrrolidine from L-proline*

Synthesis of the acyclic chiral amines 13 and 14

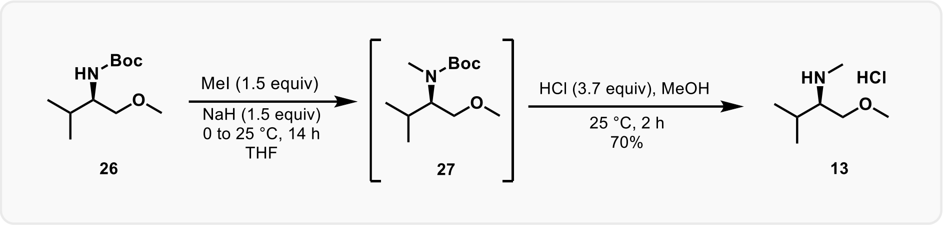

(R)-1-methoxy-N,3-dimethylbutan-2-amine hydrochloride (13). To a suspension of NaH (2.38 g, 59.62 mmol, 60% purity, 1.5 equiv) in THF (100 mL) at 0 °C, a solution of compound **26** (8.64 g, 39.75 mmol, 1.0 equiv) in THF (100 mL) was added dropwise. The suspension was stirred for 2 h at 25 °C under N_2 atmosphere. The reaction was re-cooled to 0 °C, and MeI (8.46 g, 59.62 mmol, 1.5 equiv) was added. The suspension was warmed to 25 °C and stirred for 12 h. After the consumption of all the starting material, a saturated NH_4Cl solution (100 mL) was slowly added to the resulting mixture. The aqueous phase was extracted with EtOAc (3 x 100 mL). The combined organic phase was washed with brine (2 x 100 mL), dried over anhydrous Na_2SO_4, filtered, and concentrated under reduced pressure to give the crude intermediate **27** (light-yellow oil). The obtained mass was solubilized in MeOH (20 mL), and HCl in MeOH (4 M, 30 mL, 3.7 equiv) was added. The reaction was stirred for 2 h at 25 °C, and approximately 80% of the solvent volume was removed under vacuum. The white solid was filtered and washed with cold MeOH (3 x 10 mL). After drying, the hydrochloride salt **13** was achieved in 70% yield (4.66 g, 27.82 mmol).

^{1}H NMR (600 MHz, methanol-*d₄*): δ/ppm = 3.69 (dd, *J* = 11.1, 3.5 Hz, 1H), 3.61 (dd, *J* = 11.1, 6.1 Hz, 1H), 3.41 (s, 3H), 3.31 (dt, *J* = 3.2, 1.6 Hz, 1H), 3.11 - 3.09 (m, 1H), 2.72 (s, 3H), 2.18 - 2.09 (m, 1H), 1.08 (d, *J* = 6.9 Hz, 3H), 1.03 (d, *J* = 6.9 Hz, 3H).

^{13}C NMR (150 MHz, methanol-*d₄*): δ/ppm = 68.7, 65.1, 59.4, 32.1, 28.7, 19.7, 18.0.

HRMS (ESI) *m/z*: $[M + H]^+$ Calcd for $C_7H_{18}NO$ 132.1388; Found 132.1386.

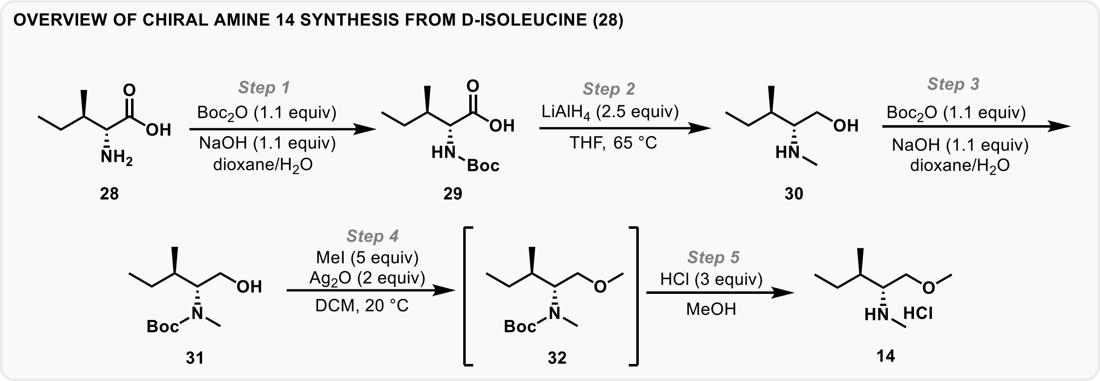

Step 1: *Amino group protection*

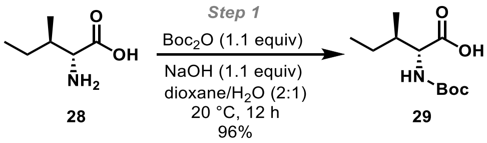

(Tert-butoxycarbonyl)-D-isoleucine (29). To a suspension of the amino acid **28** (5.0 g, 38.12 mmol, 1.0 equiv) and NaOH (1.68 g, 41.93 mmol, 1.1 equiv) in dioxane (60 mL) and H_2O (30 mL) at 0 °C, Boc_2O (9.15 g, 41.93 mmol, 1.1 equiv) was added. The suspension was stirred for 12 h at 20 °C. The suspension was concentrated under reduced pressure to remove the dioxane. The resulting suspension was extracted with EtOAc (3 x 100 mL). The combined organic phase was discarded. The aqueous phase was adjusted to pH 5 with citric acid, and the extraction was performed with EtOAc (3 x 100 mL). The combined organic phase was washed with brine (2 x 100 mL), dried over anhydrous Na_2SO_4, filtered, and concentrated under vacuum to give the product **29** as a colorless oil (8.5 g, 96% yield).

^{1}H NMR (600 MHz, DMSO-*d*$_6$): δ/ppm = 12.44 (br s, 1H), 6.81 (br d, *J* = 8.8 Hz, 1H), 4.02 - 3.95 (m, 1H), 1.89 - 1.73 (m, 1H), 1.38 (s, 9H), 1.37 - 1.27 (m, 1H), 1.20 - 1.11 (m, 1H), 0.89 - 0.76 (m, 6H). NMR data is in accordance with the literature.[10]

Step 2: *Carboxylic acid reduction and N-methylation*

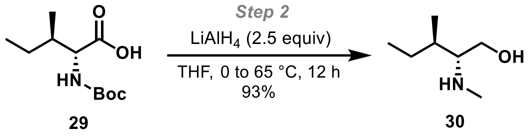

(2R,3R)-3-methyl-2-(methylamino)pentan-1-ol (30). To a suspension of $LiAlH_4$ (3.49 g, 91.88 mmol, 2.5 equiv) in THF (150 mL) at 0 °C, a solution of compound **29** (8.5 g, 36.75 mmol, 1.0 equiv) in THF (50 mL) was added dropwise. The suspension was stirred for 12 h at 65 °C and under N_2 atmosphere. Water (3.5 mL) was added to the solution dropwise. A 15% NaOH solution (3.5 mL) was added to the reaction mixture, followed by the addition of water (10.5 mL). The resulting mixture was extracted with EtOAc (3 x 100 mL). The combined organic phase was washed with brine (2 x 100 mL), dried over anhydrous Na_2SO_4, filtered, and concentrated under vacuum to give the product **30** as a light-yellow oil (4.5 g, 93% yield).

^{1}H NMR (600 MHz, $CDCl_3$): δ/ppm = 3.59 (dd, *J* = 4.4, 10.4 Hz, 1H), 3.32 (dd, *J* = 7.2, 10.4 Hz, 1H), 2.46 - 2.32 (m, 4H), 1.65 - 1.51 (m, 1H), 1.50 - 1.44 (m, 1H), 1.12 - 0.99 (m, 1H), 0.92 - 0.85 (m, 6H).

Step 3: *Amino group protection*

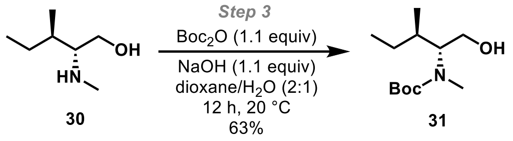

Tert-butyl ((2R,3R)-1-hydroxy-3-methylpentan-2-yl)(methyl)carbamate (31). To a solution of compound **30** (4.5 g, 34.29 mmol, 1.0 equiv) and NaOH (1.51 g, 37.72 mmol, 1.1 equiv) in dioxane (60 mL) and H_2O (30 mL) at 0 °C, Boc_2O (8.23 g, 37.72 mmol, 1.1 equiv) was added. The solution was stirred for 12 h at 20 °C, and concentrated under reduced pressure. The aqueous phase was extracted with EtOAc (3 x 100 mL). The combined organic phase was washed with brine (2 x 100 mL), dried over anhydrous Na_2SO_4, filtered, and concentrated under vacuum to give the product **31** as a light-yellow oil (5.0 g, 63% yield).

^{1}H NMR (600 MHz, $CDCl_3$): δ/ppm = 3.91 - 3.52 (m, 3H), 2.76 (s, 3H), 2.52 (br s, 1H), 1.73 (br s, 1H), 1.46 (s, 9H), 1.54 - 1.44 (m, 1H), 1.21 - 1.08 (m, 1H), 0.90 (t, *J* = 7.2 Hz, 3H), 0.84 (d, *J* = 6.8 Hz, 3H). NMR data is in accordance with the literature.[11]

Steps 4 and 5: *O-Methylation with iodomethane and removal of protecting group*

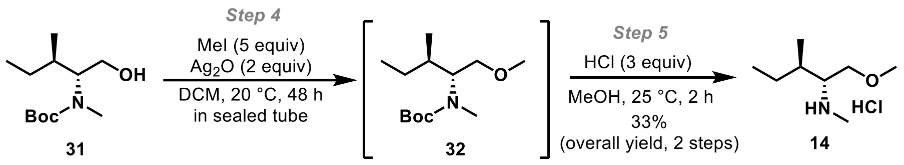

(2R,3R)-1-methoxy-N,3-dimethylpentan-2-amine hydrochloride (14**).** A suspension of carbamate **31** (3.5 g, 15.13 mmol, 1.0 equiv) and Ag_2O (7.01 g, 30.26 mmol, 2.0 equiv) in DCM (50 mL) was prepared in a sealed tube, and MeI (10.74 g, 75.65 mmol, 5.0 equiv) was added. The suspension was stirred for 48 h at 20 °C under N_2 atmosphere in the dark (sealed tube covered with aluminum foil). The reaction was filtered, and the filtrate diluted with water (50 mL). The organic phase was separated, and the aqueous phase was extracted with DCM (3 x 50 mL). The combined organic phase was washed with brine (2 x 50 mL), dried over anhydrous Na_2SO_4, filtered, and concentrated under reduced pressure. A solution of the crude compound **32** in MeOH (10 mL) was prepared, and a HCl solution in MeOH (4 M, 4.59 mL, 3.0 equiv) was added. The reaction mixture was stirred for 2 h at 25 °C. The resulting solution was concentrated under reduced pressure to give the product **14** as a light-yellow solid (0.9 g, 4.95 mmol, 33% overall yield).

^{1}H NMR (600 MHz, methanol-*d₄*): δ/ppm = 3.72 - 3.65 (m, 1H), 3.63 - 3.57 (m, 1H), 3.41 (s, 3H), 3.21 - 3.15 (m, 1H), 2.73 (s, 3H), 1.94 - 1.80 (m, 1H), 1.63 - 1.50 (m, 1H), 1.31 - 1.16 (m, 1H), 1.11 - 1.01 (m, 3H), 1.00 - 0.93.

^{13}C NMR (150 MHz, methanol-*d₄*): δ/ppm = 69.2, 64.8, 59.4, 35.6, 32.5, 25.4, 15.7, 11.8.

HRMS (ESI) *m/z*: $[M + H]^+$ Calcd for $C_8H_{20}NO$ 146.1545; Found 146.1538.

Synthesis of 1,4-Michael addition side product 20

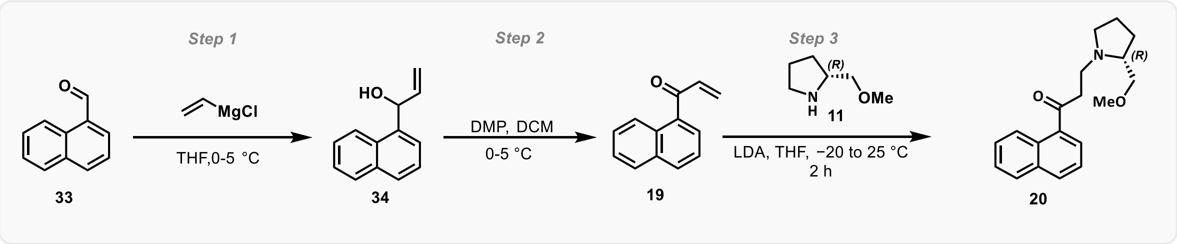

Step 1: *Reaction of 1-naphthaldehyde with vinyl magnesium bromide*

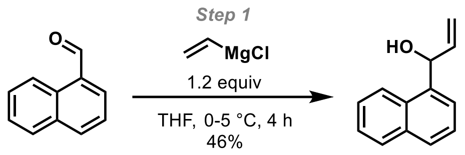

1-(naphthalen-1-yl)prop-2-en-1-ol (34): To a stirred solution of 1-naphthaldehyde (**33**) (15 g, 96.04 mmol, 1 equiv) in THF (5 V), vinyl magnesium bromide solution (1 M) (115.25 mL, 1.2 equiv) was added dropwise at 0-5 °C under N_2 atmosphere. The reaction mixture was stirred at this temperature for 4 h. After reaction completion (determined by TLC), a saturated solution of NH_4Cl (420 mL) was slowly added at 0 °C. The aqueous phase was extracted with EtOAc (3 x 420 mL). The combined organic phase was washed with water (500 mL) followed by brine (400 mL). After phase separation, the organic layer was dried over anhydrous Na_2SO_4, filtered, and concentrated under vacuum to give a residue which was purified by flash silica gel chromatography (100-200 silica gel, eluent: 10% EtOAc/hexanes). Compound **34** was obtained as a light-yellow oil in 46% yield (8.2 g). NMR data is in accordance with the literature.[12]

Step 2: *Alcohol reduction to ketone*

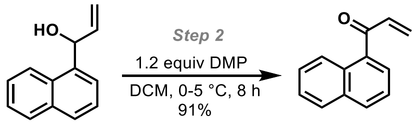

1-(naphthalen-1-yl)prop-2-en-1-one (19). A stirred solution of compound **34** (8.0 g, 43.42 mmol, 1 equiv) in DCM (80 mL, 10V) was cooled to 0-5 °C under N_2 atmosphere. Dess-Martin periodinane (22.1 g, 52.10 mmol, 1.2 equiv) was added portion wise to the reaction mass at the same temperature and stirred at 25-30 °C for 8 h. The reaction was quenched with $Na_2S_2O_3$ (Hypo solution, 250 mL) at 0 °C, and the resulting mixture stirred for an additional 30 min. The reaction mixture was filtered over a Celite® bed, which was washed with DCM (3 x 24 mL). The organic layer of the biphasic filtrate was separated, and the aqueous phase was extracted with DCM (3 x 80 mL). The combined organic phase was washed with water (80 mL), followed by brine (80 mL), dried over anhydrous Na_2SO_4, filtered, and concentrated under vacuum to give the desired compound **19** as a reddish gummy material (7.2 g, 91% yield). This material was used in the next step without further purification. NMR data is in accordance with the literature.[13]

Step 3: *1,4-Michael addition*

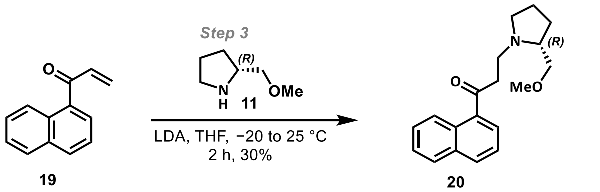

(R)-3-(2-(methoxymethyl)pyrrolidin-1-yl)-1-(naphthalen-1-yl)propan-1-one (20). A solution of (*R*)-2-(methoxymethyl)pyrrolidine (**11**) (0.948 g, 1.5 equiv) in THF (5 V) was cooled down to −20 °C, and LDA (1 M) (7.13 mL, 1.3 equiv) was slowly added to the reaction mixture under N_2 atmosphere, and stirred for 30 min. A solution of compound **19** (1.0 g, 5.48 mmol, 1.0 equiv) in THF (5 V) was added at −20 °C, and stirred at 25-30 °C for 2 h. After completion of the reaction (determined by TLC), a saturated solution of NH_4Cl (10 mL) was slowly added at 0 °C. The layers were separated, and the aqueous phase was extracted with EtOAc (3 x 100 mL). The combined organic phase was washed with water (100 mL) followed by brine (100 mL), dried over anhydrous Na_2SO_4, filtered, and concentrated under vacuum. The obtained residue was purified by flash silica gel chromatography (100-200 silica gel, eluent: 10% EtOAc/hexanes), yielding the desired product **20** (0.500 g, 30% yield, 83% purity based on HPLC A%) as light-yellow oil.

^{1}H NMR (400 MHz, $CDCl_3$): δ/ppm = 1.59 - 1.53 (m, 1H), 1.73 - 1.68 (m, 2H), 1.88 - 1.81 (m, 1H), 2.27 - 2.20 (m, 1H), 2.61 - 2.58 (m, 1H), 2.77 - 2.71 (m, 1H), 3.26 - 3.10 (m, 8H), 3.40 - 3.33 (m, 1H), 7.57 - 7.46 (m, 3H), 7.86 - 7.84 (m, 2H), 7.97 - 7.95 (m, 1H), 8.55 - 8.53 (m, 1H).
^{13}C NMR (100 MHz, $CDCl_3$): δ/ppm = 204.1, 136.2, 134.0, 132.4, 130.3, 128.4, 127.8, 127.3, 126.5, 126.0, 124.4, 75.9, 63.4, 59.0, 54.5, 50.7, 41.6, 28.4, 23.1.
HRMS (ESI) *m/z*: $[M + H]^+$ Calcd for $C_{19}H_{24}NO_2$ 298.1807; Found 298.1801.

INITIAL SCREENING OF CHIRAL LIGANDS

Non-amino acid derived chiral ligands – Use of lithium pyrrolidide as the base

Other common chiral ligands, not necessarily containing the N-C-C-O moiety, were also studied. (+)-Dimethyl L-tartrate and (*R,R)*-TADDOL were tested, but they had no effect on either d.r. or e.r. values. This occurred with (*S)*-dimethoxy BINOL, (*S)*-BINAP, (1*S*,2*S)*-cyclohexadiamine, (+)-sparteine, quinine, and quinidine as well. (*S*)-BINOL was also studied, and it was shown to improve enantioselectivity towards

BDQ (**3**) (28% ee). Although the change in d.r. was small for this case, the overall yield was good (76%), and this might constitute a system to be further explored in future work (Scheme S7).

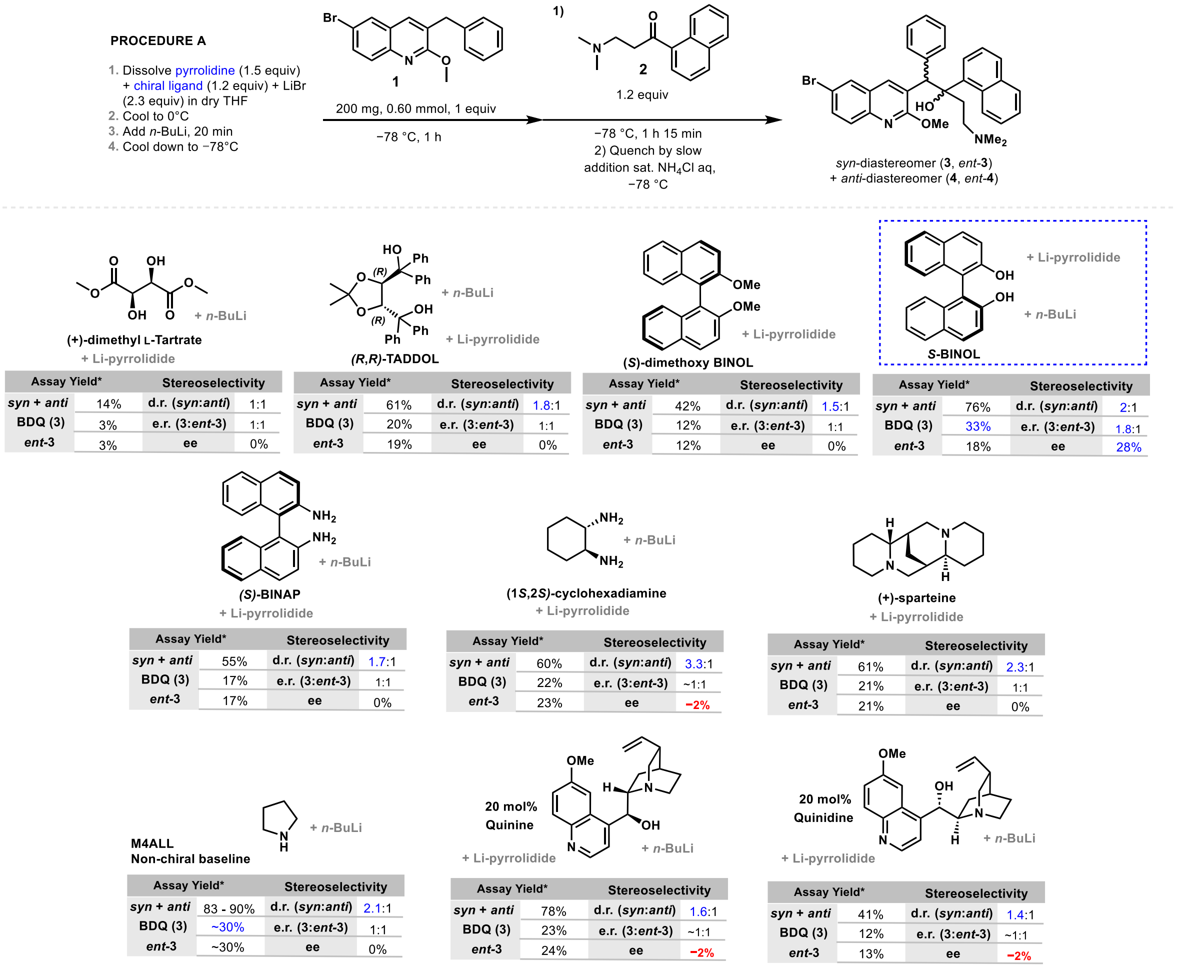

Ligand	Assay Yield*		Stereoselectivity	
(+)-dimethyl L-Tartrate	*syn + anti*	14%	d.r. (*syn*:*anti*)	1:1
	BDQ (3)	3%	e.r. (3:*ent*-3)	1:1
	ent-3	3%	ee	0%
(*R*,*R*)-TADDOL	*syn + anti*	61%	d.r. (*syn*:*anti*)	1.8:1
	BDQ (3)	20%	e.r. (3:*ent*-3)	1:1
	ent-3	19%	ee	0%
(*S*)-dimethoxy BINOL	*syn + anti*	42%	d.r. (*syn*:*anti*)	1.5:1
	BDQ (3)	12%	e.r. (3:*ent*-3)	1:1
	ent-3	12%	ee	0%
S-BINOL	*syn + anti*	76%	d.r. (*syn*:*anti*)	2:1
	BDQ (3)	33%	e.r. (3:*ent*-3)	1.8:1
	ent-3	18%	ee	28%
(*S*)-BINAP	*syn + anti*	55%	d.r. (*syn*:*anti*)	1.7:1
	BDQ (3)	17%	e.r. (3:*ent*-3)	1:1
	ent-3	17%	ee	0%
(1*S*,2*S*)-cyclohexadiamine	*syn + anti*	60%	d.r. (*syn*:*anti*)	3.3:1
	BDQ (3)	22%	e.r. (3:*ent*-3)	~1:1
	ent-3	23%	ee	−2%
(+)-sparteine	*syn + anti*	61%	d.r. (*syn*:*anti*)	2.3:1
	BDQ (3)	21%	e.r. (3:*ent*-3)	1:1
	ent-3	21%	ee	0%
M4ALL Non-chiral baseline	*syn + anti*	83 - 90%	d.r. (*syn*:*anti*)	2.1:1
	BDQ (3)	~30%	e.r. (3:*ent*-3)	1:1
	ent-3	~30%	ee	0%
20 mol% Quinine	*syn + anti*	78%	d.r. (*syn*:*anti*)	1.6:1
	BDQ (3)	23%	e.r. (3:*ent*-3)	~1:1
	ent-3	24%	ee	−2%
20 mol% Quinidine	*syn + anti*	41%	d.r. (*syn*:*anti*)	1.4:1
	BDQ (3)	12%	e.r. (3:*ent*-3)	~1:1
	ent-3	13%	ee	−2%

* *Assay yield based on qNMR of the crude reaction mixture obtained after quenching. Triphenylmethane was used as the internal standard*

Scheme S7. *Screening of chiral ligands in combination with lithium pyrrolidide*

Boc-protected amino esters – Use of lithium pyrrolidide as the base

We wanted to evaluate if the use of amino esters possessing a bulky protecting group (Boc) would influence the enantioselectivity in a positive manner. Amino esters derived from natural L-Valine and L-Phenylalanine were tested but without success; no considerable change in d.r. and e.r. was observed (Scheme S8).

PROCEDURE A

1. Dissolve pyrrolidine (1.5 equiv) + chiral ligand (1.2 equiv) + LiBr (2.3 equiv) in dry THF
2. Cool to 0°C
3. Add *n*-BuLi (2.5 equiv), 20 min
4. Cool down to −78°C

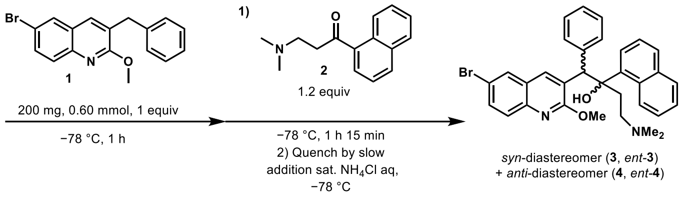

M4ALL Non-chiral baseline

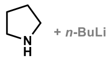

Assay Yield*		Stereoselectivity	
syn + anti	83 - 90%	d.r. (*syn*:*anti*)	2.1:1
BDQ (3)	~30%	e.r. (3:*ent*-3)	1:1
ent-3	~30%	ee	0%

Methyl (*tert*-butoxycarbonyl) *L*-valinate

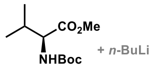

Assay Yield*		Stereoselectivity	
syn + anti	59%	d.r. (*syn*:*anti*)	1:2.1
BDQ (3)	12%	e.r. (3:*ent*-3)	1:1.2
ent-3	12%	ee	−10%

Methyl (*tert*-butoxycarbonyl) *L*-phenylalaninate

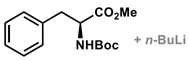

Assay Yield*		Stereoselectivity	
syn + anti	60%	d.r. (*syn*:*anti*)	1:1.4
BDQ (3)	12%	e.r. (3:*ent*-3)	1:1
ent-3	12%	ee	0%

** Assay yield based on qNMR of the crude reaction mixture obtained after quenching. Triphenylmethane was used as the internal standard*

Scheme S8. *Screening of chiral ligands in combination with lithium pyrrolidide – use of Boc-protected amino esters*

Combination of chiral amino alcohols with lithium pyrrolidide

Use of chiral ligands described in the patent filed by Fujian Institute of Microbiology (FIM)[2d] in combination with M4ALL's non-asymmetric baseline conditions developed in our previous work (Scheme S9).[2i]

PROCEDURE A

1. Dissolve pyrrolidine (1.5 equiv) + chiral ligand (1.2 equiv) + LiBr (2.3 equiv) in dry THF
2. Cool to 0°C
3. Add *n*-BuLi (2.3 equiv), 20 min
4. Cool down to −78°C

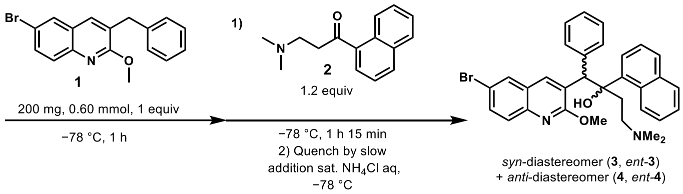

M4ALL Non-chiral baseline

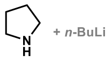

Assay Yield*		Stereoselectivity	
syn + anti	83 - 90%	d.r. (*syn*:*anti*)	2.1:1
BDQ (3)	~30%	e.r. (3:*ent*-3)	1:1
ent-3	~30%	ee	0%

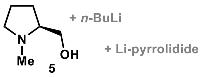

Assay Yield*		Stereoselectivity	
syn + anti	59%	d.r. (*syn*:*anti*)	2.3:1
BDQ (3)	20%	e.r. (3:*ent*-3)	1:1.1
ent-3	21%	ee	−4%

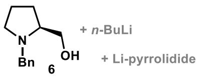

Assay Yield*		Stereoselectivity	
syn + anti	44%	d.r. (*syn*:*anti*)	2.1:1
BDQ (3)	12%	e.r. (3:*ent*-3)	1:1.5
ent-3	18%	ee	−20%

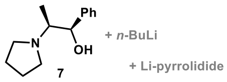

Assay Yield*		Stereoselectivity	
syn + anti	45%	d.r. (*syn*:*anti*)	2:1
BDQ (3)	16%	e.r. (3:*ent*-3)	1.2:1
ent-3	14%	ee	8%

** Assay yield based on qNMR of the crude reaction mixture obtained after quenching. Triphenylmethane was used as the internal standard*

Scheme S9. *Combining M4ALL's previous findings with the use of chiral lithium alkoxides*

Combination of acyclic amino acids derivatives as chiral ligands with lithium pyrrolidide

Group D (Scheme S10) displays two cyclic structures containing tetrahydrofuran rings, which are not derived from amino acids but possess the same N-C-C-O substructure, so we decided to test them as well. For simplicity and time-saving, all the chiral amines displayed in Scheme S10 were acquired from commercial sources, except compounds **13**, **14**.

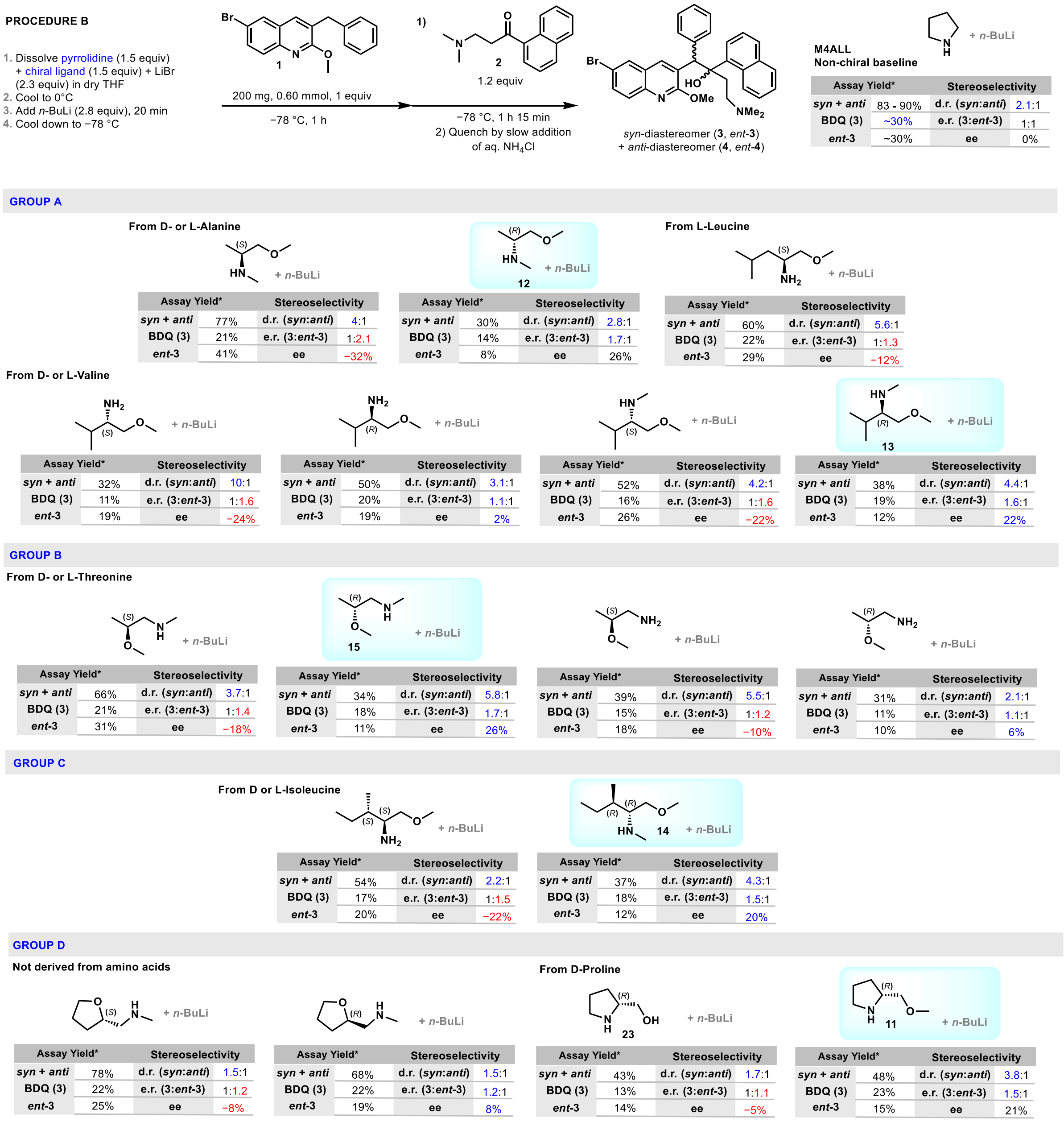

M4ALL Non-chiral baseline (pyrrolidine + n-BuLi)

Assay Yield*		Stereoselectivity	
syn + anti	83 - 90%	d.r. (syn:anti)	2.1:1
BDQ (3)	~30%	e.r. (3:ent-3)	1:1
ent-3	~30%	ee	0%

GROUP A

From D- or L-Alanine, (S) amine + n-BuLi

Assay Yield*		Stereoselectivity	
syn + anti	77%	d.r. (syn:anti)	4:1
BDQ (3)	21%	e.r. (3:ent-3)	1:2.1
ent-3	41%	ee	−32%

12 + n-BuLi

Assay Yield*		Stereoselectivity	
syn + anti	30%	d.r. (syn:anti)	2.8:1
BDQ (3)	14%	e.r. (3:ent-3)	1.7:1
ent-3	8%	ee	26%

From L-Leucine + n-BuLi

Assay Yield*		Stereoselectivity	
syn + anti	60%	d.r. (syn:anti)	5.6:1
BDQ (3)	22%	e.r. (3:ent-3)	1:1.3
ent-3	29%	ee	−12%

From D- or L-Valine

(S) primary amine + n-BuLi

Assay Yield*		Stereoselectivity	
syn + anti	32%	d.r. (syn:anti)	10:1
BDQ (3)	11%	e.r. (3:ent-3)	1:1.6
ent-3	19%	ee	−24%

(R) primary amine + n-BuLi

Assay Yield*		Stereoselectivity	
syn + anti	50%	d.r. (syn:anti)	3.1:1
BDQ (3)	20%	e.r. (3:ent-3)	1.1:1
ent-3	19%	ee	2%

(S) secondary amine + n-BuLi

Assay Yield*		Stereoselectivity	
syn + anti	52%	d.r. (syn:anti)	4.2:1
BDQ (3)	16%	e.r. (3:ent-3)	1:1.6
ent-3	26%	ee	−22%

13 + n-BuLi

Assay Yield*		Stereoselectivity	
syn + anti	38%	d.r. (syn:anti)	4.4:1
BDQ (3)	19%	e.r. (3:ent-3)	1.6:1
ent-3	12%	ee	22%

GROUP B

From D- or L-Threonine

(S) secondary amine + n-BuLi

Assay Yield*		Stereoselectivity	
syn + anti	66%	d.r. (syn:anti)	3.7:1
BDQ (3)	21%	e.r. (3:ent-3)	1:1.4
ent-3	31%	ee	−18%

15 + n-BuLi

Assay Yield*		Stereoselectivity	
syn + anti	34%	d.r. (syn:anti)	5.8:1
BDQ (3)	18%	e.r. (3:ent-3)	1.7:1
ent-3	11%	ee	26%

(S) primary amine + n-BuLi

Assay Yield*		Stereoselectivity	
syn + anti	39%	d.r. (syn:anti)	5.5:1
BDQ (3)	15%	e.r. (3:ent-3)	1:1.2
ent-3	18%	ee	−10%

(R) primary amine + n-BuLi

Assay Yield*		Stereoselectivity	
syn + anti	31%	d.r. (syn:anti)	2.1:1
BDQ (3)	11%	e.r. (3:ent-3)	1.1:1
ent-3	10%	ee	6%

GROUP C

From D or L-Isoleucine, (S),(S) primary amine + n-BuLi

Assay Yield*		Stereoselectivity	
syn + anti	54%	d.r. (syn:anti)	2.2:1
BDQ (3)	17%	e.r. (3:ent-3)	1:1.5
ent-3	20%	ee	−22%

14 + n-BuLi

Assay Yield*		Stereoselectivity	
syn + anti	37%	d.r. (syn:anti)	4.3:1
BDQ (3)	18%	e.r. (3:ent-3)	1.5:1
ent-3	12%	ee	20%

GROUP D

Not derived from amino acids

(S) tetrahydrofuran amine + n-BuLi

Assay Yield*		Stereoselectivity	
syn + anti	78%	d.r. (syn:anti)	1.5:1
BDQ (3)	22%	e.r. (3:ent-3)	1:1.2
ent-3	25%	ee	−8%

(R) tetrahydrofuran amine + n-BuLi

Assay Yield*		Stereoselectivity	
syn + anti	68%	d.r. (syn:anti)	1.5:1
BDQ (3)	22%	e.r. (3:ent-3)	1.2:1
ent-3	19%	ee	8%

From D-Proline

23 + n-BuLi

Assay Yield*		Stereoselectivity	
syn + anti	43%	d.r. (syn:anti)	1.7:1
BDQ (3)	13%	e.r. (3:ent-3)	1:1.1
ent-3	14%	ee	−5%

11 + n-BuLi

Assay Yield*		Stereoselectivity	
syn + anti	48%	d.r. (syn:anti)	3.8:1
BDQ (3)	23%	e.r. (3:ent-3)	1.5:1
ent-3	15%	ee	21%

** Assay yield based on qNMR of the crude reaction mixture obtained after quenching. Triphenylmethane was used as the internal standard*

Scheme S10. *Use of chiral amines derived from amino acids in combination with lithium pyrrolidide for the synthesis of BDQ (**3**)*

Screening of chiral lithium amides similar to pyrrolidine, *N*-methylpiperazine, and morpholine

Although this work was primarily focused on amines derived from D/L-amino acids (morpholine substructure N-C-C-O), other chiral lithium amide bases like pyrrolidine (C-N-C), *N*-methylpiperazine (N-C-C-N), and morpholine were also attempted (Group A, B, and C, respectively) (Scheme S11). For all the molecules studied, no enantioinduction was observed, and the overall yields were low, indicating the generated lithium amides were not basic enough to promote quinoline **1** deprotonation. The majority of starting materials **1** and **2** were recovered after the reaction quench. All the screened molecules shown below are commercially available.

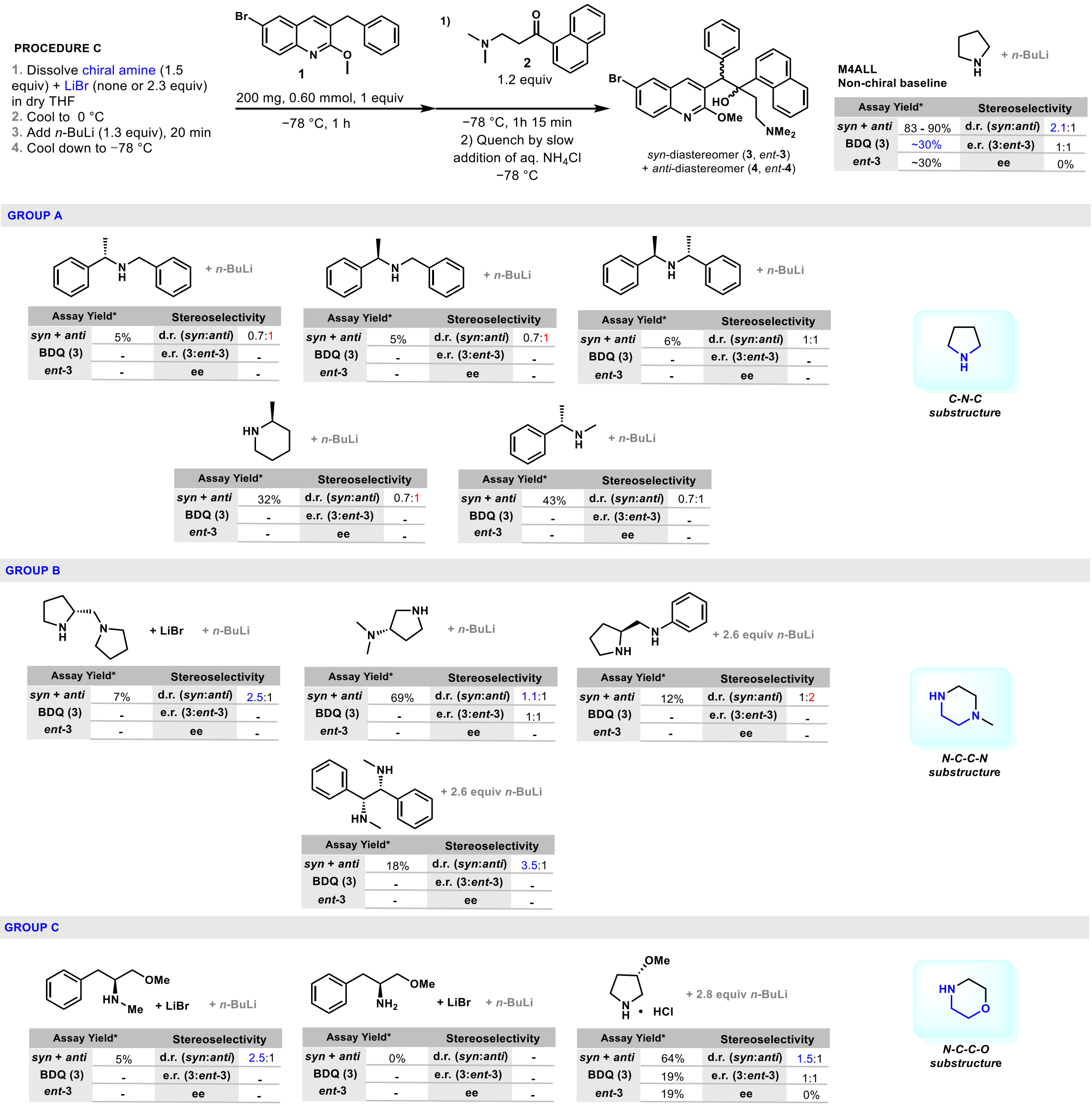

M4ALL – Non-chiral baseline (pyrrolidine + *n*-BuLi)

Assay Yield*		Stereoselectivity	
syn + anti	83 - 90%	d.r. (*syn*:*anti*)	2.1:1
BDQ (3)	~30%	e.r. (3:*ent*-3)	1:1
ent-3	~30%	ee	0%

GROUP A

Assay Yield*		Stereoselectivity	
syn + anti	5%	d.r. (*syn*:*anti*)	0.7:1
BDQ (3)	-	e.r. (3:*ent*-3)	-
ent-3	-	ee	-

Assay Yield*		Stereoselectivity	
syn + anti	5%	d.r. (*syn*:*anti*)	0.7:1
BDQ (3)	-	e.r. (3:*ent*-3)	-
ent-3	-	ee	-

Assay Yield*		Stereoselectivity	
syn + anti	6%	d.r. (*syn*:*anti*)	1:1
BDQ (3)	-	e.r. (3:*ent*-3)	-
ent-3	-	ee	-

Assay Yield*		Stereoselectivity	
syn + anti	32%	d.r. (*syn*:*anti*)	0.7:1
BDQ (3)	-	e.r. (3:*ent*-3)	-
ent-3	-	ee	-

Assay Yield*		Stereoselectivity	
syn + anti	43%	d.r. (*syn*:*anti*)	0.7:1
BDQ (3)	-	e.r. (3:*ent*-3)	-
ent-3	-	ee	-

GROUP B

Assay Yield*		Stereoselectivity	
syn + anti	7%	d.r. (*syn*:*anti*)	2.5:1
BDQ (3)	-	e.r. (3:*ent*-3)	-
ent-3	-	ee	-

Assay Yield*		Stereoselectivity	
syn + anti	69%	d.r. (*syn*:*anti*)	1.1:1
BDQ (3)	-	e.r. (3:*ent*-3)	1:1
ent-3	-	ee	-

Assay Yield*		Stereoselectivity	
syn + anti	12%	d.r. (*syn*:*anti*)	1:2
BDQ (3)	-	e.r. (3:*ent*-3)	-
ent-3	-	ee	-

Assay Yield*		Stereoselectivity	
syn + anti	18%	d.r. (*syn*:*anti*)	3.5:1
BDQ (3)	-	e.r. (3:*ent*-3)	-
ent-3	-	ee	-

GROUP C

Assay Yield*		Stereoselectivity	
syn + anti	5%	d.r. (*syn*:*anti*)	2.5:1
BDQ (3)	-	e.r. (3:*ent*-3)	-
ent-3	-	ee	-

Assay Yield*		Stereoselectivity	
syn + anti	0%	d.r. (*syn*:*anti*)	-
BDQ (3)	-	e.r. (3:*ent*-3)	-
ent-3	-	ee	-

Assay Yield*		Stereoselectivity	
syn + anti	64%	d.r. (*syn*:*anti*)	1.5:1
BDQ (3)	19%	e.r. (3:*ent*-3)	1:1
ent-3	19%	ee	0%

** Assay yield based on qNMR of the crude reaction mixture obtained after quenching. Triphenylmethane was used as the internal standard*

Scheme S11. *Screening of chiral lithium bases with structure alike pyrrolidine, N-Methylpiperazine, and morpholine*

BDQ (3) SYNTHESIS OPTIMIZATION USING LITHIUM (*R*)-2-(METHOXYMETHYL) PYRROLIDIDE (11)

Effect of concentration on the reaction outcome

The data displayed on the Tables S3 and S4 correspond to reactions performed in THF at −78 °C using the chiral lithium amide **11**. Only the impurity with the highest HPLC A% for each case was reported. The main impurity observed comes from ketone **2** (1,4-Michael addition of (*R*)-(methoxymethyl)pyrrolidide (**11**) to the enone **19**) (Scheme S12). Impurity **20** was always observed regardless of the reaction concentration. Notably, when quinoline **1** solution was further concentrated (Entry 3, Table S4), desbromination was favored, and higher percentage of side products **17** and **18** were observed (Scheme S12).

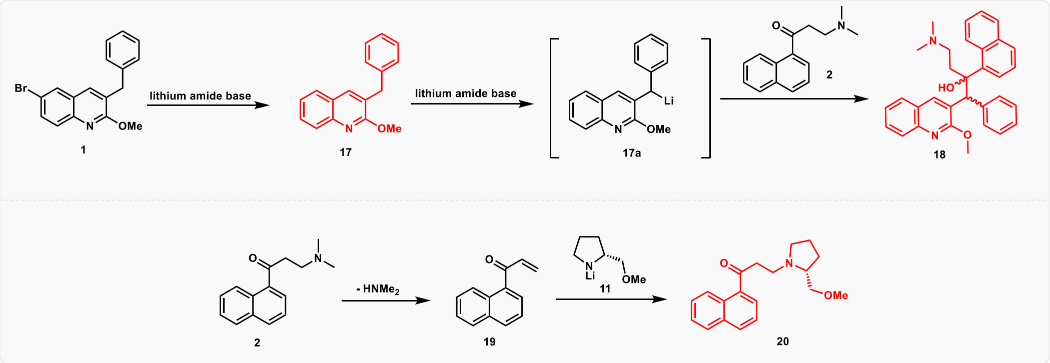

Scheme S12. *Side products formed during BDQ (**3**) synthesis*

Table S3. *Reaction dilution with 15 or 30 V of THF at −78 °C (HPLC A% at 245 nm after reaction quench)*

Entry	Solvent	Input of **1** (g)	*syn*-diast. (**3**, *ent*-**3**)	*anti*-diast. (**4**, *ent*-**4**)	Quinoline **1**	Ketone **2**	Impurity **17**	Impurity **18**	Impurity **20**
1	15 V	0.5	57.1	17.1	6.42	7.85	2.97	1.60	1.30
2	30 V	0.5	72.9	8.83	7.00	2.65	0.08	0	5.30

Experimental Procedure D was used for all the experiments displayed in the table.

Table S4. *Effect of dilution during BDQ (**3**) synthesis in THF at −78 °C (HPLC A% at 245 nm after reaction quench)*

Entry	Solvent vol. R_2NLi^*	Solvent vol. quinoline **1**	Solvent vol. ketone **2**	*syn*-diast. (**3**, *ent*-**3**)	*anti*-diast. (**4**, *ent*-**4**)	Major Impurity	Quinoline **1**	Ketone **2**
1	5 V	5 V	5 V	50.0	8.00	7.90 (**20**)	5.50	5.10
2	10 V	5 V	5 V	68.4	8.40	4.00 (**20**)	6.30	3.60
3	10 V	3 V	7 V	36.1	11.6	17.7 (**17**)**	11.6	4.20
4	5 V	5 V	10 V	60.0	12.0	4.50 (**20**)	13.1	8.50
5	10 V	5 V	10 V	53.6	10.4	4.00 (**20**)	18.5	3.10
6	10 V	10 V	10 V	71.0	9.55	8.50 (**20**)	3.00	2.20

*$^*R_2NLi$ = Lithium (R)-(methoxymethyl)pyrrolidide **11** **Only case where major impurity was not compound **20**. Quinoline **17** was detected as the main side product. Experimental Procedure D was used for all the experiments displayed in the table.*

Table S5. *Reaction dilution with 15 or 20 V of 2-MeTHF at −40 °C (HPLC A% at 245 nm after reaction quench)*

Entry	Input of **1** (g)	Solvent	*syn*-diast. (**3**, *ent*-**3**)	*anti*-diast. (**4**, *ent*-**4**)	Quinoline **1**	Ketone **2**	Impurity **19**	Impurity **20**	Impurity **22**
1	5.0	15 V	59.5	4.18	13.9	9.52	0.16	0	5.50
2	25.0	20 V	74.5	5.71	6.57	6.84	0.12	0	3.60
3	75.0	20 V	77.7	5.70	5.18	7.11	0	0	2.87

Experimental Procedure D was used for all the experiments displayed in the table.

Assessing the quality of (*R*)-2-(methoxymethyl)pyrrolidine (11)

D-prolinol purchased from three vendors in order to synthetize the (*R*)-2-(methoxymethyl)pyrrolidine **11**. Table S6 displays data of each batch after distillation of desired product.

Table S6. *Comparison of chiral amine obtained from different vendors*

Entry	Vendor	Vendor	Input (g)	Output (g)	SOR	Purity by GCHS (A%)	Chiral Purity (A%)
1	A	BLD Pharma	250.0	60	−8.806°	99.89	99.5
2	B	ASUN	250.0	58	−8.292°	99.2	99.9
3	C	Combi-Blocks	50.0	11	−8.674°	99.53	100

SOR: Specific optical rotation; solvent $CHCl_3$, concentration ~1.0 g/ 100 cm^3; GCHS: Headspace gas chromatography.

HIGH-PERFORMANCE LIQUID CHROMATOGRAPHY (HPLC) METHODS

Analysis and reaction monitoring

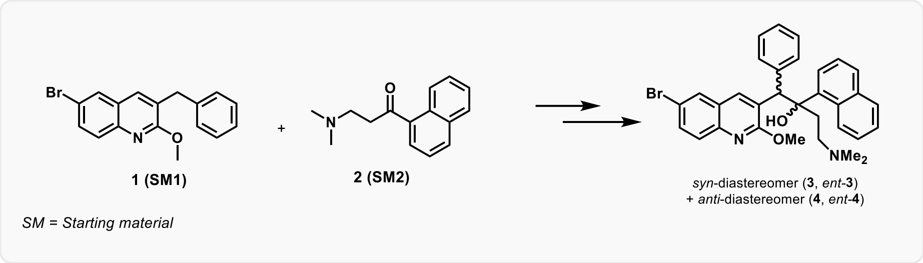

Conditions:

Column: Eclipse Plus C18 (4.6 x 100 mm; 3.5 µM particles)

Mobile Phase A: 55:45 Acetonitrile:Aqueous 15 mM NaH_2PO_4 (not adjusted) - premixed

Mobile Phase B: 95:5 Acetonitrile:Water

Injection volume: 1 µL

Column temp: 30 °C

Flow rate: 1.5 mL/min

Detector wavelength(s): 220 nm for assay, 245 nm for reaction monitoring

Table S7: *Liquid Chromatography gradient of mobile phases A and B*

Time (min)	% A	% B
0.0	100	0
5.0	100	0
10.0	0	100
15.0	0	100

Post-run equilibration: 5 min

Notes: The use of the higher pH (~4.6) aqueous mobile phase was required to give acceptable peak shape for the *syn* and *anti*-diastereomers. Buffer solubility was an issue leading to the use of the sodium salt rather than the potassium salt. Using water in B rather than buffer helped avoid this concern without sacrificing performance. Premixing A and B simplified the complexity of the gradient and any instrumental issues that might cause. It was very difficult to increase retention of ketone **2** and maintain acceptable peak shape. Sample preparation can be difficult due to poor solubility of the mixture of BDQ stereoisomers (especially the *anti*-diastereomers pair, **4** and *ent*-**4**). We found that a 1 mg/mL solution in acetonitrile was possible

with mild heat and sonication but use 0.5 mg/mL instead due to concerns about precipitation in autosampler vials. For assay of BDQ, it is possible to just run the isocratic portion of the method.

Table S8. *Retention times and relative response factor (RRF) of the main reaction components*

Compound	Time (min)	RRF (mg/mL) at 245 nm	RRF (M) at 245 nm
syn-diastereomer pair (**3** and *ent*-**3**)	5.9	1.00	1.00
anti-diastereomer pair (**4** and *ent*-**4**)	4.3	0.91	0.91
Quinoline **1**	10.6	1.05	0.62
Ketone **2**	0.6	0.81	0.33
Compound **17**	8.7	0.73	0.33

Representative Chromatogram:

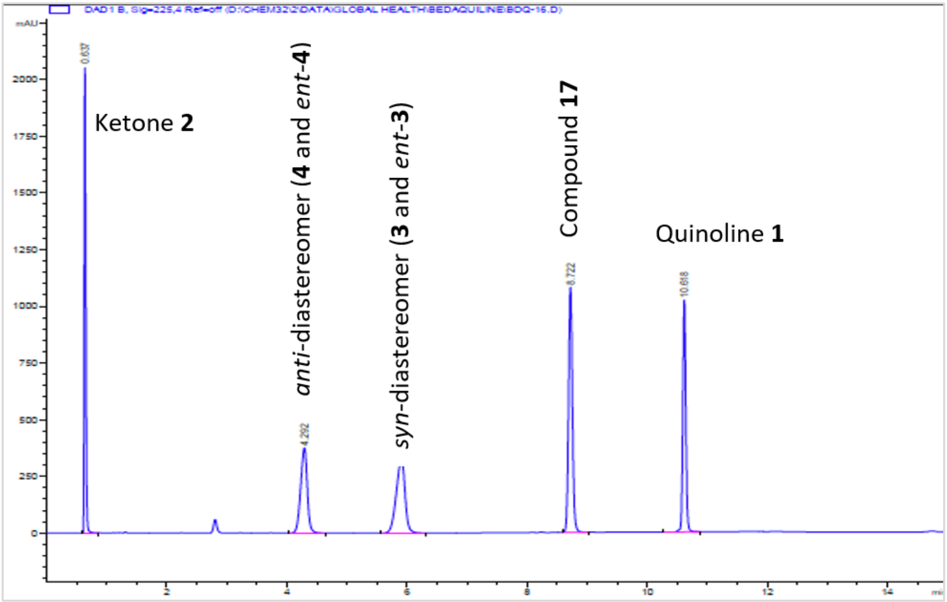

Figure S16. *HPLC profile showing the retention times of different components observed in the reaction mixture (non-asymmetric approach)*

BDQ Diastereomer Spectra　　　　　Quinoline **1** Spectra

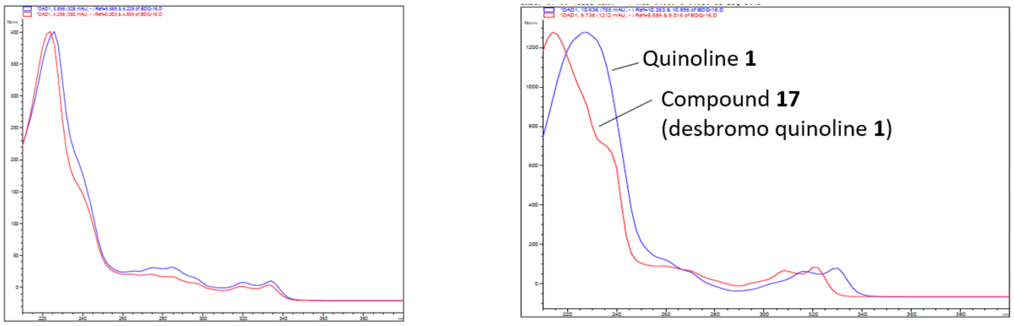

Figure S17. *UV spectra syn-diastereomer pair (**3** and ent-**3**), quinoline **1**, and desbromo quinoline **17***

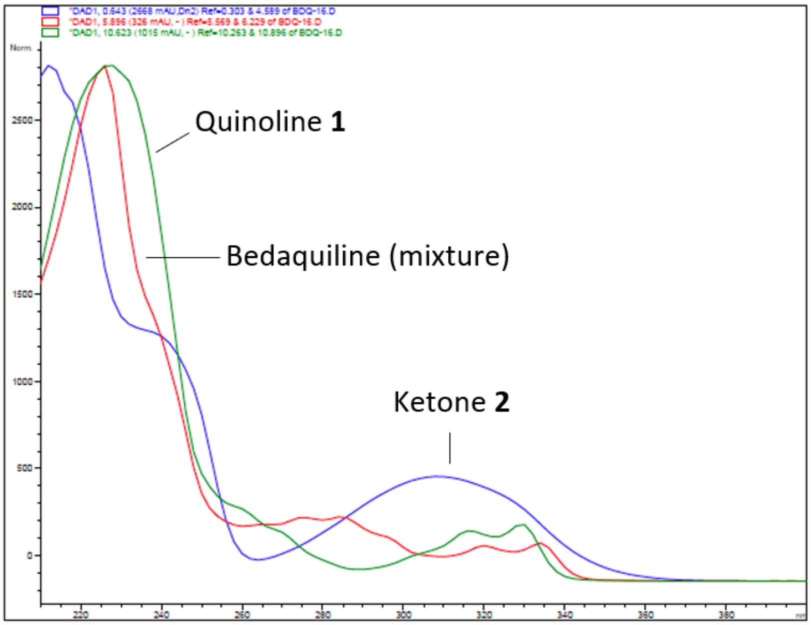

Figure S18. *Overlaid UV spectra of BDQ mixture of four stereoisomers, quinoline **1**, and ketone **2***

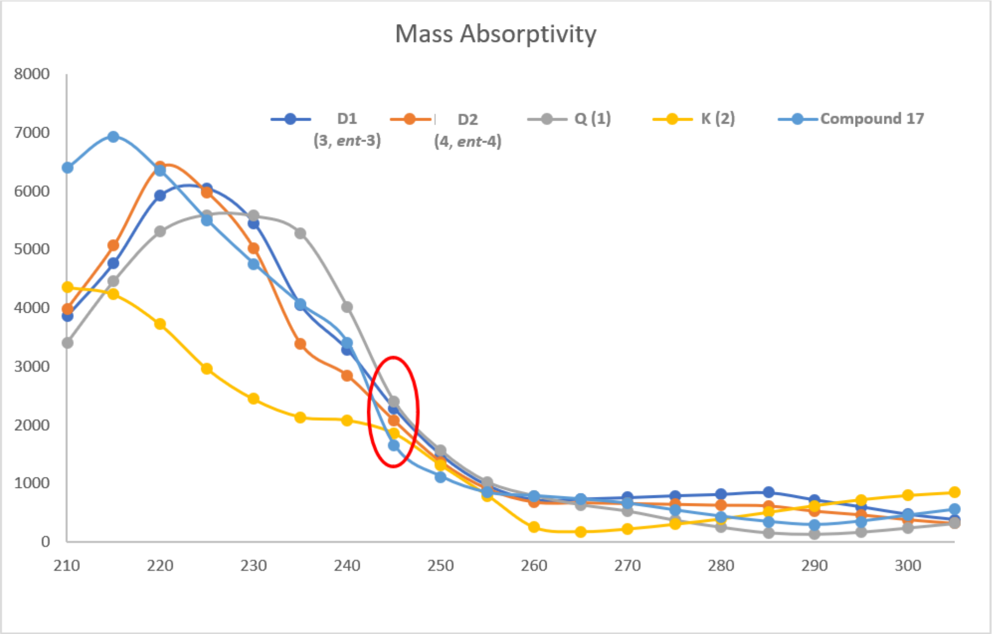

Figure S19. *Isosbestic plot based on mass – syn and anti-diastereomers (**3**+ent-**3**+**4**+ent-**4**), quinoline **1**, ketone **2**, and desbromo quinoline **17***

Analysis of BDQ (3) and stereoisomers via Supercritical Fluid Chromatography (SFC)

Conditions:

Column: ChiralPak IC-3, (4.6 mm ID X 250 mm L, 3 µM)

Mobile Phase: 25% Ethanol (1.0% DEA) against CO_2

Back Pressure: 100 bar at 60 °C

Flow rate: 2.5 mL/min

Injection volume: 10 µL

Column temp: 25 °C

Detector wavelength: 260 nm

Sample preparation: Prepare samples at approximately 1.0 mg/mL in methanol

Table S9. *Retention times and relative response factor (RRF) of the main reaction components*

Compound	Time (min)
BDQ (target, **3**)	4.36
BDQe (undesired enantiomer *ent*-**3**)	5.78
anti-diastereomer pair (**4** and *ent*-**4**)	6.26 and 11.1

Representative Chromatogram:

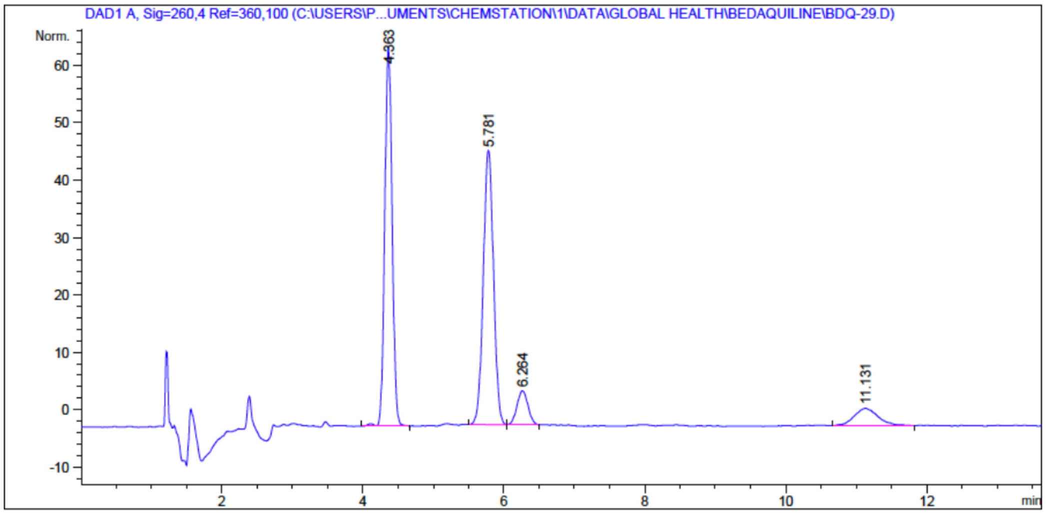

Figure S20. *Chromatogram of mixture of BDQ stereoisomers (**3**+ent-**3**+**4**+ent-**4**)*

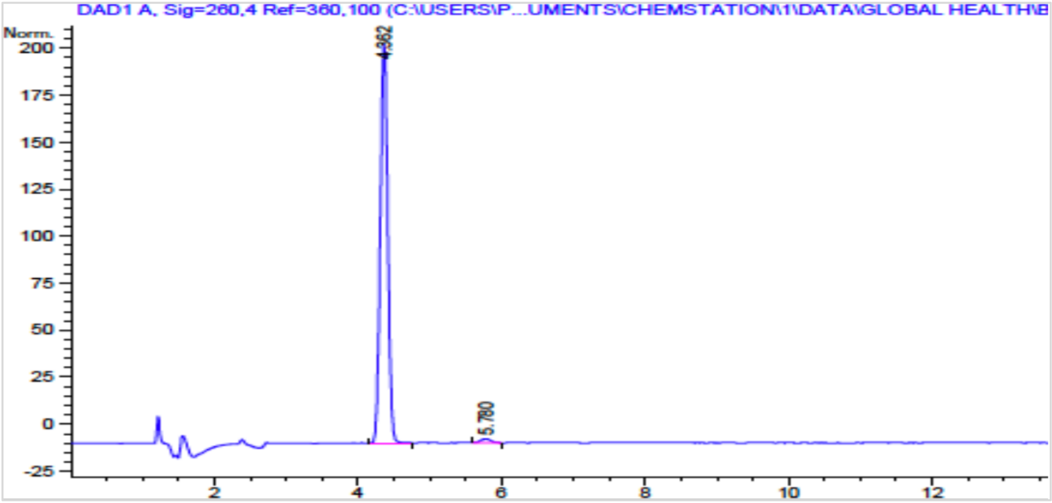

Figure S21. *Chromatogram of target BDQ (**3**)*

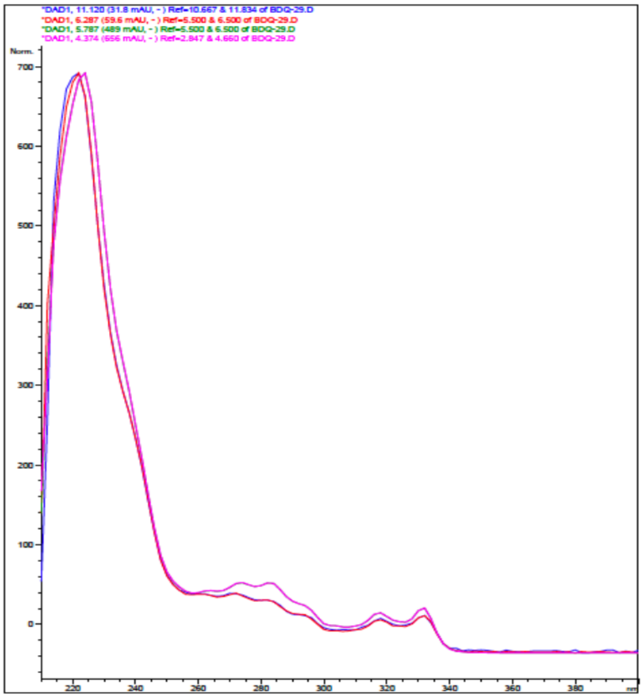

Figure S22. *Overlaid UV spectra of the four BDQ stereoisomers (**3**+ent-**3**+**4**+ent-**4**)*

^{1}H AND ^{13}C NUCLEAR MAGNETIC RESONANCE (NMR) SPECTRA

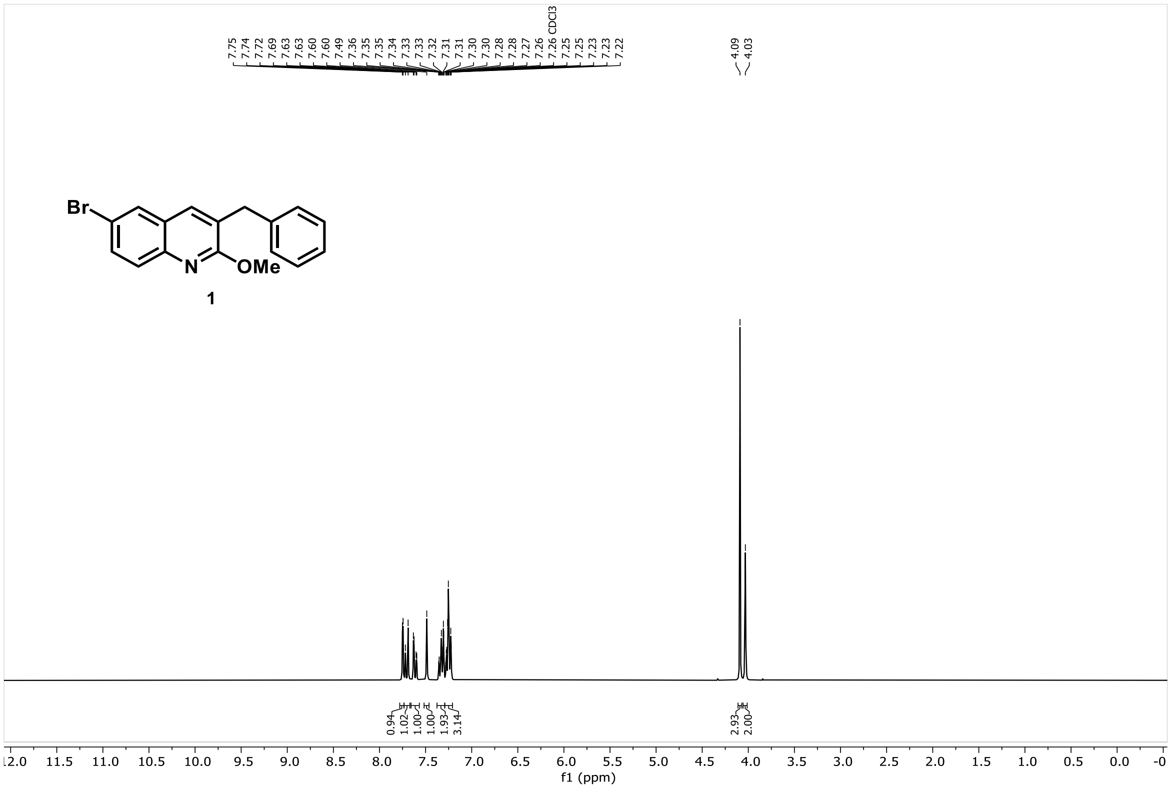
Br
N
OMe
1
f1 (ppm)

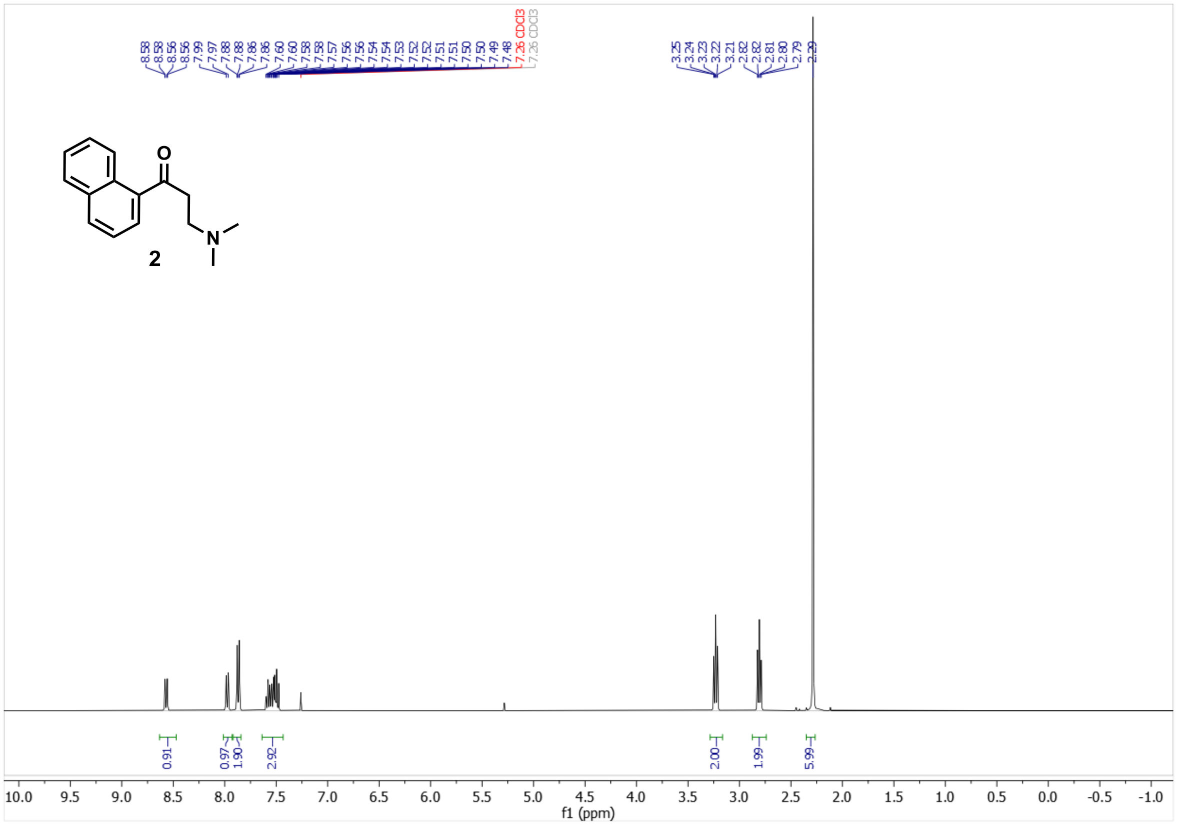
O
N
2
f1 (ppm)

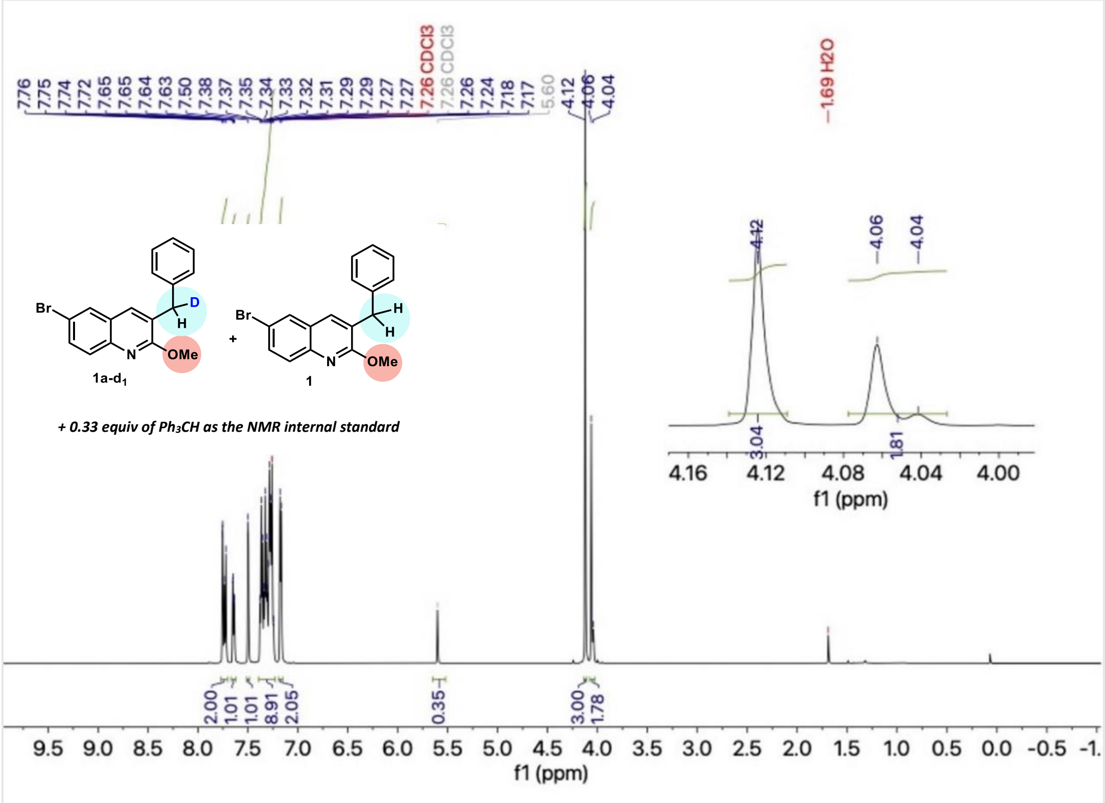
7.76
7.75
7.74
7.72
7.65
7.65
7.64
7.63
7.50
7.38
7.37
7.35
7.34
7.33
7.32
7.31
7.29
7.29
7.27
7.27
7.26 CDCl3
7.26 CDCl3
7.26
7.24
7.18
7.17
5.60
4.12
4.06
4.04
1.69 H2O
Br
D
H
N
OMe
1a-d1
+
Br
H
H
N
OMe
1
+ 0.33 equiv of Ph3CH as the NMR internal standard
4.12
4.06
4.04
3.04
1.81
4.16
4.12
4.08
4.04
4.00
f1 (ppm)
2.00
1.01
1.01
8.91
2.05
0.35
3.00
1.78
9.5
9.0
8.5
8.0
7.5
7.0
6.5
6.0
5.5
5.0
4.5
4.0
3.5
3.0
2.5
2.0
1.5
1.0
0.5
0.0
-0.5
-1.
f1 (ppm)

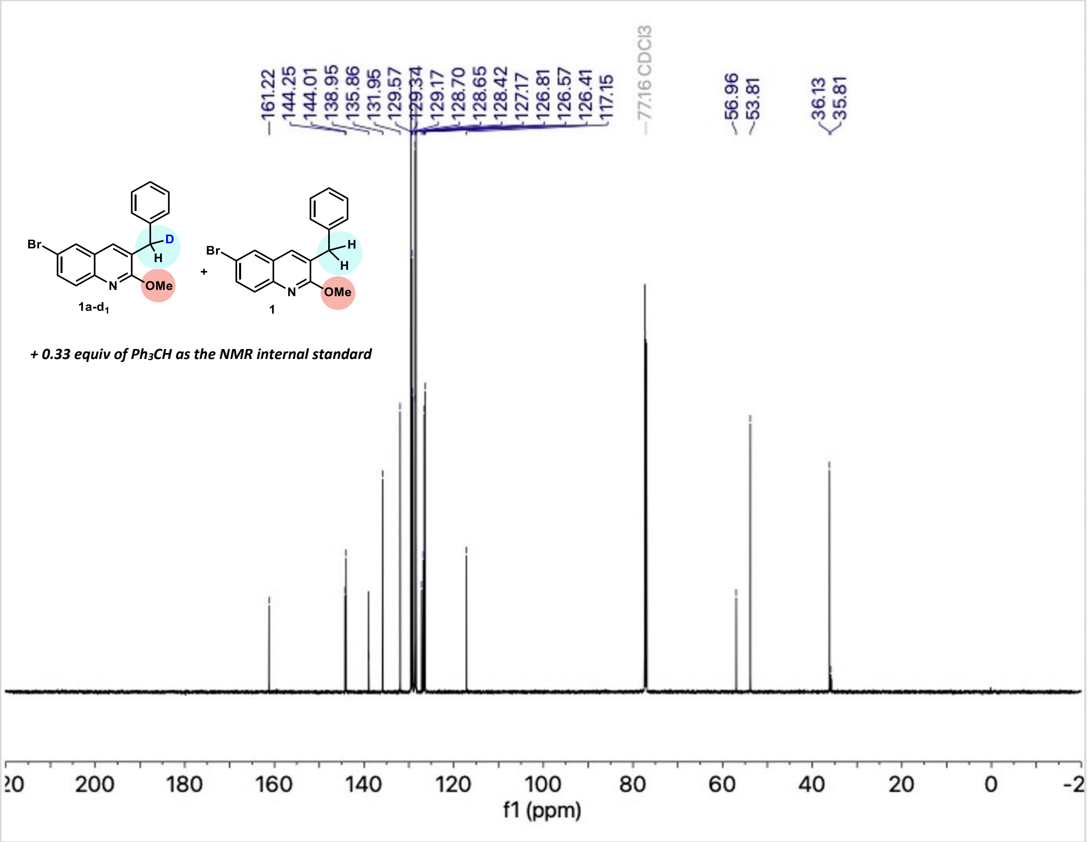
161.22
144.25
144.01
138.95
135.86
131.95
129.57
129.34
129.17
128.70
128.65
128.42
127.17
126.81
126.57
126.41
117.15
77.16 CDCl3
56.96
53.81
36.13
35.81
Br
D
H
N
OMe
1a-d1
+
Br
H
H
N
OMe
1
+ 0.33 equiv of Ph3CH as the NMR internal standard
20
200
180
160
140
120
100
80
60
40
20
0
-2
f1 (ppm)

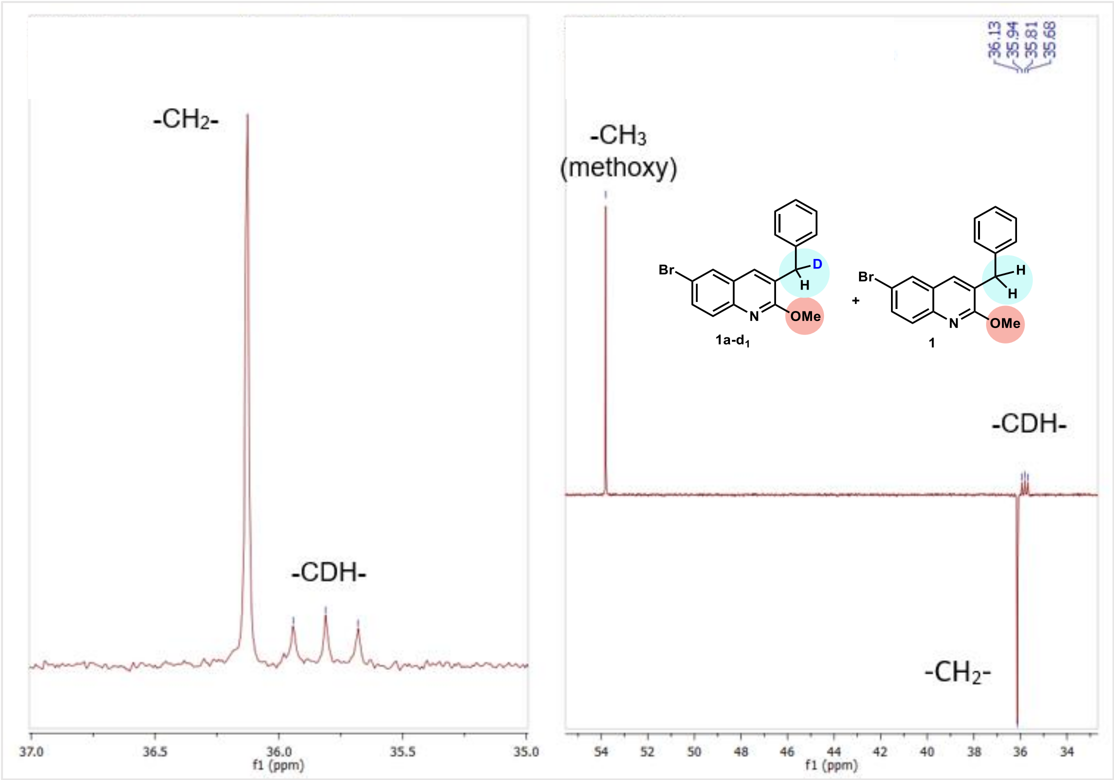
-CH2-
-CDH-
f1 (ppm)
36.13
35.94
35.81
35.68
-CH3
(methoxy)
Br
D
H
N
OMe
1a-d1
+
1
-CDH-
-CH2-
f1 (ppm)

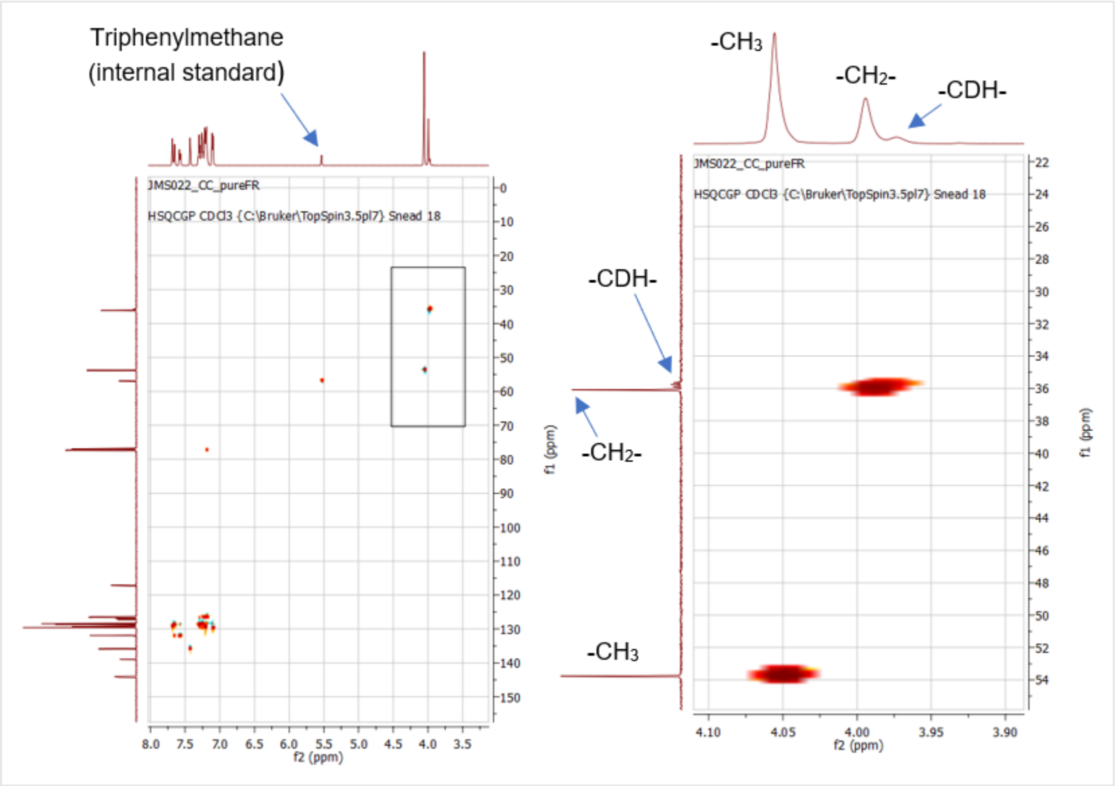
Triphenylmethane
(internal standard)
JMS022_CC_pureFR
HSQCGP CDCl3 {C:\Bruker\TopSpin3.5pl7} Snead 18
f2 (ppm)
f1 (ppm)
-CH3
-CH2-
-CDH-
-CDH-
-CH2-
-CH3
JMS022_CC_pureFR
HSQCGP CDCl3 {C:\Bruker\TopSpin3.5pl7} Snead 18
f2 (ppm)
f1 (ppm)

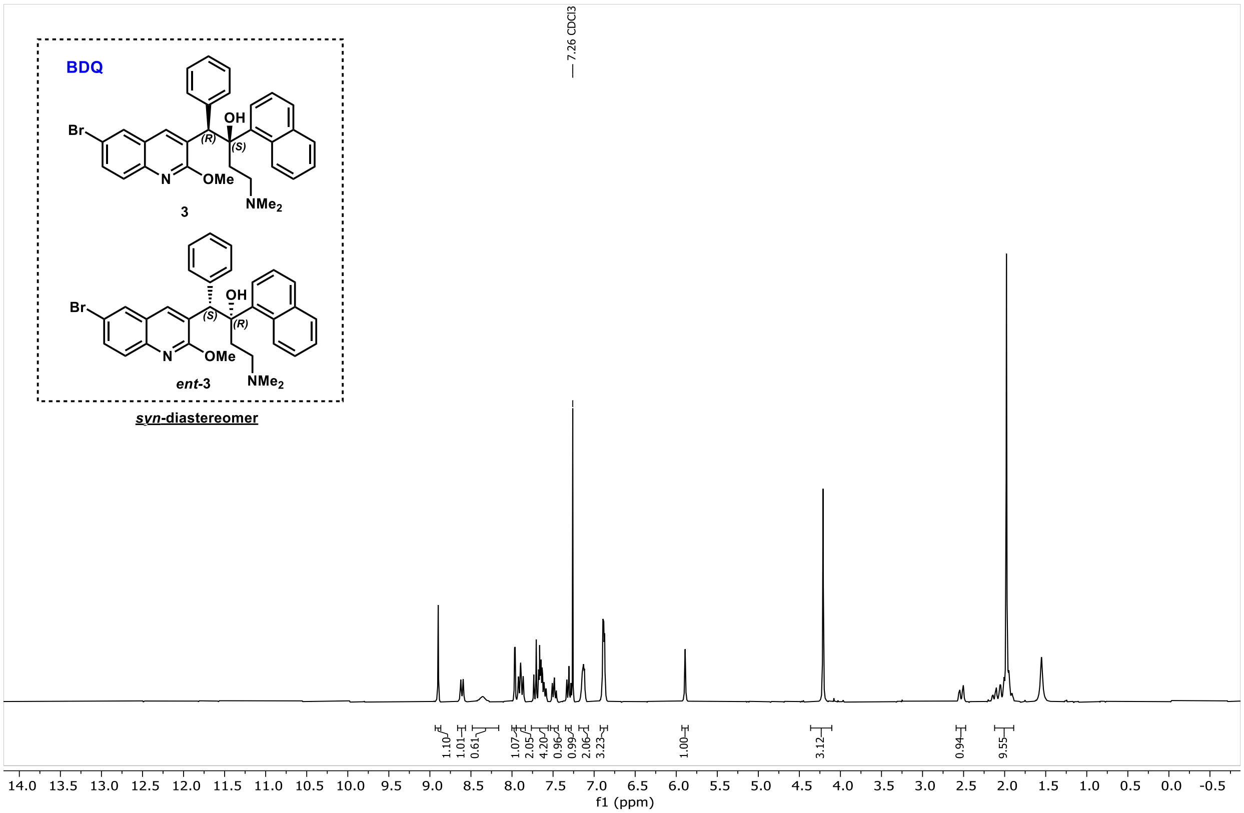
BDQ
Br
N
OMe
OH
(R)
(S)
NMe$_2$
3
ent-3
syn-diastereomer
7.26 CDCl3
1.10
1.01
0.61
1.07
2.05
4.20
0.96
0.99
2.06
3.23
1.00
3.12
0.94
9.55
f1 (ppm)

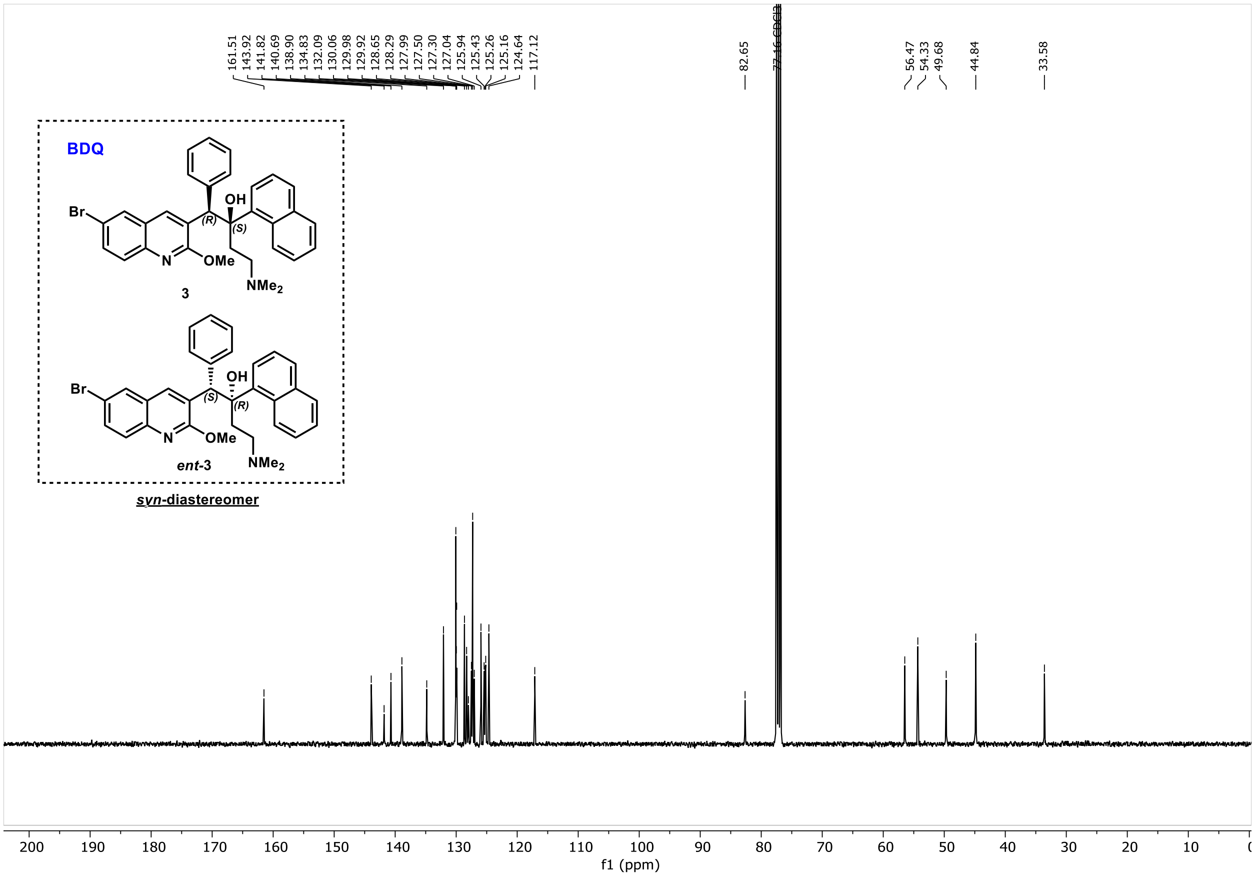
BDQ
Br
N
OMe
OH
(R)
(S)
NMe$_2$
3
ent-3
syn-diastereomer
161.51
143.92
141.82
140.69
138.90
134.83
132.09
130.06
129.98
129.92
128.65
128.29
127.99
127.50
127.30
127.04
125.94
125.43
125.26
125.16
124.64
117.12
82.65
77.16 CDCl3
56.47
54.33
49.68
44.84
33.58
f1 (ppm)

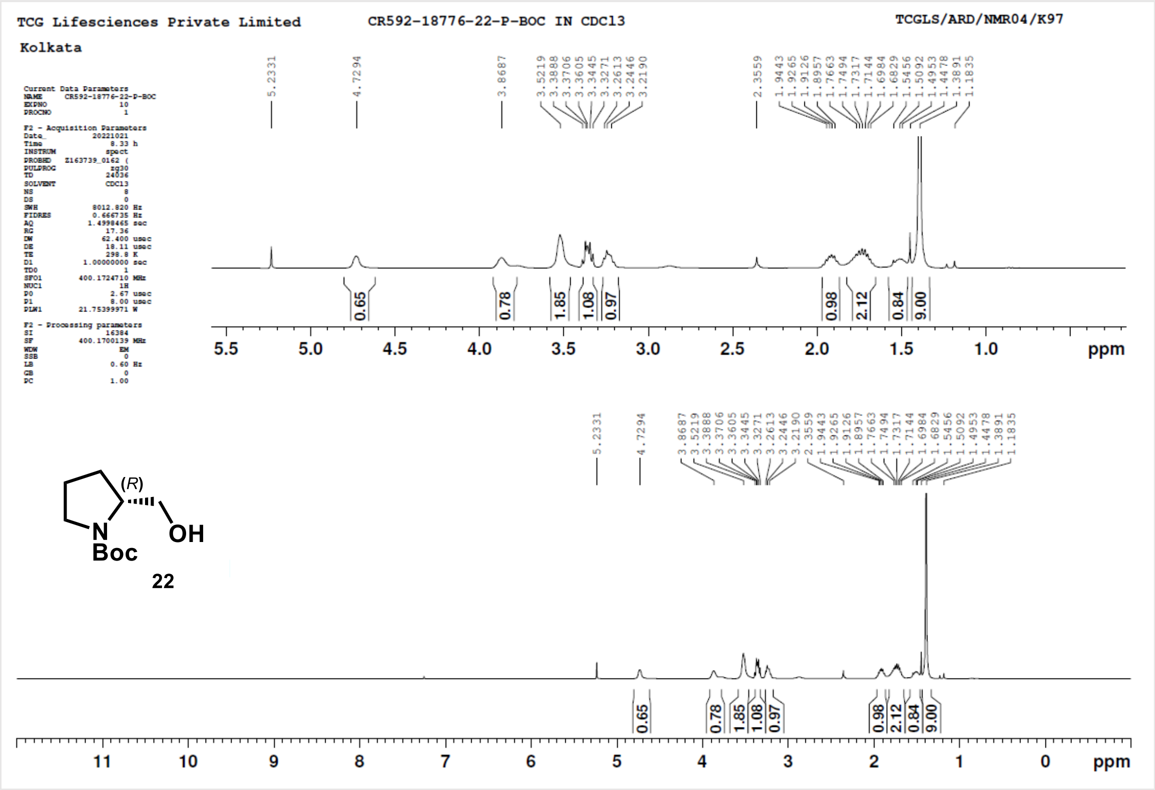
TCG Lifesciences Private Limited
Kolkata
CR592-18776-22-P-BOC IN CDCl3
TCGLS/ARD/NMR04/K97
(R)
N
OH
Boc
22
ppm

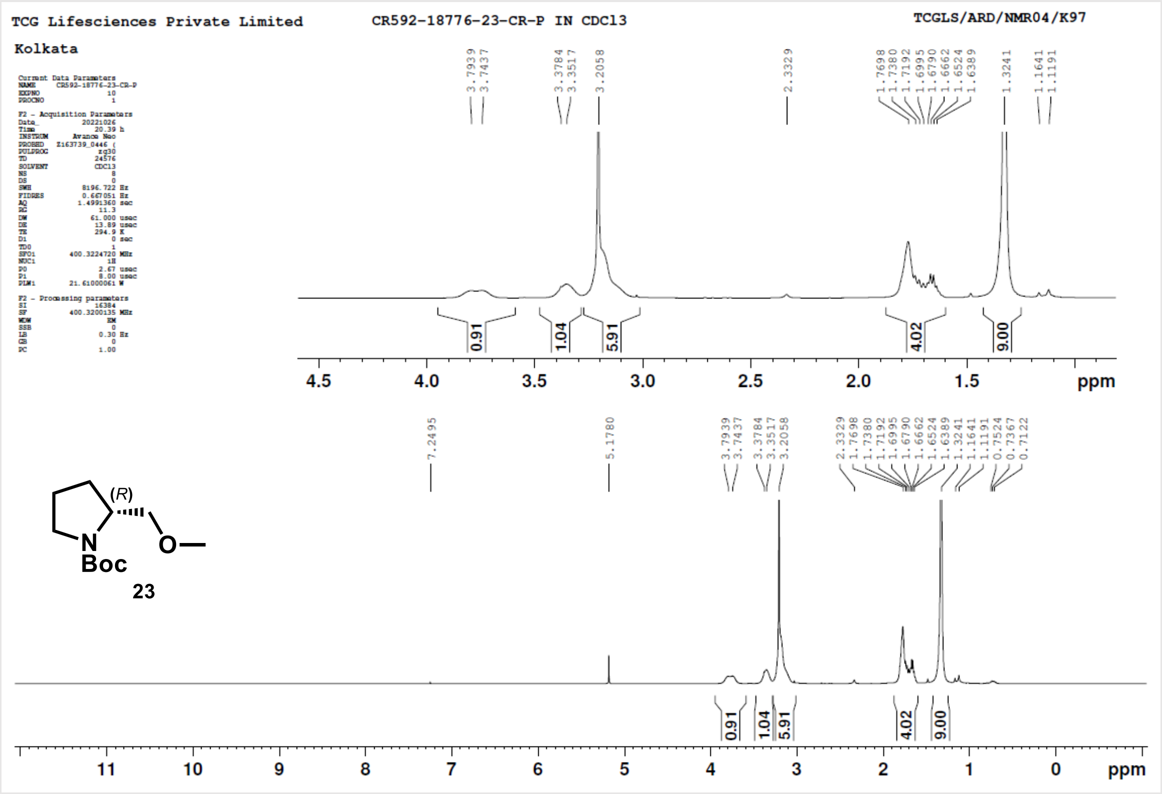
TCG Lifesciences Private Limited
Kolkata
CR592-18776-23-CR-P IN CDCl3
TCGLS/ARD/NMR04/K97
(R)
N
O
Boc
23
ppm

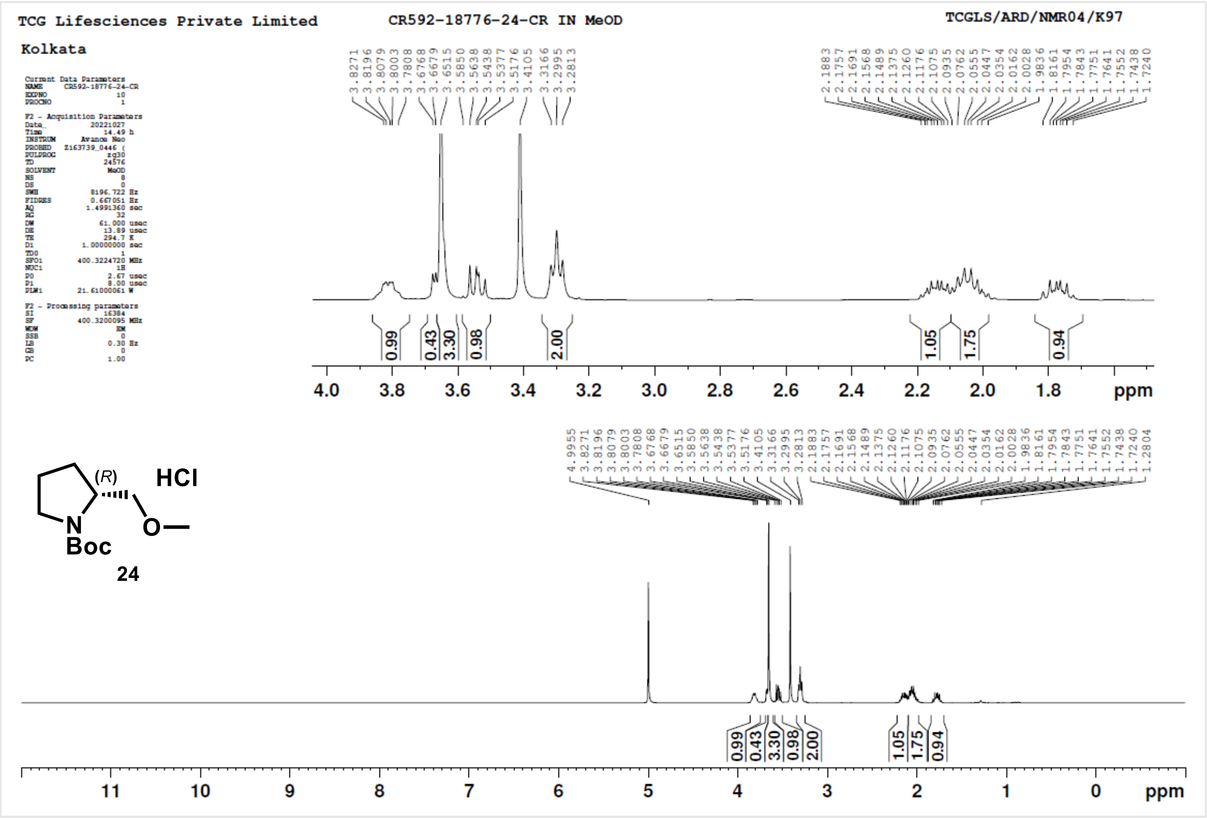
TCG Lifesciences Private Limited
CR592-18776-24-CR IN MeOD
TCGLS/ARD/NMR04/K97
Kolkata
(R)
HCl
N
O
Boc
24
ppm

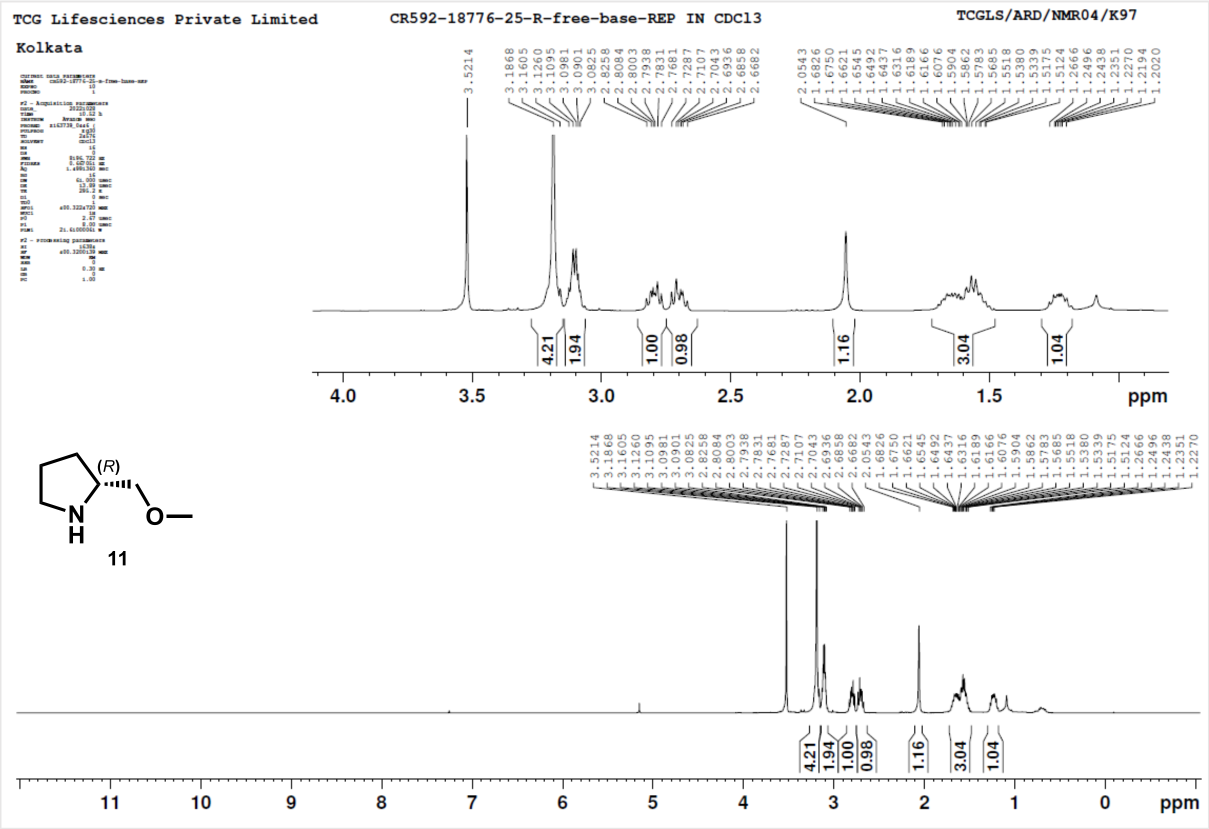
TCG Lifesciences Private Limited
CR592-18776-25-R-free-base-REP IN CDCl3
TCGLS/ARD/NMR04/K97
Kolkata
(R)
N
H
O
11
ppm

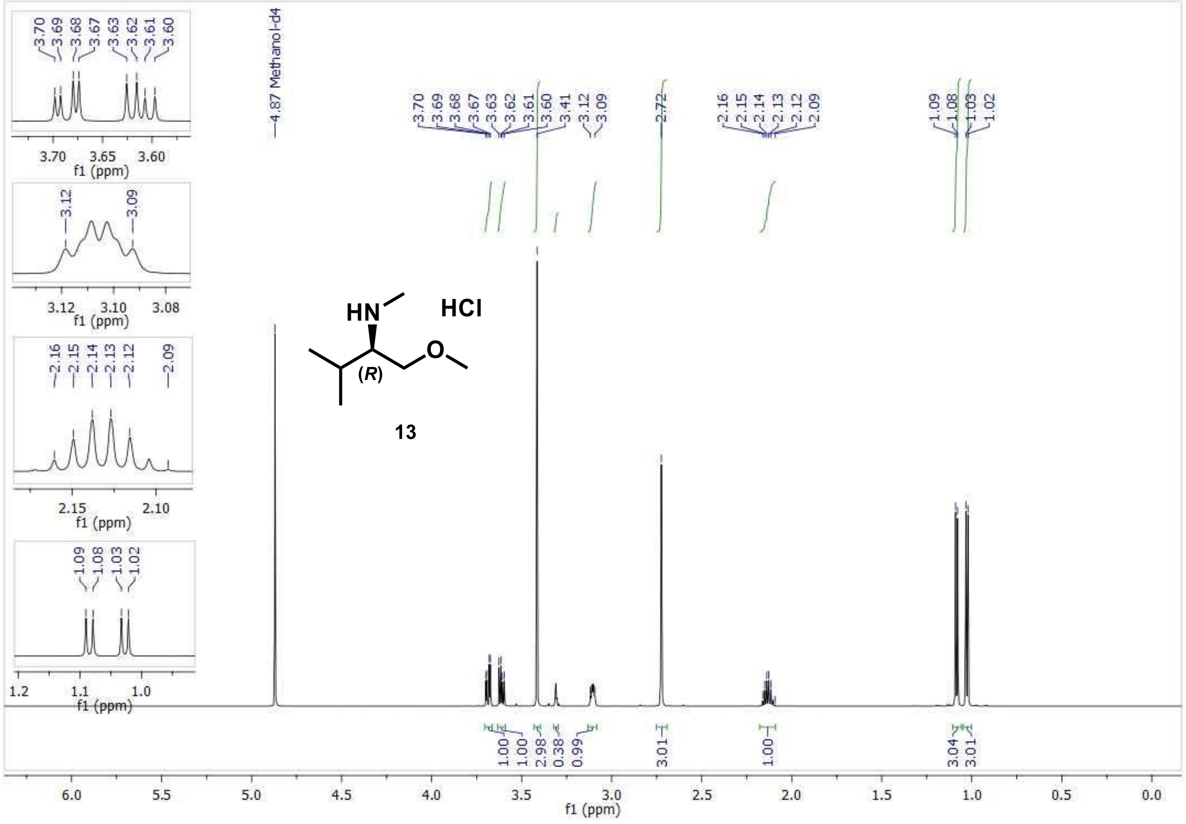
4.87 Methanol-d4
3.70 3.69 3.68 3.67 3.63 3.62 3.61 3.60 3.41 3.12 3.09
2.72
2.16 2.15 2.14 2.13 2.12 2.09
1.09 1.08 1.03 1.02
HN
HCl
O
(R)
13
1.00 1.00 2.98 0.38 0.99 3.01 1.00 3.04 3.01
f1 (ppm)

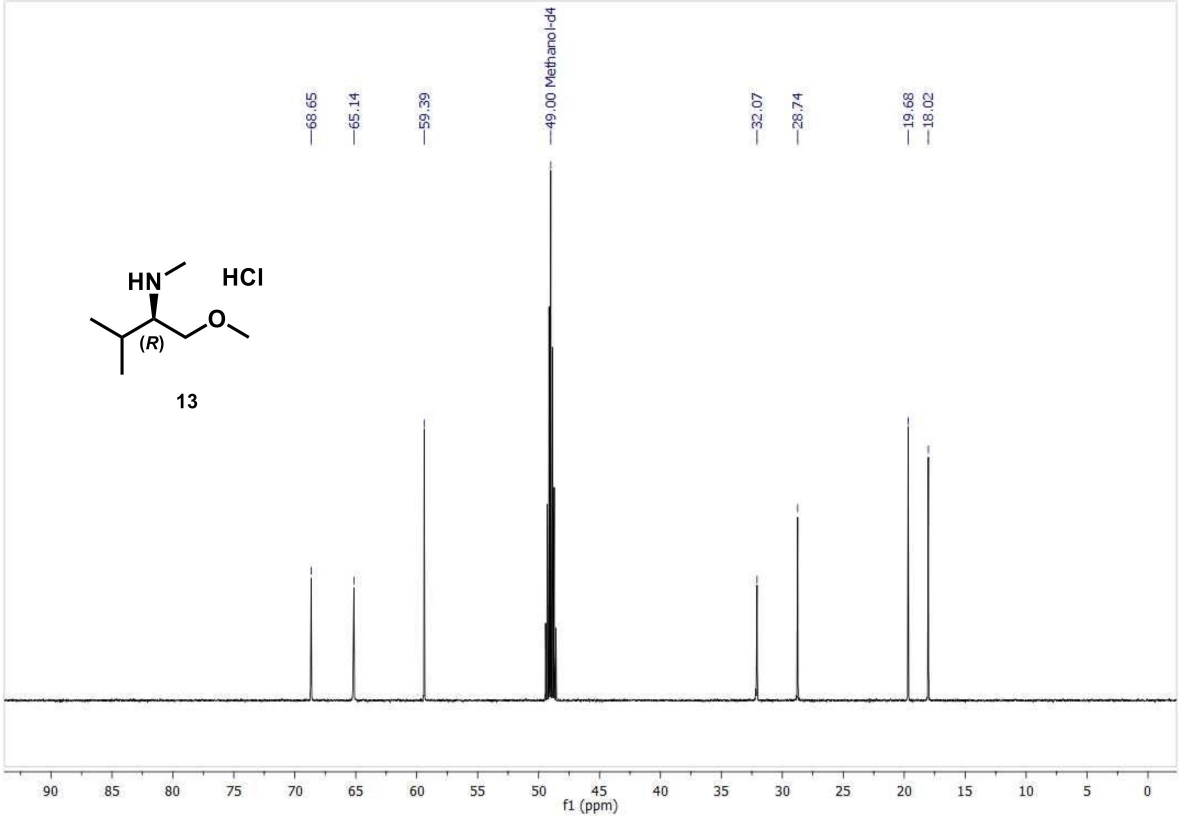
68.65
65.14
59.39
49.00 Methanol-d4
32.07
28.74
19.68
18.02
HN
HCl
O
(R)
13
f1 (ppm)

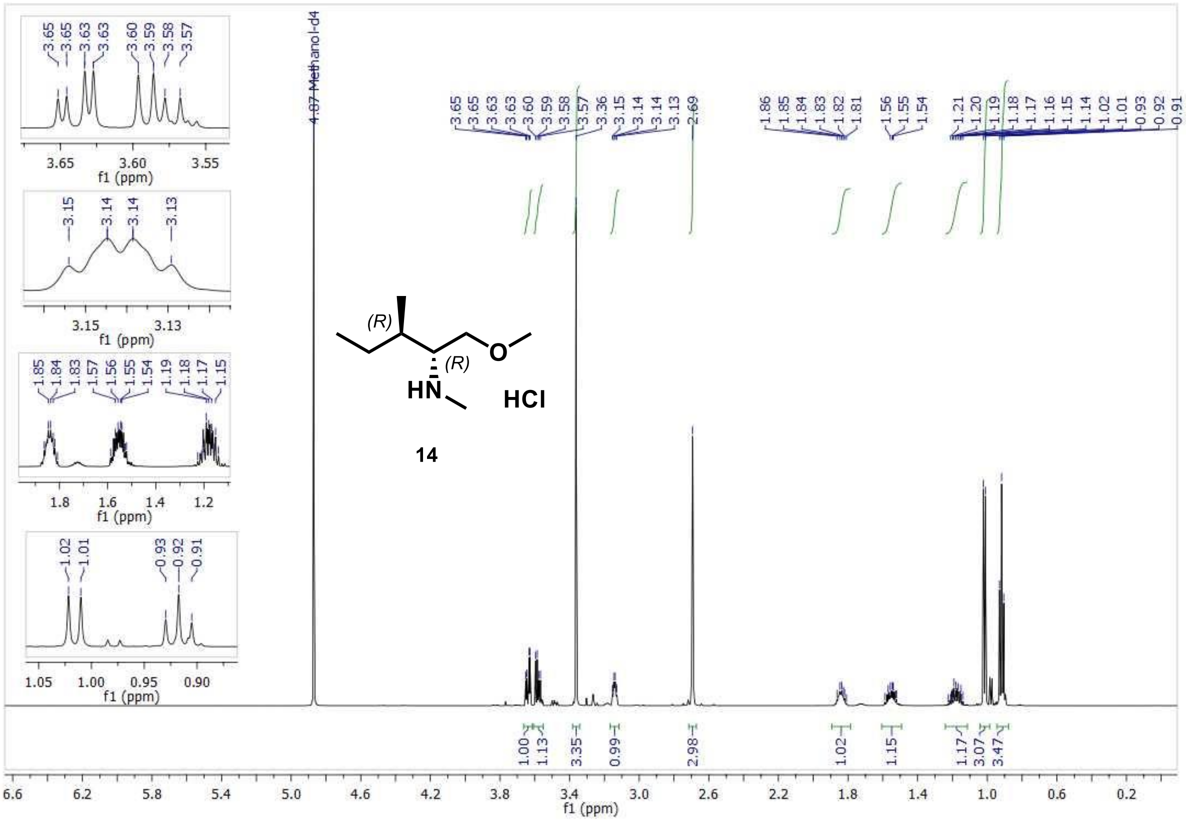
4.87 Methanol-d4
(R)
(R)
HN
HCl
14
f1 (ppm)

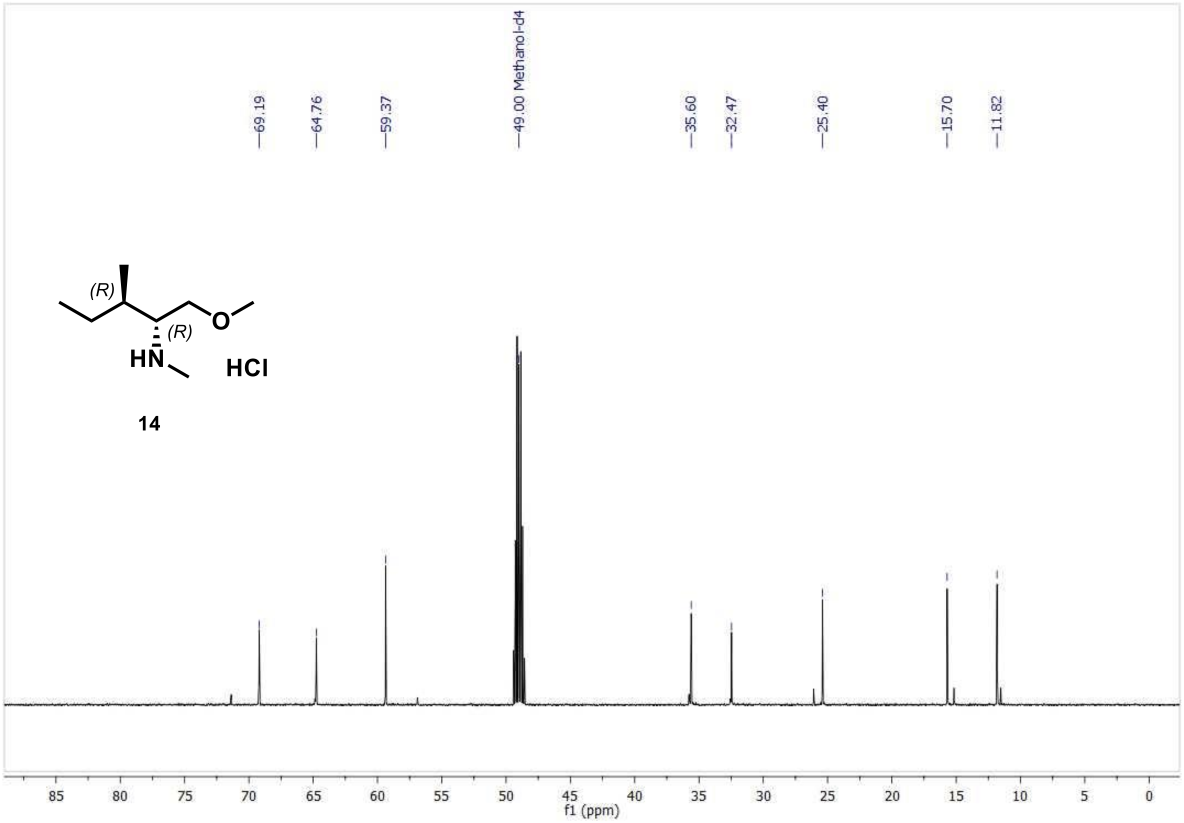
69.19
64.76
59.37
49.00 Methanol-d4
35.60
32.47
25.40
15.70
11.82
(R)
(R)
HN
HCl
14
f1 (ppm)

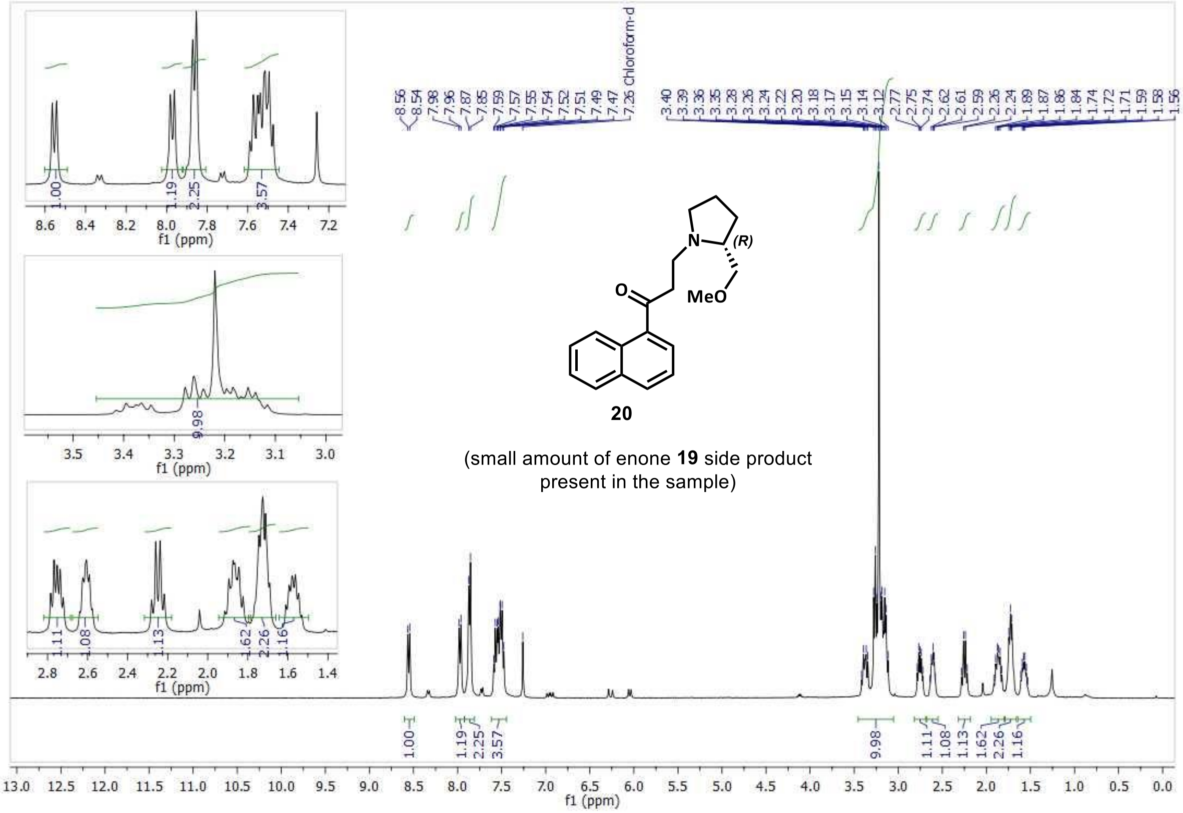
(small amount of enone 19 side product
present in the sample)
20
MeO
(R)
Chloroform-d
f1 (ppm)

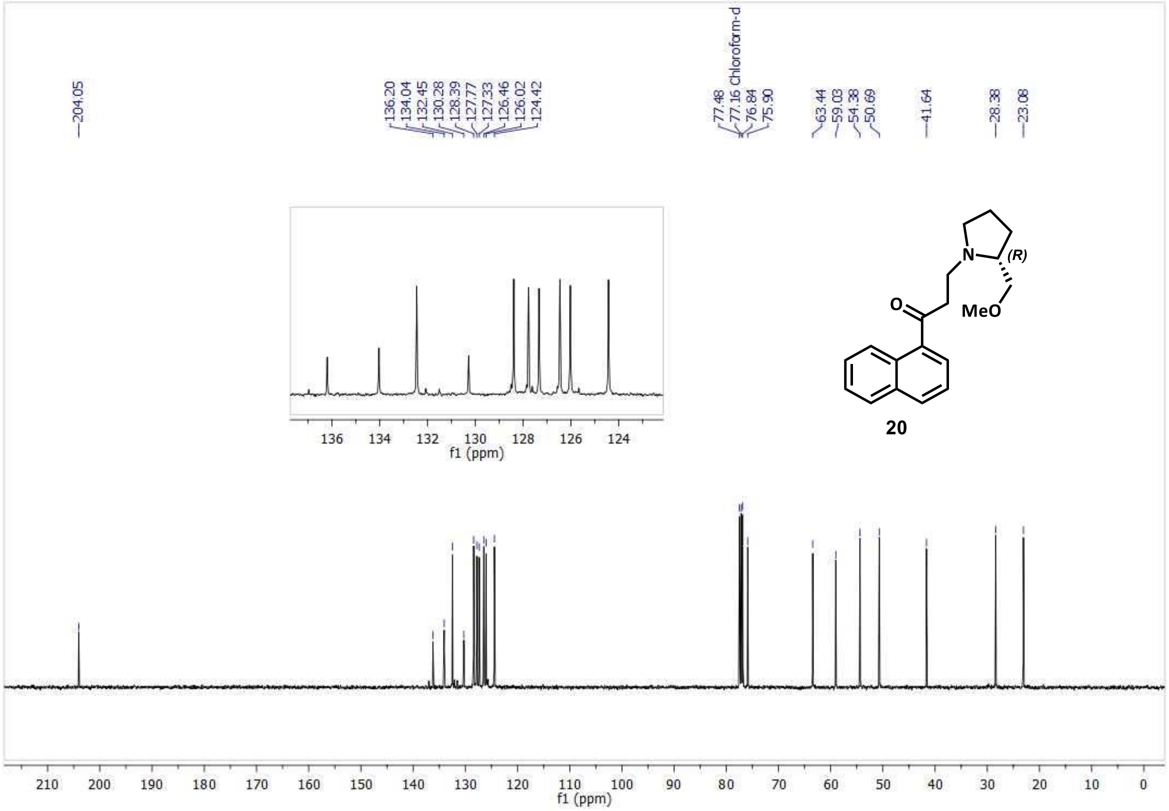
20
MeO
(R)
Chloroform-d
f1 (ppm)

REFERENCES

[1] Burchat, A. F.; Chong, J. M.; Nielsen, N. Titration of alkyllithiums with a simple reagent to a blue endpoint. *J. Organomet. Chem.* **1997**, *542*, 281–283. DOI: 10.1016/S0022-328X(97)00143-5.

[2] (a) Porstmann, F. R.; Horns, S.; Bader, T. Process for preparing (α*S*,β*R*)-6-bromo-α-[2-(dimethylamino)ethyl]-2-methoxy-α-1-naphthalenyl-β-phenyl-3-quinolineethanol. WO 2006/125769 A1, 2006. (b) Hegyi, J. F. A. L.; Aelterman, W. A. A.; Lang, Y. L.; Stokbroekx, S. C. M.; Leys, C.; Remoortere, P. J. M. V.; Faure, A. Fumarate salt of (α*S*, β*R*)-6-bromo-α-[2-(dimethylamino)ethyl]-2-methoxy-α-1-naphthalenyl-β-phenyl-3-quinolineethanol. EP 2 086 940 B1, 2007. (c) Lustig, P.; Stefko, M. Method of Isolation of a Mixture of Enantiomers of 1-(6-bromo-2-methoxyquinolin-3-yl)-4-(dimethylamino)-2-(naphthalen-1-yl)-1-phenylbutan-2-ol. WO 2016/116075 A1, 2016. (d) Xueqing, Z.; Yangwei, H.; Zhiyao, Z.; Yanqin, L.; Zhong, C. Chiral inducers for the synthesis of (1*R*,2*S*)-bedaquiline. CN 106866525 A, 2017. (e) Wenhua, F.; Delong, K. A method of recycling and utilize bedaquiline three-dimensional chemical isomer. CN 105017147 B, 2014. (f) Sebastian, S.; Singh, S. K.; Polavarapu, S.; Veera, U. Process for the preparation of bedaquiline fumarate. WO 2020/161743 A1, 2020. (g) Kim, Y.; Kim, J.; Shin, C. (1*R*,2*S*)-1-(6-bromo-2-methoxyquinolin-3-yl)-4-dimethylamino-2-(1-naphthyl)-1-phenyl-butan-2-ol and their Method for preparing a pharmaceutically acceptable salt. KR 102303635 B1, 2020. (h) Lubanyana, H.; Arvidsson, P. I.; Govender, T.; Kruger, H. G.; Naicker, T. Improved Synthesis and Isolation of Bedaquiline. *ACS Omega* **2020**, *5* (7), 3607–3611. DOI: 10.1021/acsomega.9b04037. (i) Mear, S. J.; Lucas, T.; Ahlqvist, G. P.; Robey, J. M. S.; Dietz, J. -P.; Khairnar, P. V.; Maity, S.; Williams, C. L.; Snead, D. R.; Nelson, R. C.; Opatz, T.; Jamison, T. F. Diastereoselectivity Is in the Details: Minor Changes Yield Major Improvements to the Synthesis of Bedaquiline. *Chem. Eur. J.* **2022**, *28* (47), e2022013. DOI: 10.1002/chem.202201311. (j) Gao, F.; Li, J.; Ahmad, T.; Luo, Y.; Zhang, Z.; Yuan, Q.; Huo, X.; Song, T.; Zhang, W. Asymmetric Synthesis of Bedaquiline Based on Bimetallic Activation and Non-Covalent Interaction Promotion Strategies. *Sci. China Chem.* **2022**, *65* (10), 1968–1977. DOI: 10.1007/s11426-022-1387-7.

[3] Krężel, A.; Bal, W. A Formula for Correlating pK_a Values Determined in D_2O and H_2O. *J. Inorg. Biochem.* **2004**, *98* (1), 161–166. DOI: 10.1016/j.jinorgbio.2003.10.001.

[4] (a) Newcomb, M.; Reeder, R. A. Reactions of *trans*-2-*tert*-butyl-3-phenyloxaziridine with lithium amide bases. *J. Org. Chem.* **1980**, *45* (8), 1489–1493. DOI:10.1021/jo01296a029. (b) Searles, S.; Li, Y.; Nassim, B.; Robert Lopes, M.-T.; T. Tran, P.; Crabbé, P. Observation on the Synthesis of Allenes by Homologation

of Alk-1-Ynes. *J. Chem. Soc., Perkin trans. 1* **1984**, *0* (0), 747–751. DOI: 10.1039/P19840000747. (c) Viciu, M. S.; Gupta, L.; Collum, D. B. Mechanism of Lithium Diisopropylamide-Mediated Substitution of 2,6-Difluoropyridine. *J. Am. Chem. Soc.* **2010**, *132* (18), 6361–6365. DOI: 10.1021/ja910834b.

[5] De-Long, K.; Huang, Y.; Ren, L. -Y.; Feng, W. -H. A Highly Efficient Way to Recycle Inactive Stereoisomers of Bedaquiline into Two Previous Intermediates via Base-Catalyzed C_{sp3}–C_{sp3} Bond Cleavage. *Chin. Chem. Lett.* **2015**, 26 (6), 790–792. DOI: 10.1016/j.cclet.2015.04.013.

[6] L'Estrade, E. T.; Edgar, F. G.; Xiong, M.; Shalgunov, V.; Baerentzen, S. L.; Erlandsson, M.; Ohlsson, T. G.; Palner, M.; Knudsen, G. M.; Herth, M. M. Synthesis, Radiolabeling, and in Vitro and in Vivo Evaluation of [^{18}F]ENL30: A Potential PET Radiotracer for the 5-HT_7 Receptor. *ACS Omega* **2019**, *4* (4), 7344–7353. DOI: 10.1021/acsomega.9b00394.

[7] Kurokawa, M.; Shindo, T.; Suzuki, M.; Nakajima, N.; Ishihara, K.; Sugai, T. Enzyme-Catalyzed Enantiomeric Resolution of *N*-Boc-Proline as the Key-Step in an Expeditious Route towards RAMP. *Tetrahedron: Asymmetry* **2003**, *14* (10), 1323–1333. DOI: 10.1016/S0957-4166(03)00210-6.

[8] Bootwicha, T.; Feilner, J. M.; Myers, E. L.; Aggarwal, V. K. Iterative Assembly Line Synthesis of Polypropionates with Full Stereocontrol. *Nature Chemistry* **2017**, *9* (9), 896–902. DOI: 10.1038/nchem.2757.

[9] (a) Ahlbrecht, H.; Enders, D.; Santowski, L.; Zimmermann, G. Chirale Homoenolat-Äquivalente, II: Asymmetrische Synthese 3-substituierter Phenylpropionaldehyde über metallierte chirale Cinnamylamine. *Chemische Berichte* **1989**, *122* (10), 1995–2004. DOI: 10.1002/cber.19891221027. (b) van Delden, R. A.; Hurenkamp, J. H.; Feringa, B. L. Photochemical and Thermal Isomerization Processes of a Chiral Auxiliary Based Donor–Acceptor Substituted Chiroptical Molecular Switch: Convergent Synthesis, Improved Resolution and Switching Properties. *Chemistry – A European Journal* **2003**, *9* (12), 2845–2853. DOI: 10.1002/chem.200204660.

[10] King, A. M.; Salomé, C.; Dinsmore, J.; Salomé-Grosjean, E.; Ryck, M. D.; Kaminski, R.; Valade, A.; Kohn, H. Primary Amino Acid Derivatives: Compounds with Anticonvulsant and Neuropathic Pain Protection Activities. *J. Med. Chem.* **2011**, *54* (13), 4815–4830. DOI: 10.1021/jm2004305.

[11] Gajula, P. K.; Asthana, J.; Panda, D.; Chakraborty, T. K. A Synthetic Dolastatin 10 Analogue Suppresses Microtubule Dynamics, Inhibits Cell Proliferation, and Induces Apoptotic Cell Death. *J. Med. Chem.* **2013**, *56* (6), 2235–2245. DOI: 10.1021/jm3009629.

[12] Li, F.; Long, L.; He, Y. -M.; Li, Z.; Chen, H.; Fan, Q. -H. Manganese-Catalyzed Asymmetric Formal Hydroamination of Allylic Alcohols: A Remarkable Macrocyclic Ligand Effect. *Angew. Chem., Int. Ed.* **2022**, *61* (26), e202202972. DOI: 10.1002/anie.202202972.

[13] Bera, N.; Samanta, S.; Sarkar, D. Stereoselective Synthesis of Oxacycles via Ruthenium-Catalyzed Atom-Economic Coupling of Propargyl Alcohols and Michael Acceptors. *J. Org. Chem.* **2021**, *86* (23), 16369–16395. DOI: 10.1021/acs.joc.1c01758.
